# Joint action of miR‐126 and MAPK/PI3K inhibitors against metastatic melanoma

**DOI:** 10.1002/1878-0261.12506

**Published:** 2019-08-06

**Authors:** Francesca Pedini, Gabriele De Luca, Federica Felicetti, Rossella Puglisi, Alessandra Boe, Maria Beatrice Arasi, Federica Fratini, Gianfranco Mattia, Massimo Spada, Simona Caporali, Mauro Biffoni, Alessandro Giuliani, Alessandra Carè, Nadia Felli

**Affiliations:** ^1^ Department of Oncology and Molecular Medicine Istituto Superiore di Sanità Rome Italy; ^2^ Center for Gender Medicine Oncology Unit Istituto Superiore di Sanità Rome Italy; ^3^ Core Facilities Istituto Superiore di Sanità Rome Italy; ^4^ Center of Animal Research and Welfare Istituto Superiore di Sanità Rome Italy; ^5^ Laboratory of Molecular Oncology Istituto Dermopatico dell'Immacolata‐IRCCS Rome Italy; ^6^ Department of Environment and Health Istituto Superiore di Sanità Rome Italy

**Keywords:** drugs, melanoma, miR‐126, synergistic therapy

## Abstract

Emerging data support the rationale of combined therapies in advanced melanoma. Specifically, the combined use of drugs with different mechanisms of action can reduce the probability of selecting resistant clones. To identify agents active against melanoma cells, we screened a library of 349 anti‐cancer compounds, currently in clinical use or trials, and selected PIK‐75, an inhibitor of the phosphatidylinositol 3‐kinase/protein kinase B (PI3K/AKT) pathway, as the ‘top active’ drug. PIK‐75 was then used alone or in combination with vemurafenib, the first BRAF inhibitor approved for patients with melanoma harboring BRAF mutations. We identified a combined dose of PIK‐75 and vemurafenib that inhibited both the PI3K/AKT and mitogen‐activated protein kinase pathways, thereby overcoming any compensatory activation. In view of the important tumor suppressor function induced by restoring expression of microRNA (miR)‐126 in metastatic melanoma cells, we examined whether miR‐126 has a synergistic role when included in a triple combination alongside PIK‐75 and vemurafenib. We found that enforced expression of miR‐126 (which alone can reduce tumorigenicity) significantly increased PIK‐75 activity when used as either a single agent or in combination with vemurafenib. Interestingly, PIK‐75 proved to be effective against early passage cell lines derived from patients’ biopsies and on melanoma cell lines resistant to either vemurafenib or dabrafenib, thus suggesting that it potentially has the capability to overcome drug resistance. Finally, the synergistic role played by miR‐126 in combination with vemurafenib and/or PIK‐75 was demonstrated *in vivo* in mouse xenograft models, in which tumor growth inhibition was associated with increased apoptosis. These results not only show the efficacy of PIK‐75 and vemurafenib co‐treatment but also indicate that restoration of miR‐126 expression in advanced melanoma can enhance their antitumor activity, which may possibly allow dose reduction to decrease adverse events without reducing the therapeutic benefits.

**Abbreviations**
BRAFi: BRAF inhibitors
EOB: excess over bliss
IC_50_: half maximal inhibitory concentration
IRS‐1: insulin receptor substrate 1
LC‐MS/MS: liquid chromatography‐tandem mass spectrometry
MAPK/ERK: mitogen‐activated protein kinase/extracellular signal‐regulated kinase
MAPK: mitogen‐activated protein kinase
miR: microRNA
MTV: median tumor volume
PARP: poly (ADP‐ribose) polymerase
PI3K/AKT: phosphatidylinositol 3‐kinase/protein kinase B
PI3Ki: PI3K inhibitors
Pt1, Pt2, Pt3 and Pt4: cell lines obtained from bioptic patient samples
qRT‐PCR: quantitative reverse transcription PCR
SD: standard deviation
TGI: tumor growth inhibition

## 1 Introduction

Cutaneous melanoma is a highly aggressive form of cancer whose incidence is constantly increasing worldwide (Apalla *et al*., 2017; Matthews *et al*., 2017). Although, it is surgically curable in its early phase, after dissemination, metastatic melanomas are characterized by poor prognosis and short survival of patients (Eggermont *et al*., 2014). In the last few years, a number of novel promising therapeutic agents have been developed, specifically including molecular targeting drugs and immune checkpoint inhibitors. Despite the impressive clinical responses, only a small fraction of patients obtain a durable response, and the emergence of acquired resistance appears a major problem for both treatments (Matthews *et al*., 2017; Nazarian *et al*., 2010). To overcome this failure, the emerging therapies are increasingly considering combinatorial treatments based on different mechanisms of action (Hepner *et al*., 2017; Shtivelman *et al*., 2014; Volpe *et al*., 2017).

One of the most relevant targeted proteins is BRAF, a Ser/Thr protein kinase mutated in 60–70% of melanoma patients, mostly as a V600E substitution (Davies *et al*., 2002). Mutated BRAF represents a constitutively active isoform which maintains the mitogen‐activated protein kinase (MAPK) pathway always activated (Cantwell‐Dorris *et al*., 2011). Vemurafenib, the first BRAF inhibitor (BRAFi) approved for BRAF‐mutated melanoma, induces a clinical response in a large proportion of patients with an overall response rate (RR) of up to 69%. Unfortunately, 90% of these patients develop resistance and undergo tumor progression within 6–8 months (Chan *et al*., 2017; George *et al*., 2017), in most cases because of the presence of additional mutations in the same (MAPK) or in other signaling pathways. Phosphatidylinositol 3‐kinase/protein kinase B (PI3K/AKT) is the second most frequently deregulated pathway in melanoma (Lim *et al*., 2017). Several inhibitors targeting the PI3K/AKT pathway (PI3Ki) have been developed and are now at different stages of clinical trials (Massacesi *et al*., 2016). Although some clinical activity has been observed with PI3Ki as a single agent, clinically significant results have not been observed. Conversely, several publications showed improved efficacy of PI3Ki used in combination with other agents (Tolcher *et al*., 2018). Indeed, possibly because of the complexity derived from molecule interactions and feedback loops, single‐agent therapies did not yield satisfactory results, with failures at least partly due to the significant functional cross‐talk existing between the MAPK and PI3K/AKT key pathways. In fact, inhibition of just one of these two signaling pathways often leads to a compensatory activation of the other, eventually interfering with adequate therapeutic responses (Pappalardo *et al*., 2016). Hence, there is a strong scientific rationale to explore the simultaneous targeting of more than one signaling pathway, including MAPK and PI3K/AKT (Lim *et al*., 2017; Smalley and Sondak, 2013). Moreover, regulation of gene expression at the post‐transcriptional level, such as that mediated by microRNA (miR), may interfere with the melanoma malignant phenotype. We previously showed the marked decline of miR‐126 and its complement miR‐126* (miR‐126‐3p and miR‐126‐5p, respectively, hereafter referred as to miR‐126) in metastatic cells, demonstrating their tumor suppressor function and proposing the restoration of their levels as a potential therapeutic option for the treatment of advanced melanoma (Felli *et al*., 2013, 2016). Interestingly, miR‐126 down‐modulation also plays a role in other neoplasms, where its restored expression significantly inhibits growth and dissemination as well as angiogenesis (Alhasan, 2019; Han *et al*., 2018; Liu *et al*., 2019). Indeed, miR‐126 expression was shown to be significantly associated with outcomes of patients with metastatic colorectal cancer receiving bevacizumab (Fiala *et al*., 2017) and reversal of resistance to TRAIL in cervical cancer (Zhang *et al*., 2017). In addition, miR‐126 serum amount has been demonstrated to represent a prognostic biomarker (Feng *et al*., 2018). Recently, a number of long noncoding RNA have been reported to favor cancer progression via miR‐126 down‐regulation (Huang *et al*., 2018; Sun *et al*., 2019; Wang *et al*., 2019).

Here, we have investigated the possibility of using miR‐126 as a sensitizing agent to increase the effects of targeted therapies against key signaling pathways such as MAPK/extracellular signal‐regulated kinase (ERK) and PI3K/AKT. Specifically, as an MAPK inhibitor, we selected vemurafenib, since it represents a standard melanoma first‐line treatment in BRAF‐mutated melanoma (Savoia *et al*., 2019). Conversely, PIK‐75 was identified among the more active drugs by screening an anti‐cancer drug library on metastatic melanoma cells.

## 2 Materials and methods

### 2.1 Antibodies

Anti‐pAKT (Ser473; #4058), anti‐AKT (#9272), anti‐pERK (Thr202/Tyr204; #4376), anti‐ERK1/2 (#9107), anti‐poly (ADP‐ribose) polymerase (PARP; #9532), anti‐caspase 3 (#9665) and anti‐insulin receptor substrate 1 (IRS‐1; #2382) antibodies were purchased from Cell Signaling (Danvers, MA, USA). Anti‐cyclin D1 (sc‐718) and anti‐p110δ (sc‐136032) antibodies were purchased from Santa Cruz Biotechnology (Dallas, TX, USA). Anti‐p85β (ab28356) was purchased from Abcam (Cambridge, UK). Anti‐α‐tubulin (T5168) and anti‐β‐actin (A5441; Sigma‐Aldrich Inc., St. Louis, MO, USA) antibodies were used as a loading control. All antibodies were used in accordance with the manufacturer's instructions.

For immunofluorescence analysis, polyclonal rabbit anti‐cleaved caspase 3 (Asp175; #9661) antibody was purchased from Cell Signaling and the monoclonal mouse anti‐human Ki‐67 antigen (Clone MIB‐1; M7240) was purchased from DAKO (Glostrup, Denmark).

### 2.2 Cell culture and transduction

The malignant human melanoma cell line A375M was kindly provided by R. Giavazzi (Istituto Mario Negri, Bergamo, Italy). The human melanoma cell lines A375 and SK‐Mel28 and the sublines A375‐R and SK‐Mel28‐R with acquired resistance to dabrafenib, and the early passage cell lines obtained from bioptic samples (reported as Pt1, Pt2, Pt3, Pt4) were obtained from S. D'Atri (IDI IRCCS, Rome, Italy) (Caporali *et al*., 2016, 2017). All biological materials were obtained with the informed written consent of patients and the study was conducted according to the Declaration of Helsinki Principles. The study methodologies were approved by the local ethics committee. Vemurafenib‐resistant A375M (A375M‐VR) cell lines (TripZ and miR‐126) were generated by culturing parental cells in increasing concentrations of vemurafenib (from 0.5 to 10 μm; Selleckchem, Munich, Germany) for at least 2 months and subsequently maintained in full growth medium containing 5 μm vemurafenib (Su *et al*., 2012). Overexpression of miR‐126 was obtained in melanoma cells using the doxycycline‐inducible lentiviral vector TripZ (Dharmacon, Lafayette, CO, USA), as already reported (Felli *et al*., 2013). After infection, transduced cells were selected with puromycin and red fluorescent protein level was evaluated by flow cytometry (FACSCanto; BD Biosciences, Milan, Italy) upon doxycycline induction (Sigma‐Aldrich). When indicated, the A375M‐VR melanoma cell line was treated with the proteasome inhibitor MG‐132 for 4 h at a final concentration of 20 μm (Sigma‐Aldrich). MiR‐126‐3p mimic and negative control expressions were obtained using 40 nm of miRIDIAN miR mimic (Product numbers #300626‐07 and #CN‐001000‐01; Dharmacon). MiR‐126‐3p silencing was performed using 200 nm of miRCURY locked‐nucleic‐acid (anti‐miR‐126‐3p). Power Inhibitor knockdown probe for miR‐126‐3p and negative control were obtained from Exiqon (Vedbaek, Denmark; Product numbers #426717‐00 and #199006‐00). Transfections were performed using Lipofectamine 2000 (Life Technologies, by Thermo Fisher, Waltham, MA, USA) according to the manufacturer's instructions. All melanoma cell lines were cultured in Dulbecco's modified Eagle's medium (Gibco by Life Technologies, Paisley, UK) supplemented with 10% FBS (Gibco). Cells were incubated at 37 °C with 5% CO_2_ in a humidified chamber. Cell lines were authenticated by standard short tandem repeat‐based genotyping (Banca Biologica e Cell Factory, IRCCS Azienda Ospedaliera Universitaria San Martino‐IST Istituto Nazionale per la Ricerca sul Cancro, Genoa, Italy).

### 2.3 Drug cytotoxicity experiments

For cytotoxicity assays, melanoma cell lines were plated in 96‐well plates at a density of 2 × 10^4 ^cells·mL^−1^ in triplicate. Anti‐cancer compound library (349 bioactive compounds), vemurafenib and PIK‐75 were purchased from Selleckchem. A complete list of the compounds used for the screening is available in Table S1. Compounds were dissolved in DMSO and added 48 h after cell plating. ATP levels were measured 24 and 48 h later, using CellTiter‐Glo™ (Promega Corporation, Madison, WI, USA) and quantified using an ELISA plate reader (VICTOR2; Perkin Elmer, Norwalk, CT, USA). During the experiments, cells were grown in the presence of doxycycline.

The excess over bliss (EOB) independence model was used to quantify the synergy for each compound pair. EOB evaluates the difference between the observed and expected inhibition of different dose drug combinations. Specifically, red squares indicate strong antagonistic effect, orange and light orange squares indicate slight antagonistic effect, dark blue indicates additive effect, and light green and dark green squares indicate slight and strong synergistic effects, respectively.

### 2.4 Luciferase assay

Luciferase reporter assays were performed as reported (Felli *et al*., 2013). Briefly, the 3′UTR of target genes (p110δ: NM_005026 from nt 3714 to nt 4088; p85β: NM_005027 from nt 3759 to nt 3958) predicted to interact with miR‐126, were PCR‐amplified and cloned in psiCHECKTM‐2 vector (Promega). For the luciferase reporter assay, each psiCHECKTM‐3′UTR plasmid was transfected in empty‐ or miR‐126‐vector‐transduced A375M cell line. Counts from empty vector‐transduced A375M were considered to be 100%.

### 2.5 Western blot

Total cell lysates were prepared by using Nonidet‐P40 lysate buffer (1% NP40, 200 nm NaCl, 50 mm Tris pH 7.4) plus protease and phosphatase inhibitors, maintained on ice for 30 min, vortexed and then centrifuged at 800 ***g*** for 10 min at 4 °C. Protein concentration was measured by BioRad protein‐assay (Hercules, CA, USA). Western blot was performed according to standard procedures. Total cell lysates were separated by the precast NuPAGE polyacrylamide gel system (Life Technologies by Thermo Fisher).

### 2.6 RNA extraction and real time quantitative reverse transcription PCR (qRT‐PCR)

Total RNA was extracted with NucleoSpin miRNA kit (Macherey‐Nagel GmbH & Co. KG. Düren, Germany) according to the manufacturer's specifications. Real time quantification qRT‐PCR was performed using the TaqMan technology (Applied Biosystems, Foster City, CA, USA: miR‐126 #000450; miR‐126* #000451). Samples were normalized by evaluating U6 small‐nuclear ribonucleoprotein (#001093) expression.

### 2.7 Apoptosis detection

Apoptosis was measured by flow cytometry. Cells showing a sub‐G0 DNA content were identified as apoptotic. For DNA staining, 2 × 10^4^ cells were plated in 24‐well microtiter plates. After 48 h, cells were treated with vemurafenib (500 nm) and PIK‐75 (40–60–80 nm) alone or in combination. After 24 h, cells were resuspended in Nicoletti's buffer containing propidium iodide 50 μg·mL^−1^. Samples were analyzed with FACSCanto flow cytometer (BD Biosciences) and data were analyzed with FACS diva software (Becton Dickinson, BD Biosciences, Milano, Italy).

### 2.8 Xenograft mouse models

Empty vector or miR‐126/126*‐transduced A375M cells were subcutaneously injected into the flanks of 5‐week‐old female nude mice (minimum *n* = 5 mice per group; Harlan, Udine, Italy) at a dose of 10^6^ cells mixed 1 : 1 with Matrigel (BD Biosciences) in 200 μL PBS. Treatments were initiated when xenografted tumors reached 30–60 mm^3^ and were administered daily intraperitoneally. Mice were randomly allocated to four treatment arms: PIK‐75 (2 mg·kg^−1^ DMSO : H_2_O), vemurafenib (20 mg·kg^−1^ DMSO : PBS), the combination (2 mg·kg^−1^ PIK‐75 plus 20 mg·kg^−1^ vemurafenib), and DMSO : H_2_O : PBS (control). Since PIK‐75 and vemurafenib are reversible inhibitors, they were administered five times per week to maintain stable inhibition. Tumor growth was monitored twice a week and diameters measured with calipers. Volumes were calculated using the following formula: volume (mm^3^) = (width)^2^ × length/2, where length represents the largest tumor diameter and width the perpendicular. Percentage of tumor growth inhibition (TGI) was calculated as the difference between the median tumor volume (MTV) of a treated group and the control group, using the following formula: %TGI = [(MTV_control_ − MTV_treated_)/MTV_control_] × 100. During the experiments, doxycycline was administered to the mice by daily oral gavage (10 mg·mL^−1^; Sigma‐Aldrich). Relative tumor growths were calculated by normalizing over initial tumor volume. Animal experiments were performed at the Istituto Superiore di Sanità (Rome, Italy) according to Italian law and institutional guidelines.

### 2.9 Immunofluorescence and apoptosis analysis on formalin‐fixed paraffin‐embedded tissue

Serial sections from mice tumor nodules embedded in paraffin were dewaxed and rehydrated. For immunolocalization studies, slides were subjected to heat‐mediated antigenic retrieval (10 mm sodium citrate buffer pH 6.0) and subsequently permeabilized (0.1% Triton X‐100 for 10 min) and saturated (3% BSA for at least 2 h) at room temperature. After incubation overnight at 4 °C in a humidified chamber with the primary antibodies, slides were incubated with specific fluorophore conjugated secondary antibodies (Alexa Fluor; Molecular Probes, Eugene, OR, USA) for 45 min at room temperature. Negative controls were performed by omission of the primary antibody in each experiment. Finally, slides were mounted with SlowFade anti‐fade reagent containing DAPI (Molecular Probes) and analyzed by Olympus F1000 laser‐scanning confocal microscopy (Olympus, Tokyo, Japan).

For apoptosis analysis, slides were subjected to TUNEL assay using *In situ* Cell Death Detection Kit, POD (Roche Italia, Monza, Italy). Very briefly, after digestion with Proteinase K (18 μg·mL^−1^ for 30 min at room temperature), permeabilization and endogenous peroxidase blocking, specimens were incubated with TUNEL reaction mixture for 1 h at 60 °C. Apoptotic positive nuclei were detected by incubation with Converter‐POD, followed by the colorimetric development of the 3,3′‐diaminobenzidine chromogenic substrate (Pierce by Thermo Fisher Scientific, Rockford, IL, USA). Finally, sections were counterstained with Hematoxylin & Eosin. Negative controls were performed by omission of TdT enzyme solution in the TUNEL mixture. Slides were evaluated using a Nikon Eclipse E1000 equipped with a Nikon DXM 1200 digital camera with dedicated acquisition software (Nikon ACT‐1 v. 2.1; all from Nikon Instruments, Campi Bisenzio, Florence, Italy).

### 2.10 Proteomic analysis and data processing

Proteins extracted from mice tumor were resolved by SDS/PAGE on precast 4–12% Bis‐Tris minigels (Life Technologies). Following staining with NuPage Colloidal Coomassie (Life Technologies), gel lanes were cut into 10–12 slices, and in‐gel tryptic digestion and liquid chromatography‐tandem mass spectrometry (LC‐MS/MS) were performed as already described (Boussadia *et al*., 2018). Data acquisition was performed in data‐dependent Top5. Spectra files were analyzed by Sequest HT search engine with proteome discoverer 1.4 (Thermo Fisher) using the Human Uniprot‐Swissprot database (released on June 2016) and containing also decoy database. The carbamidomethylation of cysteines was specified as a fixed modification, and the oxidation of methionine and phosphorylation of serine, threonine and tyrosine were set as variable modifications; mass tolerance was set to 1 Da for precursor ion and 0.4 Da for fragment ions; a maximum of two missed cleavages was allowed. The Percolator tool was used for peptide validation based on the q‐value and high confidence was chosen, corresponding to a false discovery rate ≤ 1% at peptide level. Proteins were identified with a minimum of one peptide rank = 1. Protein abundance was determined according to the label‐free Top3 method as already described (Fratini *et al*., 2017).

### 2.11 Pathway analysis

The enriched pathway analysis was performed using the david (http://david.abcc.ncifcrf.gov/) and panther (http://www.pantherdb.org/) tools. For the pathway analysis, the Kyoto Encyclopedia of Genes and Genomes (KEGG; https://www.genome.jp/kegg/pathway.html) database was selected.

### 2.12 Statistical analysis

Statistical analysis was performed using the *t*‐test with *P *< 0.05 deemed statistically significant. Unless otherwise stated, results were representative of at least three independent experiments.

Two‐way ANOVA analysis was performed using graphpad version 6.0 for Windows (San Diego, CA, USA) followed by Bonferroni *post hoc* test when appropriate.

## 3 Results

### 3.1 Screening of anti‐cancer compounds reveals high effectiveness PIK‐75 in melanoma growth inhibition *in vitro*

In view of the significant clinical effects obtained with BRAFi in melanoma patients carrying the BRAF^V600^ mutation, but bearing in mind that nearly all of them eventually progress, we were searching for additional drugs that, combined with vemurafenib, might be able to overcome drug resistance.

**Figure 1:**
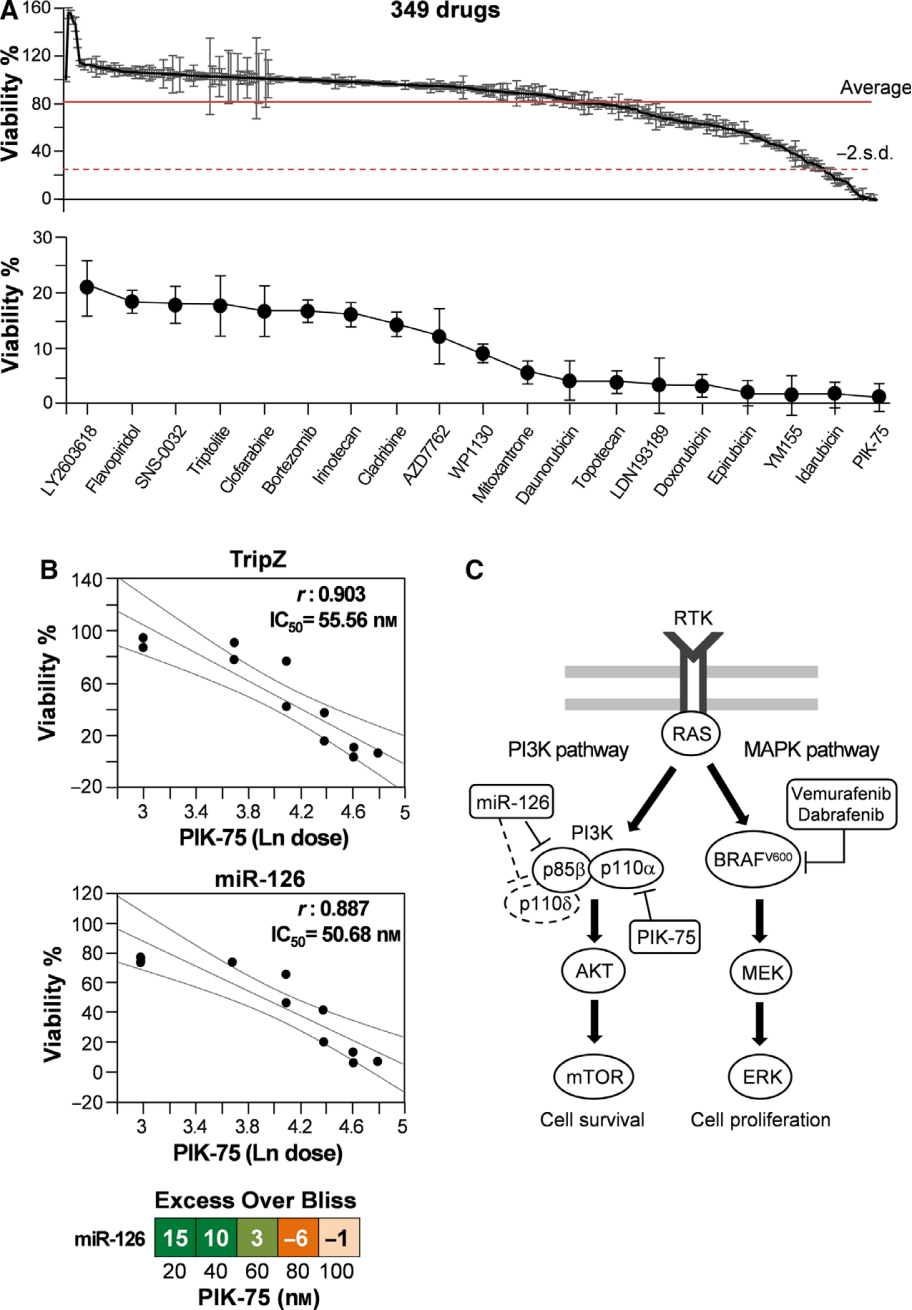
*In vitro* screening of 349 anti‐cancer library compounds. (A) Upper panel represents a point chart of the viability of the A375M melanoma cell line treated with the anti‐cancer compounds included in the library, used at 5 μm for 24 h. Normalized viability of each cell line is plotted as mean ± SD from three independent experiments. The average response is indicated by the solid line, and the dotted line outlines the value of 2 SD under the average. Bottom panel represents the point chart of the 19 compounds able to reduce the cell number below −2.0 × SD. (B) Dose‐effect responses of PIK‐75 treatment on A375M TripZ‐ and miR‐126‐transduced cells. EOB at each PIK‐75 concentrations relative to miR‐126 expression. Orange and light orange squares indicate slight antagonistic effect, light green and dark green squares indicate slight and strong synergistic effects, respectively. (C) Schematic PI3K and ERK signaling pathways in metastatic melanoma cells: p85β and p110δ subunits are targeted by miR‐126, PIK‐75 is a p110α inhibitor, and vemurafenib and dabrafenib are specific inhibitors of BRAF^V^ ^600^.

On these bases, we screened a library of 349 anti‐cancer compounds, including agents under evaluation in clinical trials or already approved for clinical use, on the BRAF^V600E^ A375M metastatic melanoma cell line. The initial screening strategy was based on single *in vitro* treatment of melanoma cells at high drug concentration (5 μm) and on the subsequent evaluation of cell vitality and proliferation. The primary end‐point was represented by the percentage of live cells, as evaluated through ATP quantification at 24 and 48 h after treatment in comparison with untreated controls. Despite the high dosages used, ~ 60% of the drugs (230 of 349 at 24 h and 195 at 48 h) did not produce significant effects, leaving alive more than 80% of the treated cells (Fig. 1A, top panel, Table S2). Based on these results, we selected the 19 drugs able to reduce the cell number below −2.0 × standard deviations (SD) from the overall mean viability at 24 h compared with the untreated counterparts (Fig. 1A, bottom panel). As our previous data showed the tumor suppressor role of miR‐126 in melanoma (Felli *et al*., 2013), we evaluated the possibility that miR‐126 forced expression might sensitize melanoma cells, potentiating the effects of chemical agents, thus indicating miR‐126 restoration as a potential therapeutic option for advanced melanoma. For this reason, a concentration‐response analysis was performed with the 19 selected chemical agents on both A375M control and miR‐126‐transduced cells. As first step, qRT‐PCR confirmed miR‐126 overexpression in miR‐ vs TripZ‐transduced melanoma cells. Specifically, we obtained ~ 14‐fold increase of miR‐126‐3p and ~ 4‐fold increase of miR‐126‐5p after 48 h of induction with doxycycline (Fig. S1). Different starting doses were then chosen for each drug based on the vitality measures obtained in the initial screening, finding in most cases the expected dose‐response curves (see Fig. 1B for PIK‐75 and Fig. S2 for other drugs). Among the compounds analyzed, PIK‐75 was shown as the most effective drug and, even more important, the only displaying a synergistic effect with miR‐126. In fact, although similar levels of vitality were displayed by ‘drug’ and ‘drug plus miR‐126’ treated cells, being the PIK‐75 half maximal inhibitory concentration (IC_50_) 55.05 and 50.68 nm, respectively, the EOB values related to lower doses of PIK‐75 (20 and 40 nm) indicated a significant synergistic effect (Fig. 1B, bottom panel).

### 3.2 Identification of miR‐126, PIK‐75 and vemurafenib synergistic combination for BRAF‐mutated melanoma cell line treatment

Mitogen‐activated protein kinase and PI3K/AKT/mTOR pathways represent the key signaling cascades involved in melanoma development and progression (Fig. 1C). As already reported (Xiao *et al*., 2016), miR‐126‐3p directly targets and represses the PI3KR2 (p85β) subunit of PI3K. Moreover, bioinformatic‐based approaches (TargetScan, PicTar and RNA hybrid) indicated PI3KCD (p110δ) as an additional putative target of miR‐126. To demonstrate this hypothesis, we performed a reporter luciferase assay by cloning the p110δ 3′UTR downstream of the Renilla open reading frame in the pSiCheck vector. The reporter construct was transfected into the A375M melanoma cell line stably transduced to overexpress miR‐126 or in control cells transduced with the empty vector. Results indicated a significant miR‐126‐dependent inhibition of the luciferase activity, demonstrating its targeting of p110δ. Western blot analysis confirmed the down‐regulation of both p85β and p110δ associated with miR‐126 overexpression in the A375M cell line (Fig. S3). Interestingly, PIK‐75 plays a regulatory role on the same pathway through the direct repression of the p110α (Zheng *et al*., 2011). Conversely, vemurafenib is a small molecule, which inhibits the constitutively active MAPK cascade by targeting mutant BRAF. The potentially convergent action of these agents, acting either on the same or on interconnected pathways, prompted us to focus our attention on their possible combination to obtain a strong and not easy to circumvent therapeutic effect.

**Figure 2:**
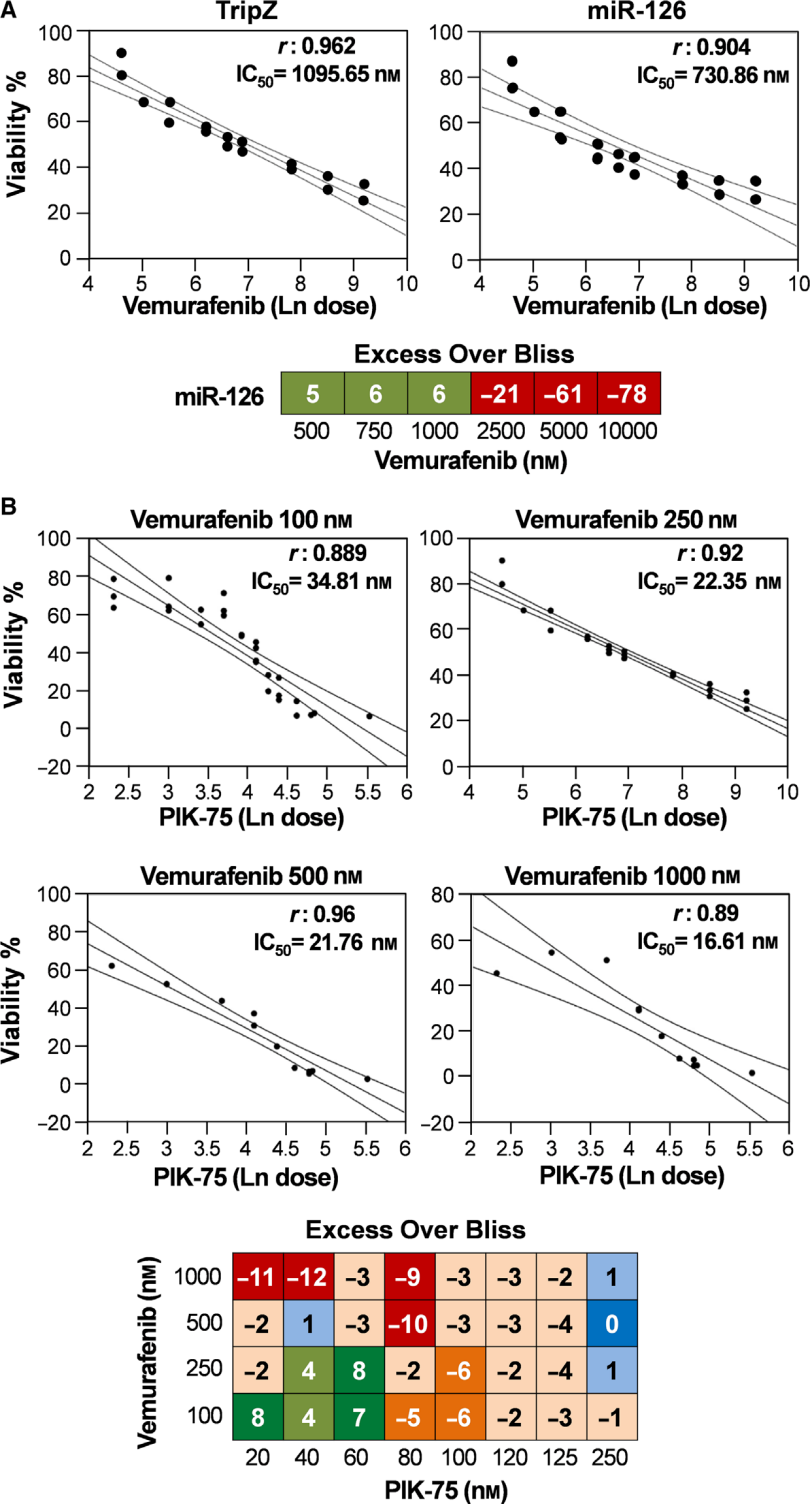
Vemurafenib treatment and combination of PIK‐75 and vemurafenib treatment. (A) Dose‐effect responses for vemurafenib on A375M TripZ and miR‐126. EOB at each vemurafenib concentrations relative to miR‐126 expression. Red squares indicate strong antagonistic effect and light green squares indicate slight synergistic effects, respectively. (B) Dose‐effect correlation of PIK‐75 in combination with different doses of vemurafenib. EOB at each drug concentration, including eight combinations of PIK‐75 and 4 of vemurafenib. Red squares indicate strong antagonistic effect, orange and light orange squares indicate slight antagonistic effect, dark blue indicates additive effect, light green and dark green squares indicate slight and strong synergistic effects, respectively.

After proving a PIK‐75 and miR‐126 synergistic effect (Fig. 1B), we demonstrated in *in vitro* experiments that the anti‐cancer activity played by vemurafenib was enhanced by the overexpression of the oncosuppressor miR‐126, with an IC_50_ of 730.86 nm for vemurafenib treatment of A375M/miR‐126 compared with 1095.65 nm for control A375M cells. Again, at the lower doses of vemurafenib, the EOB results showed the effectiveness of the ‘drug plus miR’ treatment (Fig. 2A). Proceeding to evaluate the efficacy of the combinatorial approach of anti‐cancer drugs and looking for a treatment capable of overcoming the acquired resistance associated with BRAFi in the majority of melanoma patients, we focused on the possible combination of PIK‐75 with vemurafenib. The possible therapeutic approaches were tested on A375M viability at multiple concentrations, according to a model in which each drug was supplemented either as single agent or in combination. The inhibitory effects were evaluated on cell proliferation and viability according to the Bliss independence model. On these bases, we obtained high synergistic scores with low doses of both drugs: 100 and 250 nm for vemurafenib combined with doses of PIK‐75 ranging between 20 and 60 nm (Fig. 2B).

To better dissect the synergistic results obtained by overexpressing the miR‐126 precursor, the sole miR‐126‐3p mimic sequence was transfected in the A375M cell line, looking for the specificity of its role in increasing the drug‐dependent actions. Indeed, significant enhancement of PIK‐75 and vemurafenib effects, demonstrated by reduced cell viability, was observed in single or combined treatments on A375M cells transiently transduced with miR‐126‐3p mimic (Fig. S4A,B, right graph). The functionality of miR‐126‐3p in mediating the combined action of PIK‐75 and vemurafenib was confirmed by the sensible reduction of drug effects observed after its silencing (Fig. S4B, left graph). The A375M cell line, selected for its low but detectable level of miR‐126 (Felli *et al*., 2013), represented a good cellular system for both overexpression or silencing experiments.

### 3.3 Effect of single drug treatments on the expression levels of PI3K/AKT and MAPK key proteins

**Figure 3:**
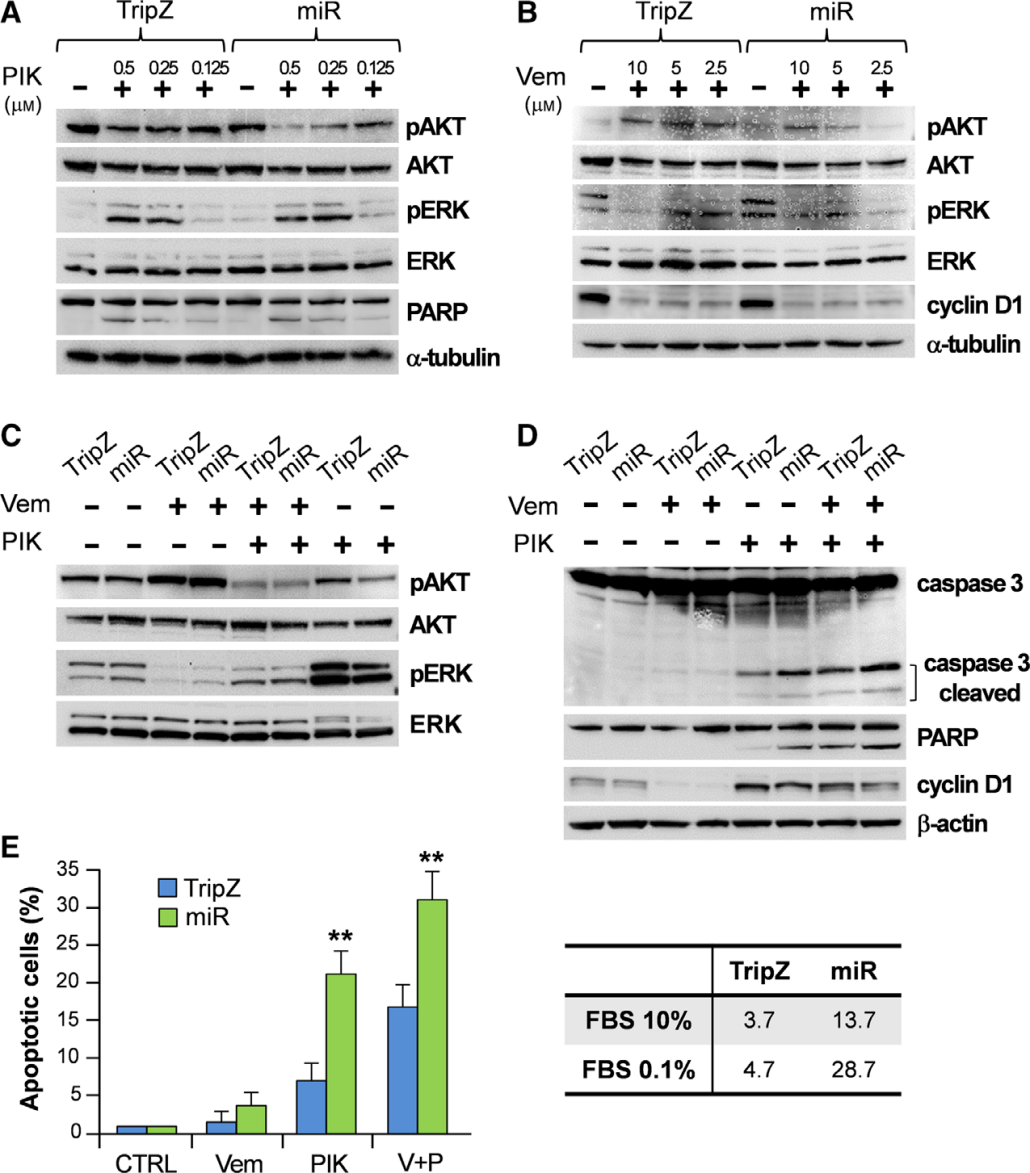
Synergistic effects of vemurafenib/PIK‐75/miR‐126 on the PI3K/AKT and MAPK pathways evaluated in the A375M‐TripZ (TripZ) vs A375M/miR‐126 (miR) cell lines. Representative western blots illustrate the patterns of AKT and ERK phosphorylation, PARP and cyclin D1 expression in melanoma cells exposed to decreasing concentrations of (A) PIK‐75 (PIK; 0.5–0.125 μm) and (B) vemurafenib (Vem; 10–2.5 μm) for 5 h, and the expression levels of (C) AKT and ERK phosphorylation and (D) caspase 3, PARP and cyclin D1 in melanoma cells exposed to vemurafenib (Vem; 5 μm) or PIK‐75 (PIK; 0.5 μm) as single agents or in combination for 5 h. (E) Quantification of apoptotic cell fractions in TripZ and miR‐126 after 24 h treatment with vemurafenib (Vem; 500 nm) or PIK‐75 (PIK; 80 nm) alone or in combination (V + P). Columns, mean ± SD of at least three independent experiments (left panel). Differences were statistically evaluated by Student's *t*‐test, **P < 0.01. Percentages of apoptotic cells in TRIPZ and miR‐126 control cells cultured in 10% or 0.1% FBS (right panel). α‐Tubulin or β‐actin was used as protein loading control. V + P: vemurafenib + PIK‐75.

To evaluate the action of PIK‐75 and vemurafenib, administered as single drugs, we performed a series of short time *in vitro* treatments on both A375M control (A375M/TripZ empty vector) and miR‐126 overexpressing cells, using increasing doses of either PIK‐75 (0.125, 0.25 and 0.5 μm) or vemurafenib (2.5, 5 and 10 μm). After 5 h, cells were harvested and analyzed by western blot for both AKT and ERK phosphorylation levels. Results showed that PIK‐75 inhibited AKT phosphorylation in a concentration‐dependent manner and that this effect was significantly more marked in A375M/miR‐126 than in control cells (Fig. 3A), in line with a direct action of miR‐126 on the PI3K/AKT pathway. PIK‐75 treatment also caused the cleavage of PARP, a well‐known marker of the apoptotic process and the contextual induction of ERK phosphorylation (Fig. 3A). In parallel experiments, vemurafenib induced a concentration‐dependent inhibition of ERK phosphorylation and a consequent cyclin D1 down‐modulation with the contemporary induction of AKT phosphorylation, without clear differences associated with miR‐126 enforced expression (Fig. 3B). These experiments confirmed in our cellular system the expected effects on these key signaling pathways, showing the crosstalk between PI3K/AKT and MAPK pathways and driving the strong increase of pERK when the drug treatment inhibited pAKT and *vice versa* (Fig. 3A,B). Quantitation of western blot images is reported in Fig. S5A,B.

### 3.4 Effective conditions for dual targeting of PI3K/AKT and MAPK pathways

In view of such a crosstalk between PI3K/AKT and MAPK and looking for the possibility of successfully inhibiting both pathways, we investigated several different PIK‐75 and vemurafenib dose combinations. Considering the results obtained by single administration of 0.25 μm PIK‐75 and 2.5 μm vemurafenib, the former able to decrease pAKT in the A375M/miR‐126 cells and the latter pERK in both A375M/TripZ and A375M/miR‐126 cell lines, we initially selected this combination. However, at these concentrations, the co‐administration of PIK‐75 and vemurafenib was not able concurrently to inhibit AKT and ERK phosphorylation (Fig. S6A,B). PIK‐75 and vemurafenib were then used at 0.5 and 5 μm, either as a single drug or in combination. The single agent‐based treatments with 0.5 μm PIK‐75 or 5 μm vemurafenib confirmed their inhibitory effects on AKT or ERK phosphorylation, respectively. Particularly evident was the reduced phosphorylated fraction of AKT in miR‐126 overexpressing cells treated with PIK‐75. More important, a significant synergistic result was obtained by the combination as, at these concentrations, the two drugs were capable of inhibiting both the PI3K/AKT and MAPK pathways, simultaneously preventing the compensatory effect usually observed (Fig. 3C). The capacity of these treatments to induce apoptosis was assessed by evaluating the amounts of cleaved PARP and caspase 3. Indeed, PIK‐75 administration was confirmed to induce apoptosis strongly and this effect was potentiated by the combined treatment. As expected (see Welsh *et al*., 2016), cyclin D1 was down‐modulated by vemurafenib, in line with the reduction of ERK phosphorylation, whereas it was increased by PIK‐75 (Fig. 3D). Quantitation of western blot images is reported in Fig. S5C,D.

Of note, the main effects obtained by PIK‐75 treatment, i.e. the reduced AKT phosphorylation and the induction of apoptosis, were more pronounced in miR‐126 overexpressing cells. Flow cytometry‐based apoptosis quantification in A375M/miR‐126 cells treated by PIK‐75 or PIK‐75 plus vemurafenib indicated the presence of a significantly higher percentage of apoptotic cells when compared with the A375M/TripZ cells treated with PIK‐75 (*P* < 0.01) or PIK‐75 plus vemurafenib (*P* < 0.01; Fig. 3E, left panel). According to our previous results (Felli *et al*., 2013), we further substantiated the capability of miR‐126 enforced expression to favor the apoptotic process of the A375M metastatic melanoma cell line at 0.1% serum culture conditions, even without any drug treatment. As shown in a representative analysis (Fig 3E, right panel), the percentage of apoptosis in A375M/miR‐126 cells kept in low serum was 28.7% compared with 4.7% of control cells. This difference was visible, although less marked, in more permissive culture conditions associated with 10% serum (13.7% vs 3.7% of apoptotic cells in miR‐126 and TripZ A375M cells, respectively).

### 3.5 Vemurafenib, PIK‐75 and miR‐126 triple combination showed a strong synergy on a vemurafenib‐resistant cell line

The suppression of MAPK signaling by BRAFi, like vemurafenib, is a therapeutic strategy widely employed in BRAF^V600^‐mutant melanomas; although very high RRs are obtained, up to 90% of vemurafenib‐responding patients develop resistance. Trying to overcome such resistance development, several dual therapies have been approved (Tolcher *et al*., 2018) and now represent the standard of care.

To evaluate the efficacy of the triple combination vemurafenib/PIK‐75/miR‐126 in cells resistant to vemurafenib, we established a resistant sub‐line (A375M‐VR) by continuous exposure of the parental A375M cell line to increasing concentrations of vemurafenib up to the final 10 μm. After selection, the A375M‐VR cells were lentivirally transduced to overexpress miR‐126. qRT‐PCR‐confirmed miR‐126 overexpression in miR‐ vs empty vector‐transduced melanoma cells. Specifically, we obtained ~ 13‐fold increase of miR‐126‐3p and ~ 3‐fold increase of miR‐126‐5p after 48 h of doxycycline induction (Fig. S7). The persistence of BRAF^V600E^ activating mutation in A375M‐VR was verified by sequencing.

**Figure 4:**
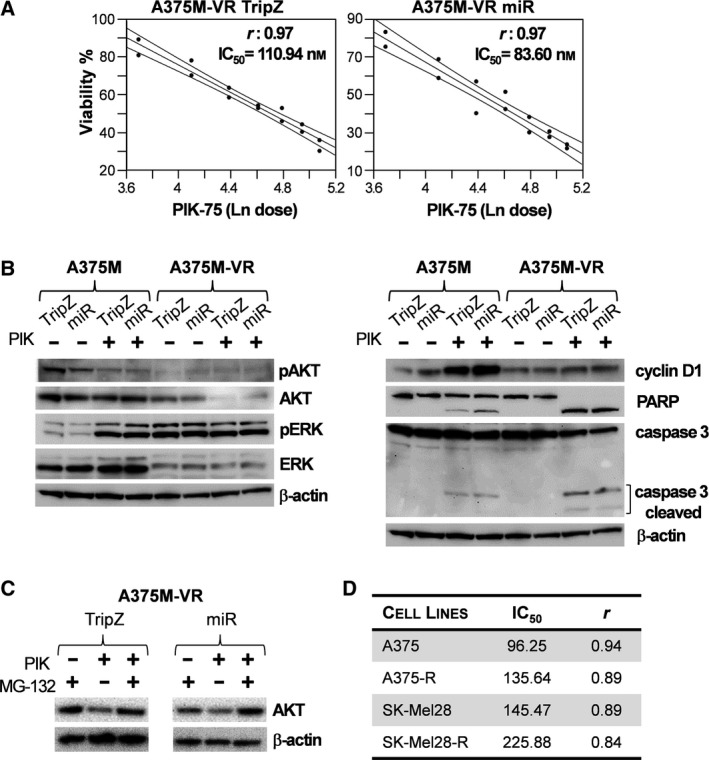
PIK‐75 effect on A375M‐VR cell lines. (A) Synergistic effects of PIK‐75 and miR‐126 on melanoma A375M‐VR cell line. Dose‐response of PIK‐75 on A375M‐VR/TripZ (left panel) and A375M‐VR/miR‐126 (right panel). Western blot analysis of (B) AKT and ERK phosphorylation, cyclin D1, PARP and caspase 3 proteins levels in A375M and A375M‐VR cell lines treated or not treated with PIK‐75 (PIK; 0.5 μm) and (C) AKT expression on A375M‐VR cell line treated with PIK‐75 (PIK) in the presence or absence of the proteasome inhibitor MG‐132 (20 μm) for 4 h. (D) IC_50_ (nm) of melanoma dabrafenib‐resistant cell lines (A375‐R, SK‐Mel28‐R). Legend: PIK‐75: 50 nm ≤ PIK‐75 ≤ 250 nm. β‐Actin was used as protein loading control.

Cell viability assays showed a similar pattern of response to PIK‐75 treatment in A375M‐VR and A375M parental cell lines. Interestingly, a consistent improvement was detected in A375M‐VR cells overexpressing miR‐126 treated with PIK‐75 (IC_50_ was 83.60 nm for A375M‐VR/miR‐126 vs 110.94 nm for A375M‐VR/TripZ; Figs 1B and 4A).

We then analyzed how the acquisition of resistance to vemurafenib affected PI3K/AKT and MAPK pathways with or without miR‐126 enforced expression. The first unexpected result was the detection of the very low level of total AKT in A375M‐VR compared with the A375M counterpart cells either untreated or treated with PIK‐75 (Fig. 4B, left panel). To assess whether this result might derive from a different AKT protein stability, we performed the PIK‐75 treatment in the presence of the proteasome inhibitor MG‐132. As shown in Fig. 4C, we obtained a complete MG132‐dependent rescue of AKT protein in both control and miR‐126 cells treated with PIK‐75, suggesting a significant proteasome‐dependent degradation of AKT in this condition.

In PIK‐75‐treated A375M‐VR cells, expressing or not expressing miR‐126, we observed caspase 3 activation and PARP cleavage at levels significantly higher than those observed in control A375M cells, suggesting that the apoptotic process induced by PIK‐75 was stronger in A375M‐VR. Finally, probably due to the onset of escape mechanisms of adaptation to chronic vemurafenib exposure, the fraction of phosphorylated ERK was originally very high and was not further increased by PIK‐75. Unexpectedly, the high level of cyclin D1 observed in A375M contextual to pERK‐increase cells was not visible in A375M‐VR cells (Fig. 4B, right panel). Quantitation of western blot images is reported in Fig. S8A–C.

### 3.6 PIK‐75 inhibited cell viability in dabrafenib‐resistant melanoma cell lines

To confirm the wider effectiveness of PIK‐75 on cell growth viability, we evaluated its action on melanoma cell lines resistant to dabrafenib compared with their parental counterparts. Dabrafenib, like vemurafenib, inhibits BRAF and is used for metastatic melanoma therapy. Two metastatic melanoma cell lines, A375 and SK‐Mel28, were grown at increasing concentrations of dabrafenib up to their final selection (Caporali *et al*., 2016, 2017). Cell viability was evaluated on both parental (A375 and SK‐Mel28) and dabrafenib‐resistant sublines (A375‐R and SK‐Mel28‐R). As shown in Fig. 4D, PIK‐75 was able to inhibit A375 and SK‐Mel28 cell growth (IC_50_ 96.25 and 145.47 nm, respectively) as well as A375‐R and SK‐Mel28‐R sublines (IC_50_ 135.64 and 225.88 nm, respectively), thus further suggesting this drug as a good candidate for a dual therapeutic application.

### 3.7 Combination of PIK‐75 and vemurafenib showed a strong synergy in metastatic melanoma cells derived from patients

**Figure 5:**
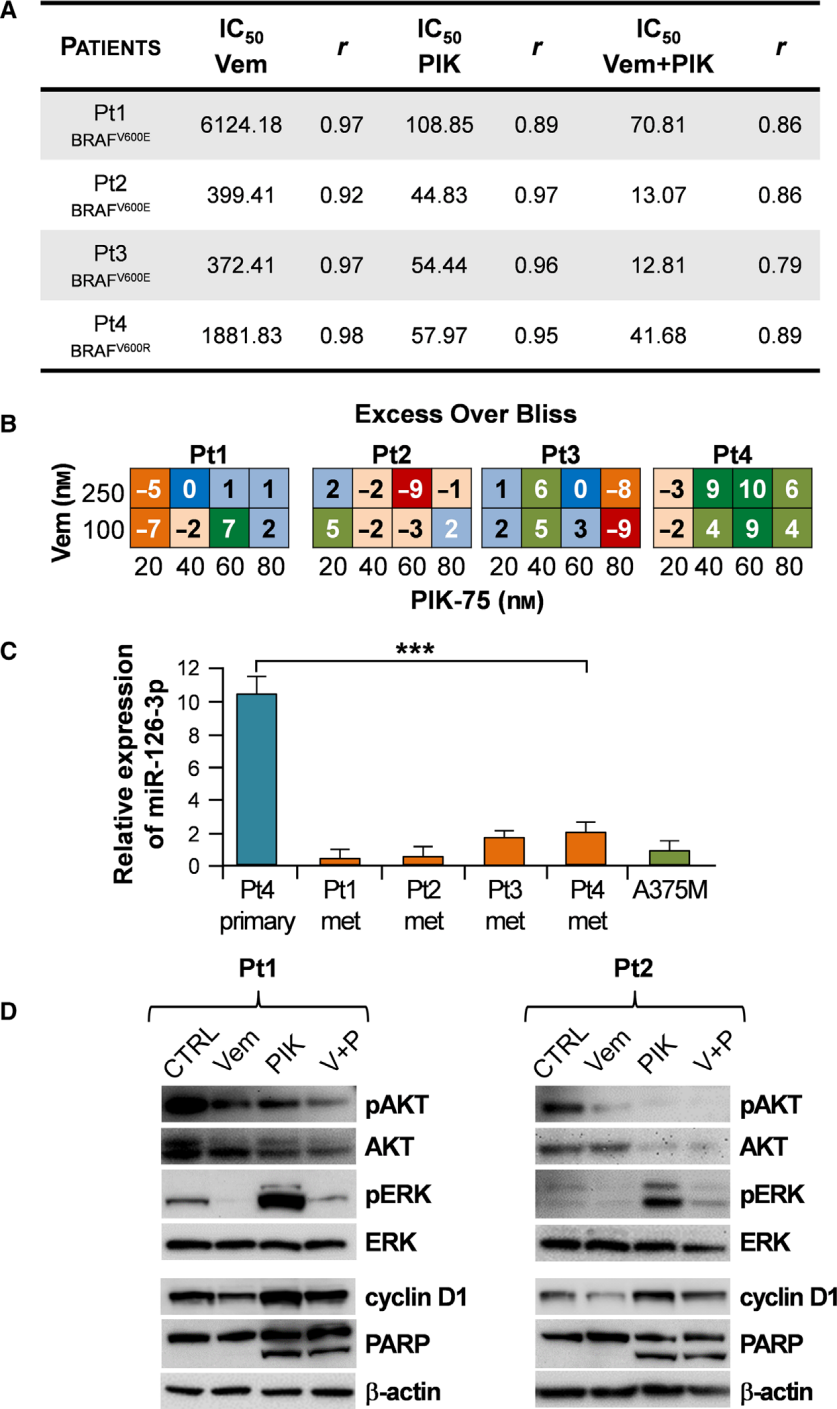
Effect of PIK‐75 and vemurafenib treatments on cell lines derived from metastatic patients. (A) IC_50_ (nm) of cells from patients Pt1, Pt2, Pt3 (BRAF^V^ ^600E^) and Pt4 (BRAF^V^ ^600R^). Legend: Vemurafenib (Vem): 100 nm ≤ Vem ≤ 10 000 nm; PIK‐75 (PIK): 20 nm ≤ PIK‐75 ≤ 120 nm; vemurafenib + PIK‐75 (V + P): vem 250 nm + 20 nm ≤ PIK‐75 ≤ 120 nm. (B) EOB at each drug concentration relative to drug effects on patients (Pt1, Pt2, Pt3 and Pt4). Red squares indicate strong antagonistic effect, orange squares strong slight antagonistic effect, light orange squares slight antagonistic effect, blue square additive effect, light blue squares slight synergistic effect, light green squares strong slight synergistic effect, and green squares strong synergistic effect. (C) qRT‐PCR analysis illustrating the expression levels of miR‐126 on metastatic melanoma patients (Pt1, Pt2, Pt3 and Pt4). Primary Pt4 cell line and A375M were used as controls. Columns, means ± SD of at least three independent experiments. Differences were statistically evaluated by Student's *t*‐test, ***P <* *0.001. (D) Representative western blots illustrate the evolution of AKT and ERK phosphorylation and PARP and cyclin D1 expression in two cell lines derived from metastatic melanoma patients (Pt1 and Pt2) exposed to vemurafenib (Vem; 5 μm) or PIK‐75 (PIK; 0.5 μm) as a single agent or in combination for 5 h. β‐Actin was used as protein loading control. V + P: vemurafenib + PIK‐75.

As a last step, to strengthen our results, we selected four primitive cell lines derived from metastatic melanoma patients, three (Pt1, Pt2, Pt3) harboring the commonest BRAF mutation (V600E) and one (Pt4) the V600R BRAF mutation. The effects of vemurafenib and PIK‐75, as either single or combined treatments, were evaluated on cell viability. As reported in Fig. 5A, whereas the sensitivity to vemurafenib as single agent was extremely diversified (IC_50_ from 372.41 to 6124.18 nm), PIK‐75 produced a strong effect regardless of the type of BRAF mutation. Of note, vemurafenib was synergistic with PIK‐75, as it considerably reduced the PIK‐75 IC_50_ in all patients’ derived cell lines as shown by the values of EOB, particularly for Pt3 and Pt4 (Fig. 5B). Considering miR‐126 replacement as a possible therapeutic option, we evaluated the expression level of miR‐126‐3p in these patients’ derived metastatic cell lines (Pt1, Pt2, Pt3, Pt4) by comparing its amount with that expressed by the primary tumor counterpart of Pt4 and the A375M cell line. As expected, the expression level of miR‐126‐3p in metastatic cells was markedly lower than in primary tumor cells and similar to that of A375M (Fig. 5C).

We then selected Pt1 and Pt2 for further analysis because, showing a very different sensitivity to vemurafenib (IC_50_ 6124.18 and 399.41 nm, respectively), they could represent the paradigmatic condition of melanoma patients: initial disease regression after BRAFi treatment (Pt2) and subsequent appearance of relapse due to development of resistance (Pt1). After 5 h of drug treatment, we used western blot to analyze modulation of PI3K/AKT and ERK pathways, PARP cleavage and cyclin D1 regulation in these two primitive cell lines (Fig. 5D). Cells from patient Pt1 (less sensitive to vemurafenib) showed a high level of pERK at basic condition, as already observed in A375M‐VR cells. Vemurafenib inducing down‐modulation of pERK was detected in Pt1 and cyclin D1 in both cell lines (Pt1 and Pt2). As expected, PIK‐75 caused a strong cleavage of PARP with induction of ERK phosphorylation, and only the combined treatment, vemurafenib + PIK‐75, was able to maintain low levels of both pAKT and pERK and the efficient cleavage of PARP protein (Fig. 5D). Densitometric quantitation of western blot images is reported in Fig. S9A,B.

### 3.8 *In vivo* studies

**Figure 6:**
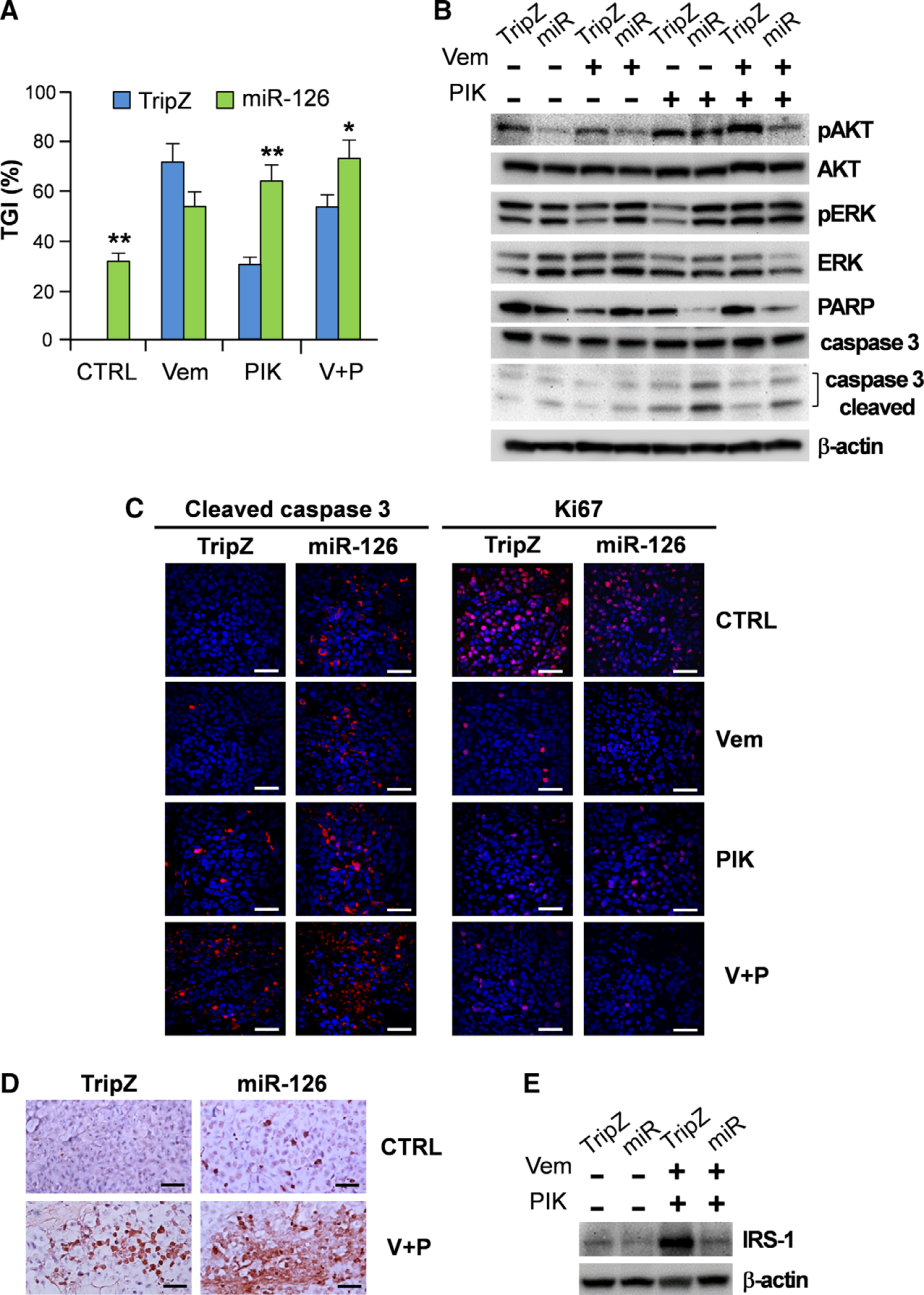
Effect of combined treatment with vemurafenib and PIK‐75 on *in vivo* tumor growth. (A) TGI determined at 21 days in A375M/TripZ (TripZ) and A375M/miR‐126 (miR) mice treated with vehicle, vemurafenib (20 mg·kg^−1^ daily) or PIK‐75 (2 mg·kg^−1^ daily) alone or in combination. Differences were analyzed by means of 2‐way ANOVA with Bonferroni post‐test and showed statistical significances. Columns, mean ± SD of two independent experiments (*P <* *0.05; **P < 0.01). (B) Western blot analysis performed to detect AKT and ERK phosphorylation, as well as PARP protein levels in xenograft tumors after vemurafenib and/or PIK‐75 treatment. (C) Confocal immunofluorescence analysis with anti‐cleaved caspase 3 and anti‐Ki67 antibodies on tumor treated with vemurafenib and PIK‐75 alone or in combination. Scale bar: 50 μm. (D) TUNEL staining on the *in vivo* xenograft model. At the end of the experiments, tumor tissues were processed for immunohistochemical TUNEL assay to detect apoptosis *in vivo*. Scale bar: 50 μm. (E) Western blot analysis performed to detect IRS‐1 in miR‐126‐transduced A375M melanoma compared with TripZ control cells. TripZ: A375M/TripZ; miR: A375M/miR‐126. β‐Actin was used as protein loading control. Vem: vemurafenib, PIK: PIK‐75, V + P: vemurafenib + PIK‐75.

The possible synergistic role played by miR‐126 in combination with vemurafenib and/or PIK‐75 therapeutic administrations was evaluated *in vivo* in mouse xenograft models. Athymic nude mice were injected with A375M/miR‐126 cells or A375M/TripZ control cells and the growth of tumor nodules followed over ~ 4 weeks. When xenograft tumors became palpable, mice were randomly allocated to four arms of treatment consisting in: daily intraperitoneal injection of PIK‐75 (2 mg·kg^−1^); vemurafenib (20 mg·kg^−1^); their combination; or the vehicle. The effects of the different conditions were evaluated as TGI% compared with mice inoculated with A375M/TripZ. After 3 weeks, in the vehicle group, tumor volumes derived from A375M/miR‐126 cells were significantly smaller than those from A375M/TripZ, with 32% of TGI, thus confirming the tumor suppressor function of this miR. An important synergistic effect was observed between miR‐126 enforced expression and PIK‐75 administration, used either as single agent (TGI: 64% in A375M/miR‐126‐derived tumors vs 30% in A375M/TripZ‐derived tumors, *P* < 0.01) or combined with vemurafenib (TGI: 73% in A375M/miR‐126‐derived tumors vs 53% in A375M/TripZ‐derived tumors, *P* < 0.05; Fig. 6A). Conversely, no significant differences were determined by vemurafenib monotherapy in tumors derived from A375M/TripZ control cells or from A375M/miR‐126 cells. During the experiments, no mice in any group lost bodyweight and no major abnormalities were observed in the sacrificed mice, indicating the absence of general toxicity due to the treatments. To confirm the inhibitory effects observed *in vitro* on PI3K/AKT and MAPK pathways, we analyzed by western blot the key molecules associated with these pathways in the excised xenograft tumors (Fig. 6B). Results confirmed that overexpression of miR‐126 was able to reduce the level of AKT phosphorylation in all the observed conditions. In agreement with *in vitro* results, we also observed a strong reduction of the uncleaved PARP protein and an increase of the activated caspase 3, both markers of the apoptotic process, only in PIK‐75 treated mice, which corresponds well to the overall TGI results (Fig. 6A,B). No reduction of ERK phosphorylation was observed (Fig. 6B). Quantitation of western blot images is reported in Fig. S10A.

In line with all these findings, immunofluorescence analysis on serial tumor slides highlighted that the amount of activated caspase 3 clearly correlated with PIK‐75 administration and miR‐126 overexpression. Caspase 3 activation was even more evident in A375M/miR‐126‐derived tumors of mice cotreated with PIK‐75 and vemurafenib (Fig. 6C, left panel). These findings were confirmed by TUNEL analysis, which showed a significantly enhanced apoptosis in the triple treatment, i.e. miR‐126 enforced expression plus vemurafenib‐PIK‐75 co‐administration (Figs 6D and S10B). An important observation was that, although the effects observed in A375M/miR‐126‐derived tumors treated with vemurafenib seemed poorer in terms of growth inhibition with respect to control cells (Fig. 6A), we detected higher amounts of apoptosis associated with miR‐126 overexpression, as indicated by caspase 3 activation (Fig. 6C, left panel). The evaluation of Ki67, as a marker of cell proliferation, showed a significant reduction of cell growth in the xenograft tumors of all treated groups compared with controls (Figs 6C, right panel, and S10B).

Finally, as an insulin feedback was reported to limit the efficacy of PI3K inhibition in several tumor models (Hopkins *et al*., 2018), we checked for the effects of the combined PIK‐75 plus vemurafenib therapy on the expression of the IRS‐1. IRS‐1, a key molecule in signal transduction between the insulin receptor and PI3K, was demonstrated to be a target gene of miR‐126 in different cancers (Fang *et al*., 2017). Expression studies, besides confirming IRS‐1 down‐regulation in miR‐126‐transduced A375M melanoma compared with TripZ control cells, showed the strong induction of IRS‐1 in PIK‐75 + vemurafenib cotreated A375M cells, a result suggestive of the association of its activation with drug resistance. Indeed, no up‐regulation of IRS‐1 was detected in miR‐126‐overexpressing A375M cell line, thus indicating that the miR‐126‐dependent inhibition might represent an alternative therapeutic strategy to interfere with the activation of the IRS‐1/PI3K/AKT pathway (Figs 6E and S10C).

### 3.9 Proteome profiling of A375M/TripZ‐ and A375M/miR‐126‐derived xenograft tumors

**Figure 7:**
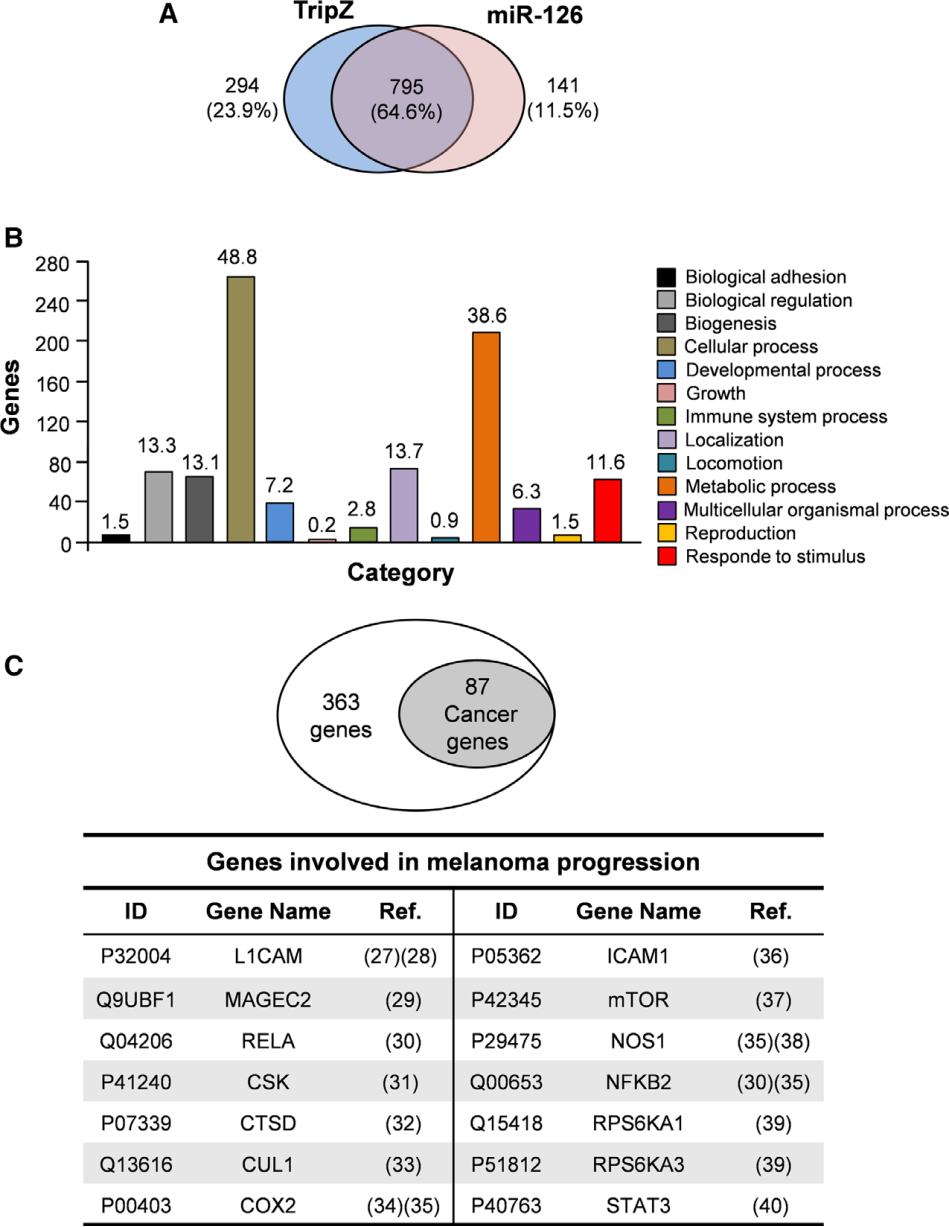
Proteome profiling of A375M/TripZ and A375M/miR‐126‐derived xenograft tumors. (A) Venn diagram showing common (795) and specific proteins identified in TripZ (294) and miR‐126 (141) xenograft tumors. (B) Bar histogram showing the results of a panther classification analysis by biological process of 363‐transcript dataset indicating the number of genes implicated in each process. The numbers above each bar represent the percentage of function hits. (C) Results obtained by david software analysis on 363‐transcript dataset indicated that 87 genes were involved in cancer pathways. Among these genes, we reported 14 genes involved in melanoma progression.

We then sought to elucidate the significance of miR‐126 restored expression on the metastatic melanoma cell proteome, identifying sensitive candidate pathways. To this purpose, we analyzed LC‐MS/MS, A375M/miR‐126‐ vs A375M/TripZ‐derived tumor proteome by reversed phase, with technical and biological reproducibility higher than 80% for both samples. We identified a total of 1230 proteins: 795 proteins were shared by both cell lines, and 294 were exclusive of TripZ and 141 of miR‐126 samples. Among those proteins common to TripZ and miR‐126 cells, 69 proteins were down‐regulated and 34 were up‐regulated by miR‐126 overexpression (Fig. 7A, Tables [Link], [Link]). Looking for the enrichment pathways possibly associated with the inhibitory action of miR‐126, we analyzed with panther software the annotations of 363 proteins, whose expression was restricted to A375M/TripZ or resulted in down‐regulation of miR‐126 cells, thus leading to a TripZ/miR‐126 ratio ≥ 2. The pattern of protein expression repressed or down‐regulated by miR‐126 overexpression was enriched in cellular and metabolic process clusters of genes (Fig. 7B).

Further analyses were then run using david software. Interestingly, as shown in Fig. 7C, the modulation by miR‐126 of several proteins involved in cancer and particularly in melanoma progression (Ernst *et al*., 2018; Laudisi *et al*., 2018) is consistent with tumor‐suppressing effects of this miR. Interestingly, the KEGG annotation analysis highlighted the mTOR signaling pathway, which represents one of the main signaling pathways activated in cancer through important metabolic changes (Mossmann *et al*., 2018). This result strengthens the tumor suppressor role of miR‐126 and its synergistic effect when associated with AKT and MAPK inhibitors (Fig. S11).

## 4 Discussion

An important challenge in clinical oncology is to overcome the acquired resistance often occurring after first‐line treatments, a field where malignant melanoma represents a paradigmatic pathology (Johannessen *et al*., 2013; Welsh *et al*., 2016). Vemurafenib treatment of patients with BRAF^V600^‐ mutated melanoma (≥ 50%) resulted in a significant prolongation of progression‐free survival (PFS), with an RR of 53%. Unfortunately, responses are not durable and most patients invariably relapse within 6–8 months with drug‐resistant disease (Savoia *et al*., 2019). A recent comparative analysis of survival data from selected trials representative for MAP kinase inhibition and immune checkpoint blockade (anti‐PD‐1 or CTLA‐4 monoclonal antibodies) showed the superiority of combined BRAF and MEK inhibitors in the first 6 months after initiation of treatment, whereas immune check point inhibitors were superior at 24 months. This observation, although preliminary, is consistent with the finding that as many as 60% of patients display primary resistance to immunotherapy but that a subpopulation of patients obtains a long‐term PFS. However, both therapeutic strategies unfortunately show a common phenomenon of acquired resistance and disease progression in a short period of time in a large proportion of patients (Najem *et al*., 2017; O'Donnell *et al*., 2016; Ugurel *et al*., 2017).

Our recent studies have identified miR‐126, already known to play an inhibitory role on the PI3K/AKT pathway (Xiao *et al*., 2016; Zhang *et al*., 2013), as a tumor‐suppressor in melanoma (Felli *et al*., 2013), demonstrating the existence of an auto‐regulatory loop that, connecting the tumor suppressor miR‐126 with the onco‐miR‐221/222, functions in melanoma development and progression (Felli *et al*., 2016). Also, significant modulations of miR have been associated with therapeutic treatments, indicating these short non‐coding RNA as additional factors for patient classification and clinical choices. Interestingly, miR‐126 was shown to be among those miR modulated by a combined treatment with temsirolimus (anti‐mTOR) and bevacizumab (anti‐VEGF) (Wagenseller *et al*., 2013).

Based on these data and seeking an effective therapeutic approach for metastatic melanoma, we demonstrated the potential role of miR‐126 as a sensitizing agent able to increase the efficacy of more traditional drugs. Combining drugs operating through different mechanisms of action is a therapeutic approach used to avoid the occurrence of resistance in oncology, but also against infectious agents easily developing drug resistance, such as *Mycobacterium tuberculosis* (Fischbach, 2011; Worthington and Melander, 2013). To find candidates for effective combinations, we screened a library of 349 anti‐cancer compounds, including several target agents already in clinical use or trials, looking for antiproliferative effects on the A375M metastatic melanoma cells. Among many others, we selected PIK‐75 for in‐depth evaluations in view of its high potency, but also for its inhibitory role on the p110α subunit of PI3K (Han and Zhang, 2010; Zheng *et al*., 2011), which in turn binds p85β, a second subunit directly targeted by miR‐126 (Xiao *et al*., 2016) (Fig. 1C). Indeed, this PIK‐75 selective inhibition could possibly induce less adverse side effects compared with pan–PI3K inhibitors (PI3Ki) (Ciraolo *et al*., 2011).

Actually, PIK‐75 is a potent inducer of apoptosis in primary human AML cells, enhancing the survival of mice with established AML without significant toxicity (Thomas *et al*., 2013). Besides PIK‐75, in combination therapy we utilized vemurafenib, currently used in patients with BRAF‐mutated advanced melanoma (Kim and Cohen, 2016). The treatment of metastatic melanoma cells with PIK‐75 or vemurafenib as single agents resulted in growth inhibition, but it was the combination of the two that produced a stronger synergistic effect, as indicated by statistical models (Fig. 2B). The molecular analysis after single‐agent treatment revealed a strong cross‐talk between PI3K/AKT and MAPK pathways, as shown by the inverse balance between pAKT and pERK levels (Fig. 3A,B). Indeed, we found the correct conditions for PIK‐75 and vemurafenib combined administration able to inhibit AKT phosphorylation, avoiding simultaneous MAPK activation. The main effect of this treatment was a strong induction of the apoptotic process. It is important to point out that the enforced expression of miR‐126 increased PIK‐75 activity in both single and combined treatments (Fig. 3C,D).

We then extended the evaluation of PIK‐75 supplementation to a vemurafenib‐resistant cell line (A375M‐VR), confirming its effectiveness in impairing cell viability through the induction of the apoptotic process, especially when associated with miR‐126 overexpression. PIK‐75 effectiveness was confirmed on two additional metastatic melanoma cell lines harboring the BRAF^V600E^ mutation, either in their standard conditions or selected for dabrafenib resistance (A375 vs A375‐R and SK‐Mel28 vs SK‐Mel28‐R). The same synergistic effects were obtained by treating four early passage cell lines obtained from metastatic melanoma patients (Pt1; Pt2; Pt3 and Pt4) with PIK‐75 and vemurafenib. Again, the analysis of the key molecular pathways confirmed that the combined treatment inhibits PI3K pathway and prevents MAPK reactivation (Fig. 5). Finally, the validity of this approach, which is able simultaneously to target PI3K and MAPK in the presence of miR‐126, was confirmed in a mouse xenogeneic model of human melanoma. In tumor samples, we showed reduced proliferation and increased apoptosis as indicated by Ki67 and caspase 3 modulations. According to recently published data (Hopkins *et al*., 2018), miR‐126 also appeared able to disrupt the insulin regulatory loop by reducing the expression of the key factor IRS‐1, reported as a target gene of miR‐126 (Fang *et al*., 2017). In addition, proteomic analysis showed the effectiveness of miR‐126 action on the mTOR pathway, one of the key controllers of cancer metabolism (Fig. 7). Overall, our results showed the capability of miR‐126 to enhance the efficacy of PI3K and/or MAPK inhibitors in line with what observed by reducing the systemic insulin response through a ketogenic diet (Hopkins *et al*., 2018). Since, at present, anti‐cancer drugs targeting IRS‐1 protein are not available (Reuveni *et al*., 2013), our data may represent preclinical proof of concept for miR‐126‐based direct targeting of IRS‐1, increasing the possible role of this miR in cancer therapy, including drug‐resistant melanoma.

There is growing evidence supporting the efficacy of combination therapies and the reduction of paradoxical activation of the MAPK pathway in melanoma (Lu *et al*., 2017; Nazarian *et al*., 2010; Tolcher *et al*., 2018). Nonetheless, one of the major problems is the severe toxicity associated with dual targeting therapeutics, particularly when based on a horizontal action, such as the use of MAPK and PI3K/AKT inhibitors (Shimizu *et al*., 2012). In this clinical setting, the restored expression of miR‐126 might have additional value. Indeed, novel more efficient and specific transfer technologies will favor miR‐126 administration.

## 5 Conclusions

Besides showing the higher efficacy of vemurafenib and PIK‐75 cotreatment, our results support the central role of miR‐126 in melanoma biology. Indeed, the restored expression of this miR in advanced melanoma could act as a sensitizer agent allowing reduced doses of the combined therapeutics, in turn leading to decreased frequency and severity of adverse events without limiting the benefits. In addition, miR‐126 direct targeting of the IRS‐1 may represent an efficient method to impair the insulin feedback loop reported to limit the efficacy of PI3K inhibition. Considering the excellent opportunity to use the PI3K/AKT inhibitors, miR‐126 might represent a novel promising tool for combination therapies.

## Conflict of interest

The authors declare no conflict of interest.

## Author contributions

FP and NF conceived the study, performed the experiments and analyzed data. FFe and MBA performed experiments. GDL and MS performed *in vivo* experiments. RP and GM performed immunofluorescence and immunohistochemical experiments. AB performed flow cytometry analysis and cell sorting. FFr performed proteomic and data processing. AG analyzed the data and performed the statistical analysis. SC provided dabrafenib‐resistant cell lines. AG and MB contributed to writing of the manuscript. NF supervised research activity and wrote the manuscript. AC wrote the manuscript. AC and MB found financial support.

## Supporting information


**Fig. S1**. qRT‐PCR analysis of miR‐126‐3p and miR‐126‐5p on A375M melanoma cell line.

**Fig. S2.** Dose‐response effects on A375M TripZ‐ and miR‐126‐transduced cells.

**Fig. S3**. Evaluation of p85β and p110δ direct targeting by miR‐126.

**Fig. S4**. Specific synergistic role of miR‐126‐3p in combination with vemurafenib and/or PIK‐75.

**Fig. S5.** Quantitation of western blot images reported in Fig. 3A–D.

**Fig. S6.** Synergistic effects of vemurafenib/PIK‐75/miR‐126 on PI3K/AKT and MAPK pathways in the A375M melanoma cell line.

**Fig. S7.** qRT‐PCR analysis of miR‐126‐3p and miR‐126‐5p on A375M‐VR melanoma cell line miR‐126‐ vs control‐transduced cells.

**Fig. S8.** Quantitation of western blot images reported in Fig. 4A–C.

**Fig. S9.** Quantitation of western blot images reported in Fig. 5D.

**Fig. S10.** Western blot quantitation and TUNEL and Ki67 positive cell count.

**Fig. S11.** PI3K‐AKT signaling pathway as shown by KEGG analysis.

**Table S1.** List of the 349 compounds of the Selleckchem anti‐cancer library, including names, targets and a brief description of the drugs.

**Table S2.** Cell viability at 24 and 48 h of treatment with 349 anti‐cancer compounds.

**Table S3**. Proteome profiling of A375M/TripZ‐ and A375M/miR‐126‐derived xenograft tumors: complete list of the identified proteins.

**Table S4.** Quantitative profile of differentially expressed proteins by comparing TripZ and miR‐126.

## References

[mol212506-bib-0001] Alhasan L (2019) MiR‐126 modulates angiogenesis in breast cancer by targeting VEGF‐A–mRNA. Asian Pac J Cancer Prev 20, 193–197. 30678431 10.31557/APJCP.2019.20.1.193 PMC6485552

[mol212506-bib-0002] Apalla Z , Lallas A , Sotiriou E , Lazaridou E and Ioannides D (2017) Epidemiological trends in skin cancer. Dermatol Pract Concep 7, 1–6. 10.5826/dpc.0702a01 PMC5424654 28515985

[mol212506-bib-0003] Boussadia Z , Lamberti J , Mattei F , Pizzi E , Puglisi R , Zanetti C , Pasquini L , Fratini F , Fantozzi L , Felicetti F *et al* (2018) Acidic microenvironment plays a key role in human melanoma progression through a sustained exosome mediated transfer of clinically relevant metastatic molecules. J Exp Clinl Cancer Res 37, 245. 10.1186/s13046-018-0915-z PMC6173926 30290833

[mol212506-bib-0004] Cantwell‐Dorris ER , O'Leary JJ and Sheils OM (2011) BRAFV600E: implications for carcinogenesis and molecular therapy. Mol Cancer Ther 10, 385–394. 21388974 10.1158/1535-7163.MCT-10-0799

[mol212506-bib-0005] Caporali S , Alvino E , Lacal PM , Levati L , Giurato G , Memoli D , Caprini E , Antonini Cappellini GC and D'Atri S (2016) Targeting the PI3K/AKT/mTOR pathway overcomes the stimulating effect of dabrafenib on the invasive behavior of melanoma cells with acquired resistance to the BRAF inhibitor. Int J Oncol 49, 1164–1174. 27572607 10.3892/ijo.2016.3594

[mol212506-bib-0006] Caporali S , Alvino E , Lacal PM , Ruffini F , Levati L , Bonmassar L , Scoppola A , Marchetti P , Mastroeni S , Antonini Cappellini GC *et al* (2017) Targeting the PTTG1 oncogene impairs proliferation and invasiveness of melanoma cells sensitive or with acquired resistance to the BRAF inhibitor dabrafenib. Oncotarget 8, 113472–113493. 29371923 10.18632/oncotarget.23052 PMC5768340

[mol212506-bib-0007] Chan XY , Singh A , Osman N and Piva TJ (2017) Role played by signalling pathways in overcoming BRAF inhibitor resistance in melanoma. Int J Mol Sci 18. 10.3390/ijms18071527 PMC5536016 28708099

[mol212506-bib-0008] Ciraolo E , Morello F and Hirsch E (2011) Present and future of PI3K pathway inhibition in cancer: perspectives and limitations. Curr Med Chem 18, 2674–2685. 21649577 10.2174/092986711796011193

[mol212506-bib-0009] Davies H , Bignell GR , Cox C , Stephens P , Edkins S , Clegg S , Teague J , Woffendin H , Garnett MJ , Bottomley W *et al* (2002) Mutations of the BRAF gene in human cancer. Nature 417, 949–954. 12068308 10.1038/nature00766

[mol212506-bib-0010] Eggermont AM , Spatz A and Robert C (2014) Cutaneous melanoma. Lancet 383, 816–827. 24054424 10.1016/S0140-6736(13)60802-8

[mol212506-bib-0011] Ernst AK , Putscher A , Samatov TR , Suling A , Galatenko VV , Shkurnikov MY , Knyazev EN , Tonevitsky AG , Haalck T , Lange T *et al* (2018) Knockdown of L1CAM significantly reduces metastasis in a xenograft model of human melanoma: L1CAM is a potential target for anti‐melanoma therapy. PLoS ONE 13, e0192525. 29432466 10.1371/journal.pone.0192525 PMC5809060

[mol212506-bib-0012] Fang S , Ma X , Guo S and Lu J (2017) MicroRNA‐126 inhibits cell viability and invasion in a diabetic retinopathy model via targeting IRS‐1. Oncol Lett 14, 4311–4318. 28943945 10.3892/ol.2017.6695 PMC5604121

[mol212506-bib-0013] Felli N , Felicetti F , Lustri AM , Errico MC , Bottero L , Cannistraci A , De Feo A , Petrini M , Pedini F , Biffoni M *et al* (2013) miR‐126&126* restored expressions play a tumor suppressor role by directly regulating ADAM9 and MMP7 in melanoma. PLoS ONE 8, e56824. 23437250 10.1371/journal.pone.0056824 PMC3578857

[mol212506-bib-0014] Felli N , Errico MC , Pedini F , Petrini M , Puglisi R , Bellenghi M , Boe A , Felicetti F , Mattia G , De Feo A *et al* (2016) AP2alpha controls the dynamic balance between miR‐126&126* and miR‐221&222 during melanoma progression. Oncogene 35, 3016–3026. 26434590 10.1038/onc.2015.357 PMC4908437

[mol212506-bib-0015] Feng R , Beeharry MK , Lu S , Sah BK , Yuan F , Yan M , Liu B , Li C and Zhu Z (2018) Down‐regulated serum miR‐126 is associated with aggressive progression and poor prognosis of gastric cancer. Cancer Biomark 119–126. 29562500 10.3233/CBM-171099 PMC13078457

[mol212506-bib-0016] Fiala O , Pitule P , Hosek P , Liska V , Sorejs O , Bruha J , Vycital O , Buchler T , Poprach A , Topolcan O *et al* (2017) The association of miR‐126‐3p, miR‐126‐5p and miR‐664‐3p expression profiles with outcomes of patients with metastatic colorectal cancer treated with bevacizumab. Tumour Biol 39 10.1177/1010428317709283 28714375

[mol212506-bib-0017] Fischbach MA (2011) Combination therapies for combating antimicrobial resistance. Curr Opin Microbiol 14, 519–523. 21900036 10.1016/j.mib.2011.08.003 PMC3196371

[mol212506-bib-0018] Fratini F , Raggi C , Sferra G , Birago C , Sansone A , Grass F , Curra C , Olivieri A , Pace T , Mochi S *et al* (2017) An integrated approach to explore composition and dynamics of cholesterol‐rich membrane microdomains in sexual stages of malaria parasite. Mol Cell Proteomics 16, 1801–1814. 28798222 10.1074/mcp.M117.067041 PMC5629265

[mol212506-bib-0019] George DD , Armenio VA and Katz SC (2017) Combinatorial immunotherapy for melanoma. Cancer Gene Ther 24, 141–147. 27834353 10.1038/cgt.2016.56

[mol212506-bib-0020] Han M and Zhang JZ (2010) Class I phospho‐inositide‐3‐kinases (PI3Ks) isoform‐specific inhibition study by the combination of docking and molecular dynamics simulation. J Chem Inf Model 50, 136–145. 19928754 10.1021/ci900175n

[mol212506-bib-0021] Han L , Liu H , Wu J and Liu J (2018) miR‐126 suppresses invasion and migration of malignant glioma by targeting mature T cell proliferation 1 (MTCP1). Med Sci Monit 20, 6630–6637. 10.12659/MSM.910292 PMC6161564 30233082

[mol212506-bib-0022] Hepner A , Salgues A , Anjos CAD , Sahade M , Camargo VP , Garicochea B , Shoushtari AN , Postow MA , Fernandes GS and Munhoz RR (2017) Treatment of advanced melanoma – A changing landscape. Rev Assoc Med Bras 63, 814–823. 29239458 10.1590/1806-9282.63.09.814

[mol212506-bib-0023] Hopkins BD , Pauli C , Du X , Wang DG , Li X , Wu D , Amadiume SC , Goncalves MD , Hodakoski C , Lundquist MR *et al* (2018) Suppression of insulin feedback enhances the efficacy of PI3K inhibitors. Nature 560, 499–503. 30051890 10.1038/s41586-018-0343-4 PMC6197057

[mol212506-bib-0024] Huang T , Wang X , Yang X , Ji J , Wang Q , Yue X and Dong Z (2018) Long non‐coding RNA DUXAP8 enhances renal cell carcinoma progression via downregulating miR‐126. Med Sci Monit 24, 7340–7347. 30317248 10.12659/MSM.910054 PMC6198709

[mol212506-bib-0025] Johannessen CM , Johnson LA , Piccioni F , Townes A , Frederick DT , Donahue MK , Narayan R , Flaherty KT , Wargo JA , Root DE *et al* (2013) A melanocyte lineage program confers resistance to MAP kinase pathway inhibition. Nature 504, 138–142. 24185007 10.1038/nature12688 PMC4098832

[mol212506-bib-0026] Kim A and Cohen MS (2016) The discovery of vemurafenib for the treatment of BRAF‐mutated metastatic melanoma. Expert Opin Drug Discov 11, 907–916. 27327499 10.1080/17460441.2016.1201057 PMC5443413

[mol212506-bib-0027] Laudisi F , Cherubini F , Monteleone G and Stolfi C (2018) STAT3 interactors as potential therapeutic targets for cancer treatment. Int J Mol Sci 19 10.3390/ijms19061787 PMC6032216 29914167

[mol212506-bib-0028] Lim SY , Menzies AM and Rizos H (2017) Mechanisms and strategies to overcome resistance to molecularly targeted therapy for melanoma. Cancer 123, 2118–2129. 28543695 10.1002/cncr.30435

[mol212506-bib-0029] Liu R , Zhang Y‐S , Zhang S , Cheng Z‐M , Yu J‐L , Zhou J and Song J (2019) MiR‐126‐3p suppresses the growth, migration and invasion of NSCLC via targeting CCR29. Eur Rev Med Pharmacol Sci 23, 679–689. 30720175 10.26355/eurrev_201901_16881

[mol212506-bib-0030] Lu H , Liu S , Zhang G , Bin W , Zhu Y , Frederick DT , Hu Y , Zhong W , Randell S , Sadek N *et al* (2017) PAK signalling drives acquired drug resistance to MAPK inhibitors in BRAF‐mutant melanomas. Nature 550, 133–136. 28953887 10.1038/nature24040 PMC5891348

[mol212506-bib-0031] Massacesi C , Di Tomaso E , Urban P , Germa C , Quadt C , Trandafir L , Aimone P , Fretault N , Dharan B , Tavorath R *et al* (2016) PI3K inhibitors as new cancer therapeutics: implications for clinical trial design. Onco Targets Ther 9, 203–210. 26793003 10.2147/OTT.S89967 PMC4708174

[mol212506-bib-0032] Matthews NH , Li WQ , Quresh AA , Weinstock MA and Cho E (2017) Epidemiology of melanoma In Cutaneous Melanoma: Etiology and Therapy (Ward WH and Farma JM, eds), pp. 3–22. Codon Publications, Brisbane. 29461782

[mol212506-bib-0033] Mossmann D , Park S and Hall MN (2018) mTOR signalling and cellular metabolism are mutual determinants in cancer. Nat Rev Cancer 18, 744–757. 30425336 10.1038/s41568-018-0074-8

[mol212506-bib-0034] Najem A , Krayem M , Perdrix A , Kerger J , Awada A , Journe F and Ghanem G (2017) New drug combination strategies in melanoma: current status and future directions. Anticancer Res 37, 5941–5953. 29061773 10.21873/anticanres.12041

[mol212506-bib-0035] Nazarian R , Shi H , Wang Q , Kong X , Koya RC , Lee H , Chen Z , Lee MK , Attar N , Sazegar H *et al* (2010) Melanomas acquire resistance to B‐RAF(V600E) inhibition by RTK or N‐RAS upregulation. Nature 468, 973–977. 21107323 10.1038/nature09626 PMC3143360

[mol212506-bib-0036] O'Donnell JS , Smyth MJ and Teng MW (2016) Acquired resistance to anti‐PD1 therapy: checkmate to checkpoint blockade? Genome Med 8, 111. 27782862 10.1186/s13073-016-0365-1 PMC5080691

[mol212506-bib-0037] Pappalardo F , Russo G , Candido S , Pennisi M , Cavalieri S , Motta S , McCubrey JA , Nicoletti F and Libra M (2016) Computational modeling of PI3K/AKT and MAPK signaling pathways in melanoma cancer. PLoS ONE 11, e0152104. 27015094 10.1371/journal.pone.0152104 PMC4807832

[mol212506-bib-0038] Reuveni H , Flashner‐Abramson E , Steiner L , Makedonski K , Song R , Shir A , Herlyn M , Bar‐Eli M and Levitzki A (2013) Therapeutic destruction of insulin receptor substrates for cancer treatment. Cancer Res 73, 4383–4394. 23651636 10.1158/0008-5472.CAN-12-3385 PMC4391644

[mol212506-bib-0039] Savoia P , Fava P , Casoni F and Cremona O (2019) Targeting the ERK signaling pathway in melanoma. Int J Mol Sci 20, 1483. 10.3390/ijms20061483 PMC6472057 30934534

[mol212506-bib-0040] Shimizu T , Tolcher AW , Papadopoulos KP , Beeram M , Rasco DW , Smith LS , Gunn S , Smetzer L , Mays TA , Kaiser B *et al* (2012) The clinical effect of the dual‐targeting strategy involving PI3K/AKT/mTOR and RAS/MEK/ERK pathways in patients with advanced cancer. Clin Cancer Res 18, 2316–2325. 22261800 10.1158/1078-0432.CCR-11-2381

[mol212506-bib-0041] Shtivelman E , Davies MQ , Hwu P , Yang J , Lotem M , Oren M , Flaherty KT and Fisher DE (2014) Pathways and therapeutic targets in melanoma. Oncotarget 5, 1701–1752. 24743024 10.18632/oncotarget.1892 PMC4039128

[mol212506-bib-0042] Smalley KS and Sondak VK (2013) Targeted therapy for melanoma: is double hitting a home run? Nat Rev Clin Oncol 10, 5–6. 10.1038/nrclinonc.2012.215 23207793

[mol212506-bib-0043] Su F , Bradley WD , Wang Q , Yang H , Xu L , Higgins B , Kolinsky K , Packman K , Kim MJ , Trunzer K *et al* (2012) Resistance to selective BRAF inhibition can be mediated by modest upstream pathway activation. Cancer Res 72, 969–978. 22205714 10.1158/0008-5472.CAN-11-1875

[mol212506-bib-0044] Sun Z , Ou C , Liu J , Chen C , Zhou Q , Yang S , Li G , Wang G , Song J , Li Z *et al* (2019) YAP1‐induced MALAT1 promotes epithelial‐mesenchymal transition and angiogenesis by sponging miR‐126‐5p in colorectal cancer. Oncogene 38, 2627–2644. 30531836 10.1038/s41388-018-0628-y PMC6484768

[mol212506-bib-0045] Thomas D , Powell JA , Vergez F , Segal DH , Nguyen NY , Baker A , Teh TC , Barry EF , Sarry JE , Lee EM *et al* (2013) Targeting acute myeloid leukemia by dual inhibition of PI3K signaling and Cdk9‐mediated Mcl‐1 transcription. Blood 122, 738–748. 23775716 10.1182/blood-2012-08-447441

[mol212506-bib-0046] Tolcher AW , Peng W and Calvo E (2018) Rational approaches for combination therapy strategies targeting the MAP kinase pathway in solid tumors. Mol Cancer Ther 17, 3–16. 29295962 10.1158/1535-7163.MCT-17-0349

[mol212506-bib-0047] Ugurel S , Rohmel J , Ascierto PA , Flaherty KT , Grob JJ , Hauschild A , Larkin J , Long GV , Lorigan P , McArthur GA *et al* (2017) Survival of patients with advanced metastatic melanoma: the impact of novel therapies‐update 2017. Eur J Cancer 83, 247–257. 28756137 10.1016/j.ejca.2017.06.028

[mol212506-bib-0048] Volpe VO , Klufas DM , Hegde U and Grant‐Kels JM (2017) The new paradigm of systemic therapies for metastatic melanoma. J Am Acad Dermatol 77, 356–368. 28711086 10.1016/j.jaad.2017.04.1126

[mol212506-bib-0049] Wagenseller AG , Shada A , D'Auria KM , Murphy C , Sun D , Molhoek KR , Papin JA , Dutta A and Slingluff CL Jr (2013) MicroRNAs induced in melanoma treated with combination targeted therapy of Temsirolimus and Bevacizumab. J Trans Med 11, 218. 10.1186/1479-5876-11-218 PMC3853033 24047116

[mol212506-bib-0050] Wang J , Ding M , Zhu H , Cao Y and Zhao W (2019) Up‐regulation of long noncoding RNA MINCR promotes non‐small cell of lung cancer growth by negatively regulating miR‐126/SLC7A5 axis. Biochem Biophys Res Commun 15, 80–784. 10.1016/j.bbrc.2018.11.162 30528230

[mol212506-bib-0051] Welsh SJ , Rizos H , Scolyer RA and Long GV (2016) Resistance to combination BRAF and MEK inhibition in metastatic melanoma: where to next? Eur J Cancer 62, 76–85. 27232329 10.1016/j.ejca.2016.04.005

[mol212506-bib-0052] Worthington RJ and Melander C (2013) Combination approaches to combat multidrug‐resistant bacteria. Trends Biotechnol 31, 177–184. 23333434 10.1016/j.tibtech.2012.12.006 PMC3594660

[mol212506-bib-0053] Xiao J , Lin HY , Zhu YY , Zhu YP and Chen LW (2016) MiR‐126 regulates proliferation and invasion in the bladder cancer BLS cell line by targeting the PIK3R2‐mediated PI3K/Akt signaling pathway. Onco Targets Ther 9, 5181–5193. 27578985 10.2147/OTT.S105198 PMC5001672

[mol212506-bib-0054] Zhang J , Zhang Z , Zhang DY , Zhu J , Zhang T and Wang C (2013) microRNA 126 inhibits the transition of endothelial progenitor cells to mesenchymal cells via the PIK3R2‐PI3K/Akt signalling pathway. PLoS ONE 8, e83294. 24349482 10.1371/journal.pone.0083294 PMC3862723

[mol212506-bib-0055] Zhang W , Zhou J , Zhu X and Yuan H (2017) MiR‐126 reverses drug resistance to TRAIL through inhibiting the expression of c‐FLIP in cervical cancer. Gene 627, 420–427. 28669929 10.1016/j.gene.2017.06.055

[mol212506-bib-0056] Zheng Z , Amran SI , Thompson PE and Jennings IG (2011) Isoform‐selective inhibition of phosphoinositide 3‐kinase: identification of a new region of nonconserved amino acids critical for p110alpha inhibition. Mol Pharm 80, 657–664. 10.1124/mol.111.072546 21778304

