# Supplementary figures and images for: Joint action of miR‐126 and MAPK/PI3K inhibitors against metastatic melanoma
Source: Mol Oncol. 2019 Aug 6;13(9):1836–54. doi: 10.1002/1878-0261.12506 (PMC6717748; doi:10.1002/1878-0261.12506)

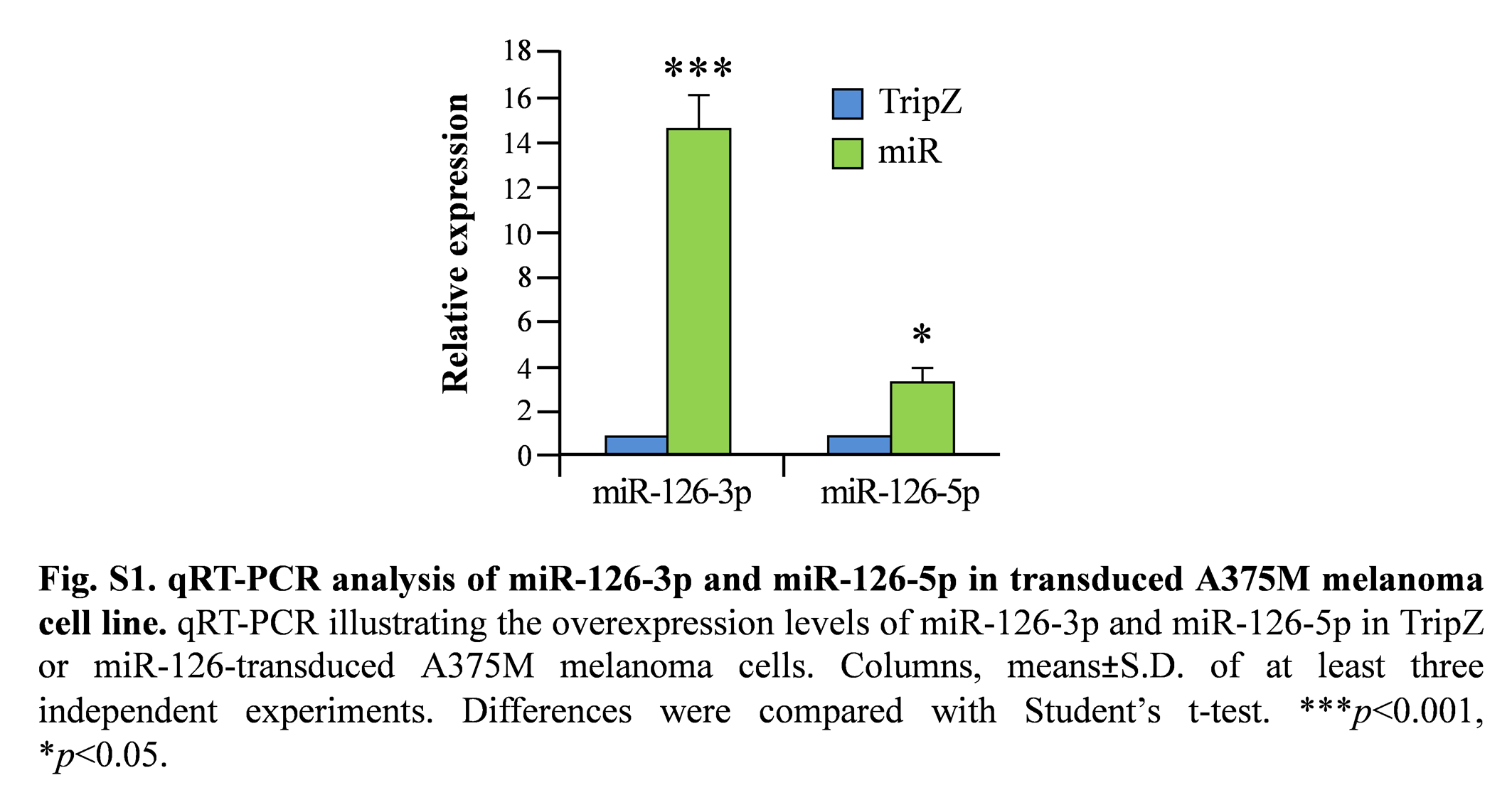

Supplement: Supplementary file 1 — Fig. S1. qRT‐PCR analysis of miR‐126‐3p and miR‐126‐5p on A375M melanoma cell line. [file MOL2-13-1836-s001.tif]

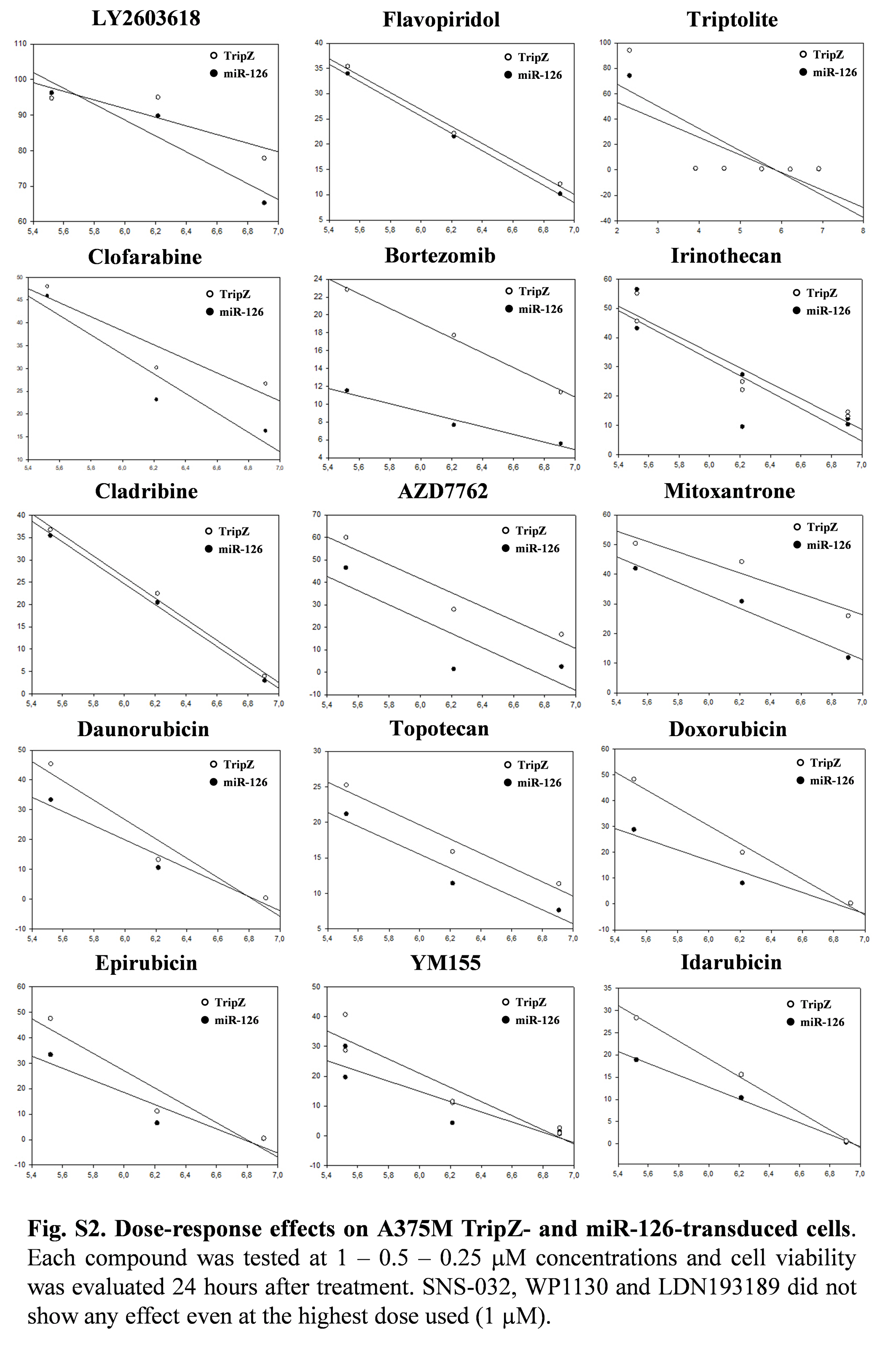

Supplement: Supplementary file 2 — Fig. S2. Dose‐response effects on A375M TripZ‐ and miR‐126‐transduced cells. [file MOL2-13-1836-s002.tif]

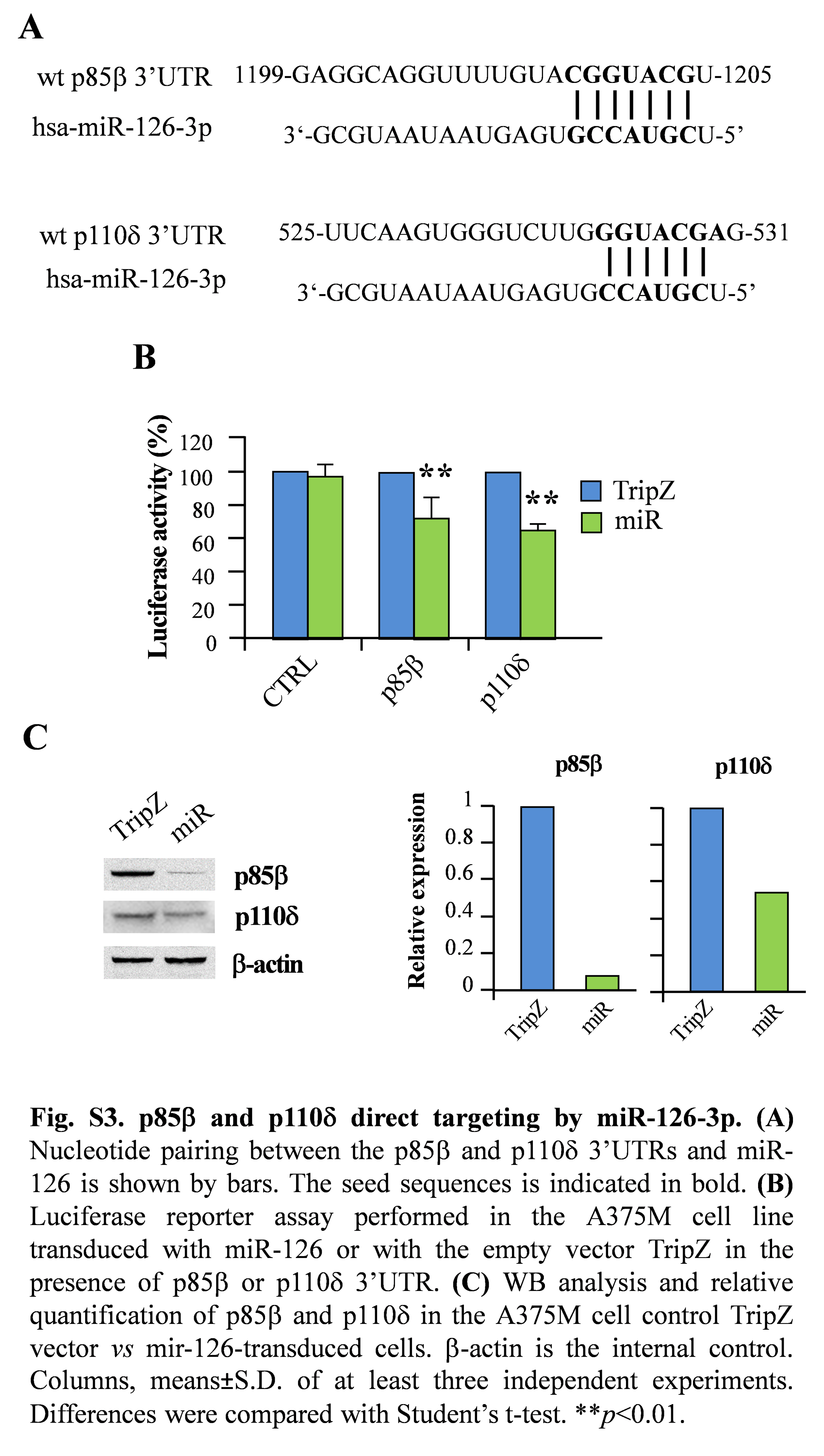

Supplement: Supplementary file 3 — Fig. S3. Evaluation of p85β and p110δ direct targeting by miR‐126. [file MOL2-13-1836-s003.tif]

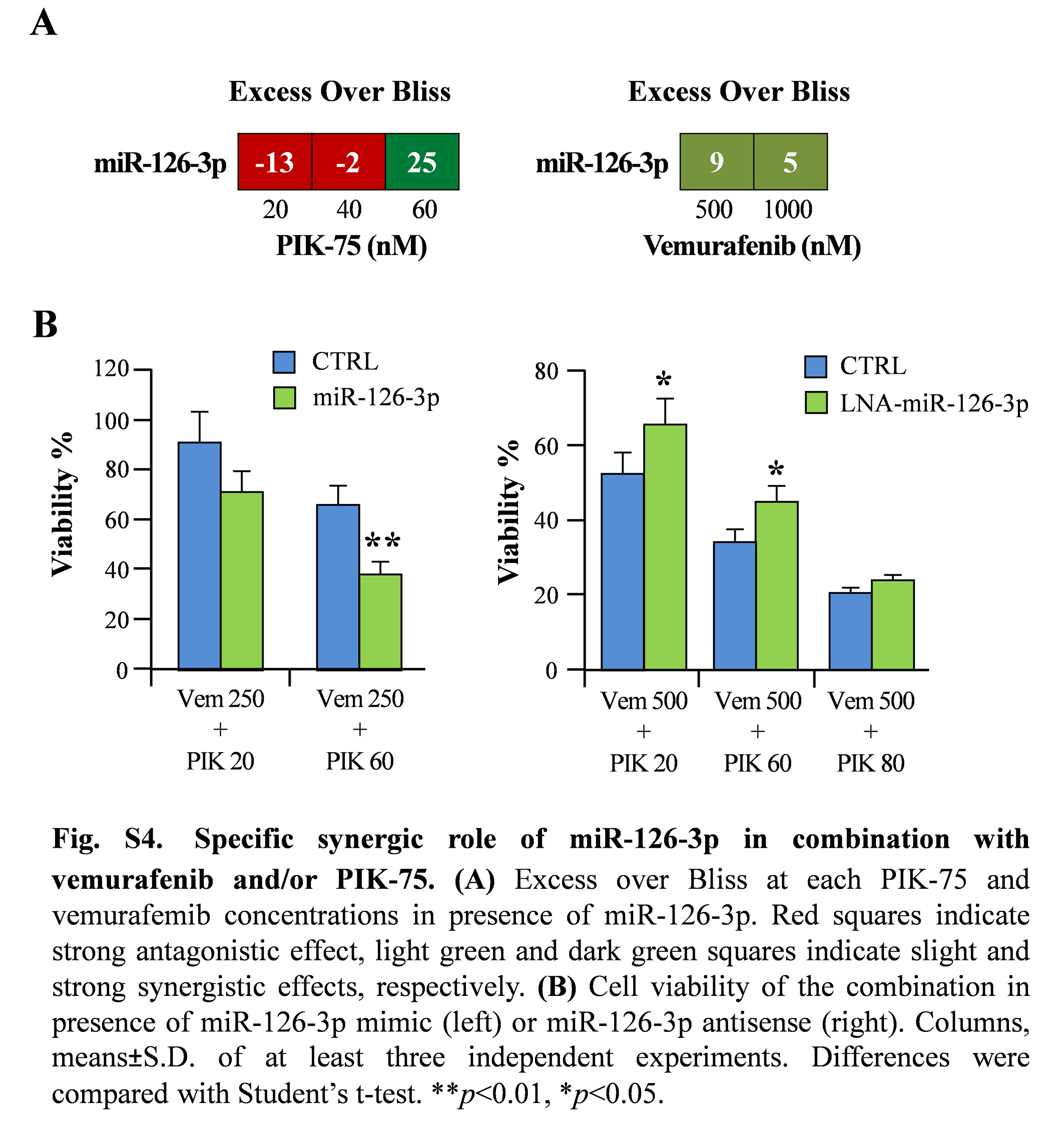

Supplement: Supplementary file 4 — Fig. S4. Specific synergistic role of miR‐126‐3p in combination with vemurafenib and/or PIK‐75. [file MOL2-13-1836-s004.tif]

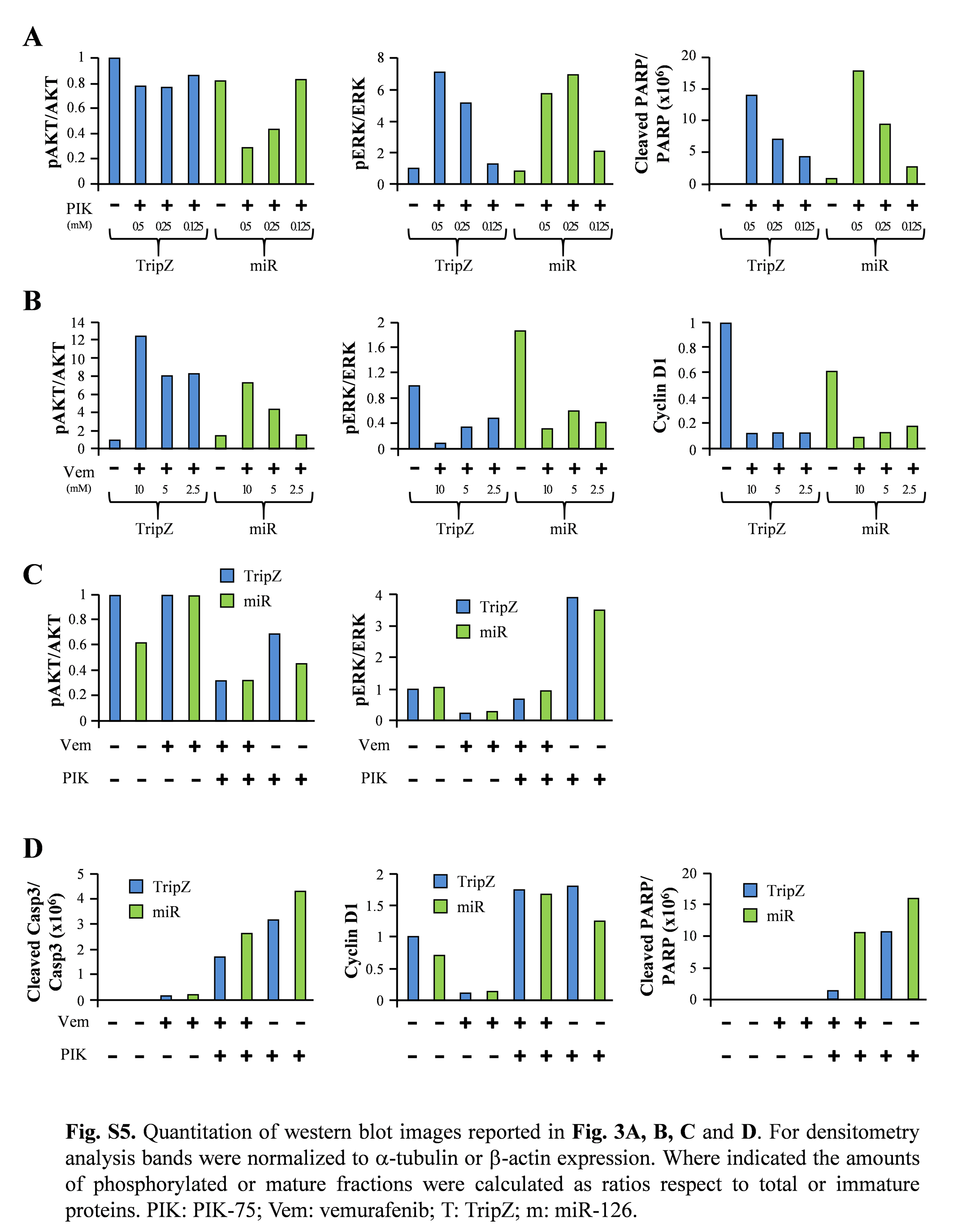

Supplement: Supplementary file 5 — Fig. S5. Quantitation of western blot images reported in Fig. 3A–D. [file MOL2-13-1836-s005.tif]

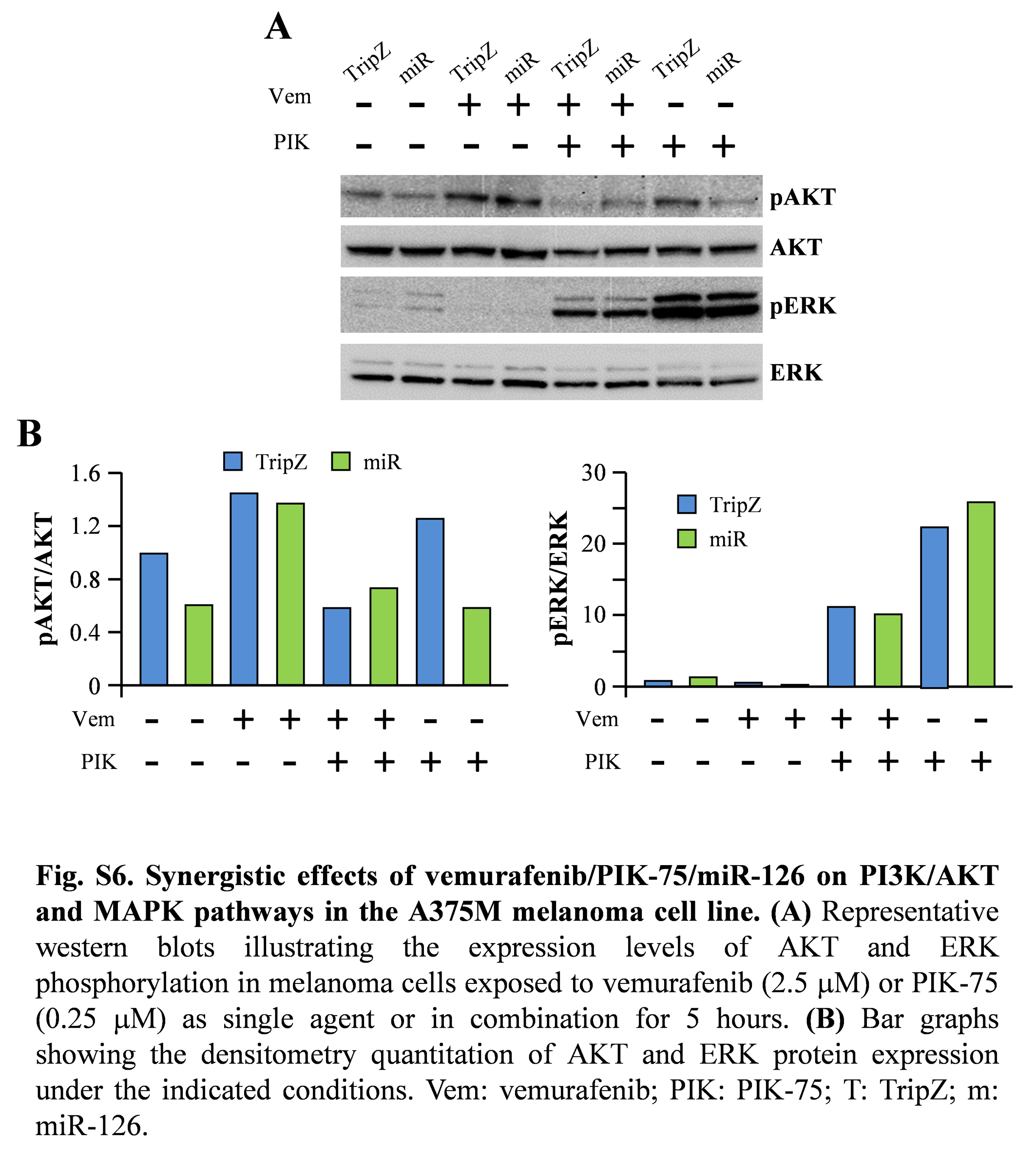

Supplement: Supplementary file 6 — Fig. S6. Synergistic effects of vemurafenib/PIK‐75/miR‐126 on PI3K/AKT and MAPK pathways in the A375M melanoma cell line. [file MOL2-13-1836-s006.tif]

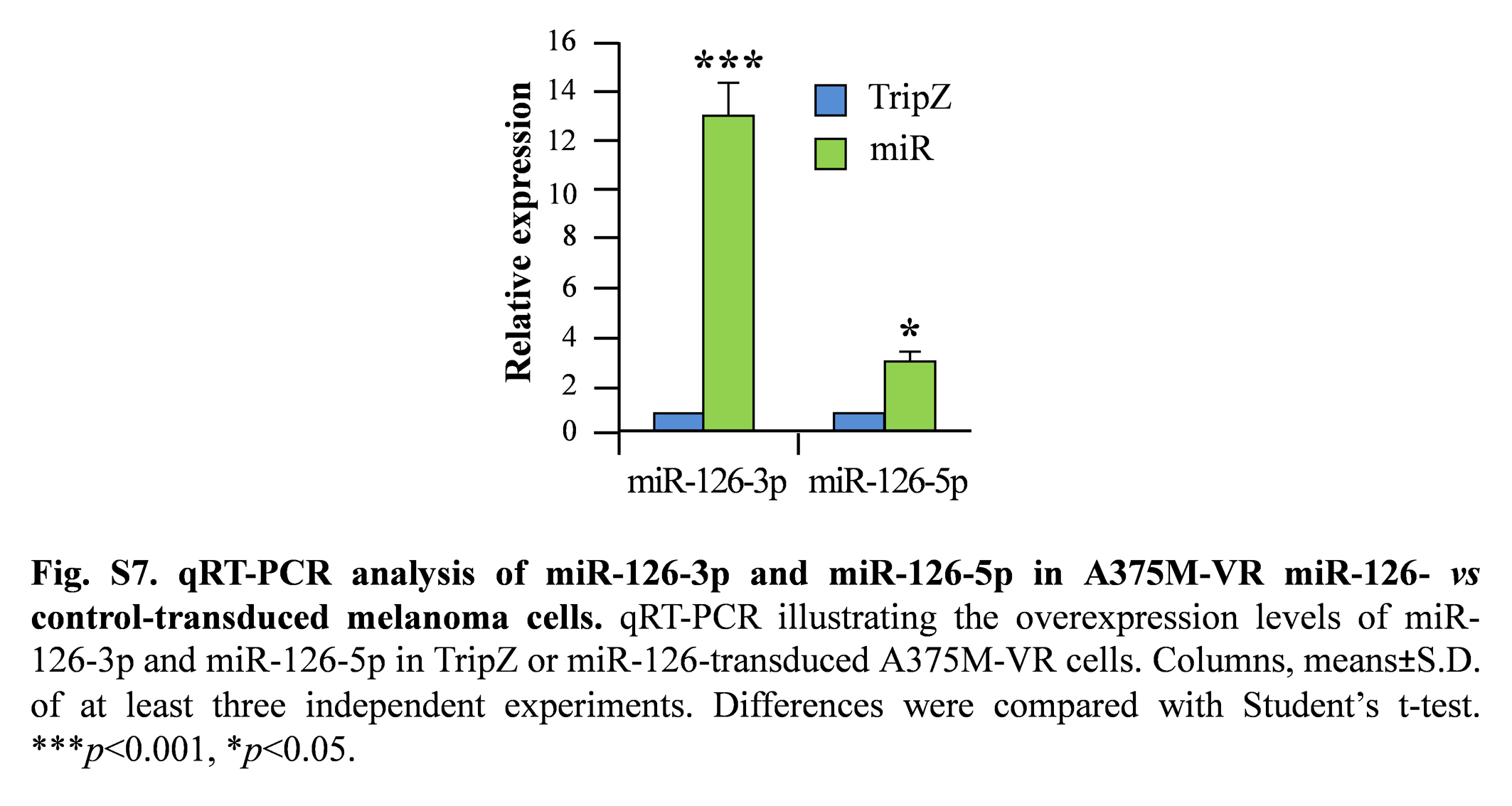

Supplement: Supplementary file 7 — Fig. S7. qRT‐PCR analysis of miR‐126‐3p and miR‐126‐5p on A375M‐VR melanoma cell line miR‐126‐ vs control‐transduced cells. [file MOL2-13-1836-s007.tif]

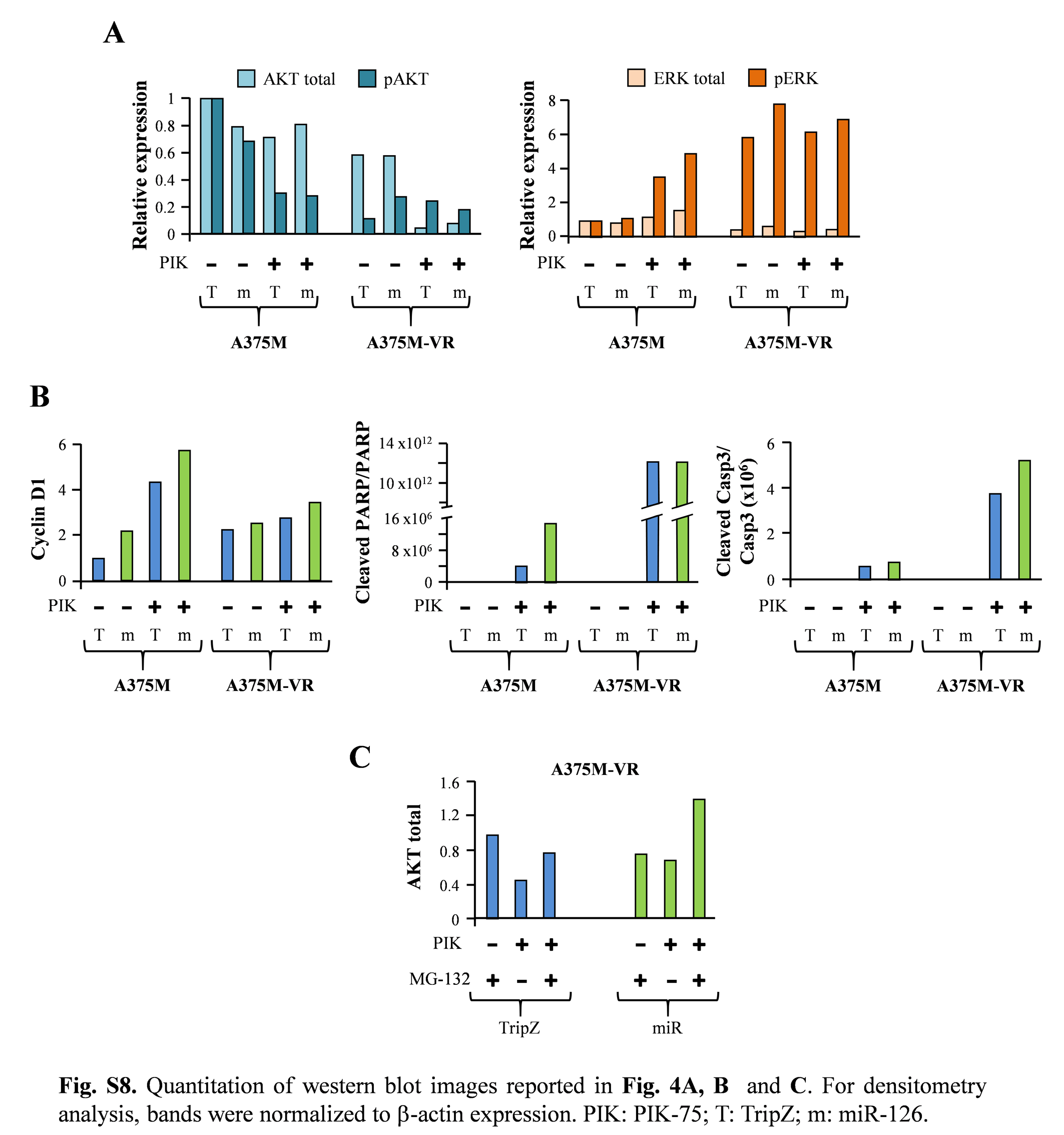

Supplement: Supplementary file 8 — Fig. S8. Quantitation of western blot images reported in Fig. 4A–C. [file MOL2-13-1836-s008.tif]

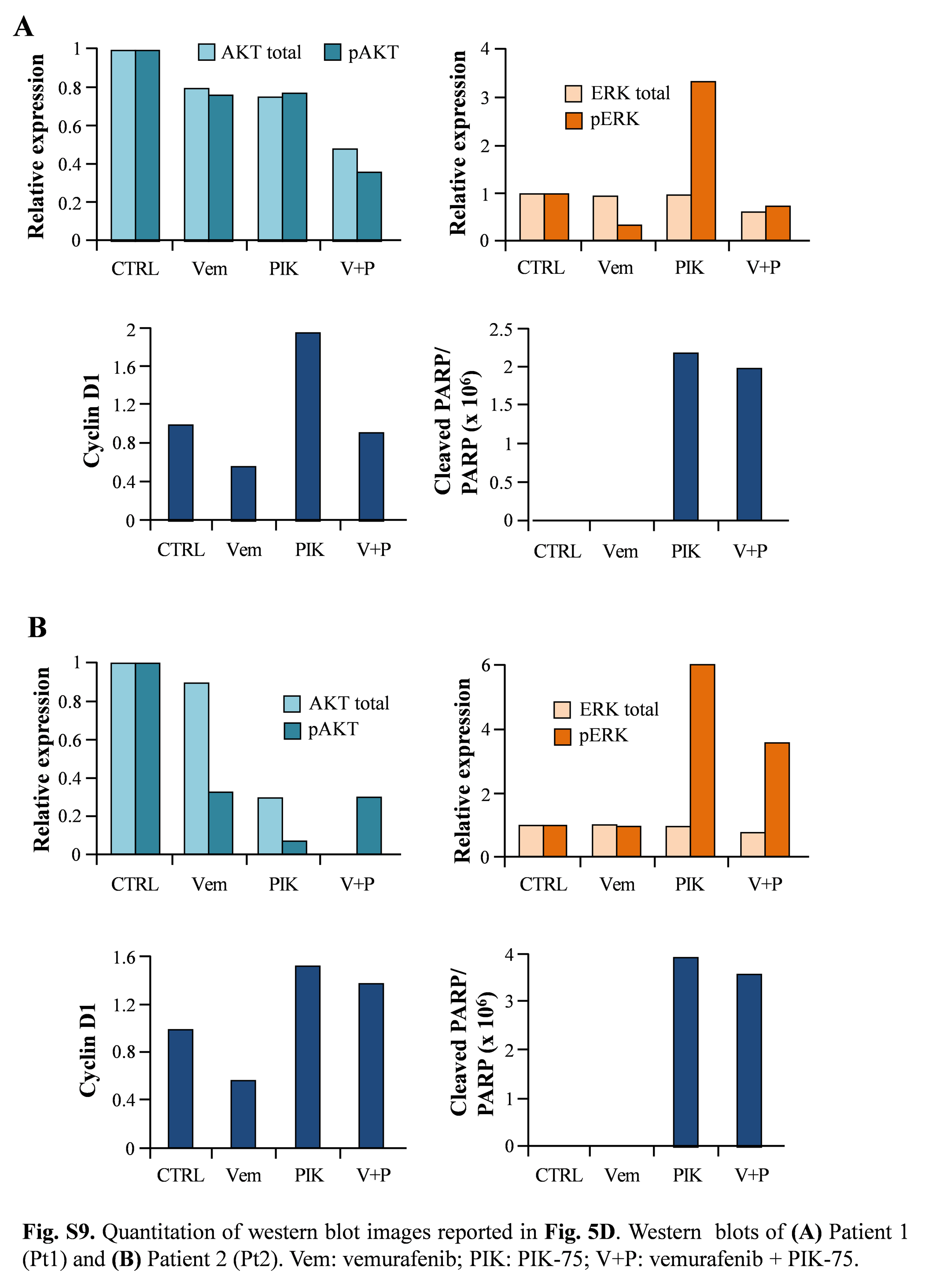

Supplement: Supplementary file 9 — Fig. S9. Quantitation of western blot images reported in Fig. 5D. [file MOL2-13-1836-s009.tif]

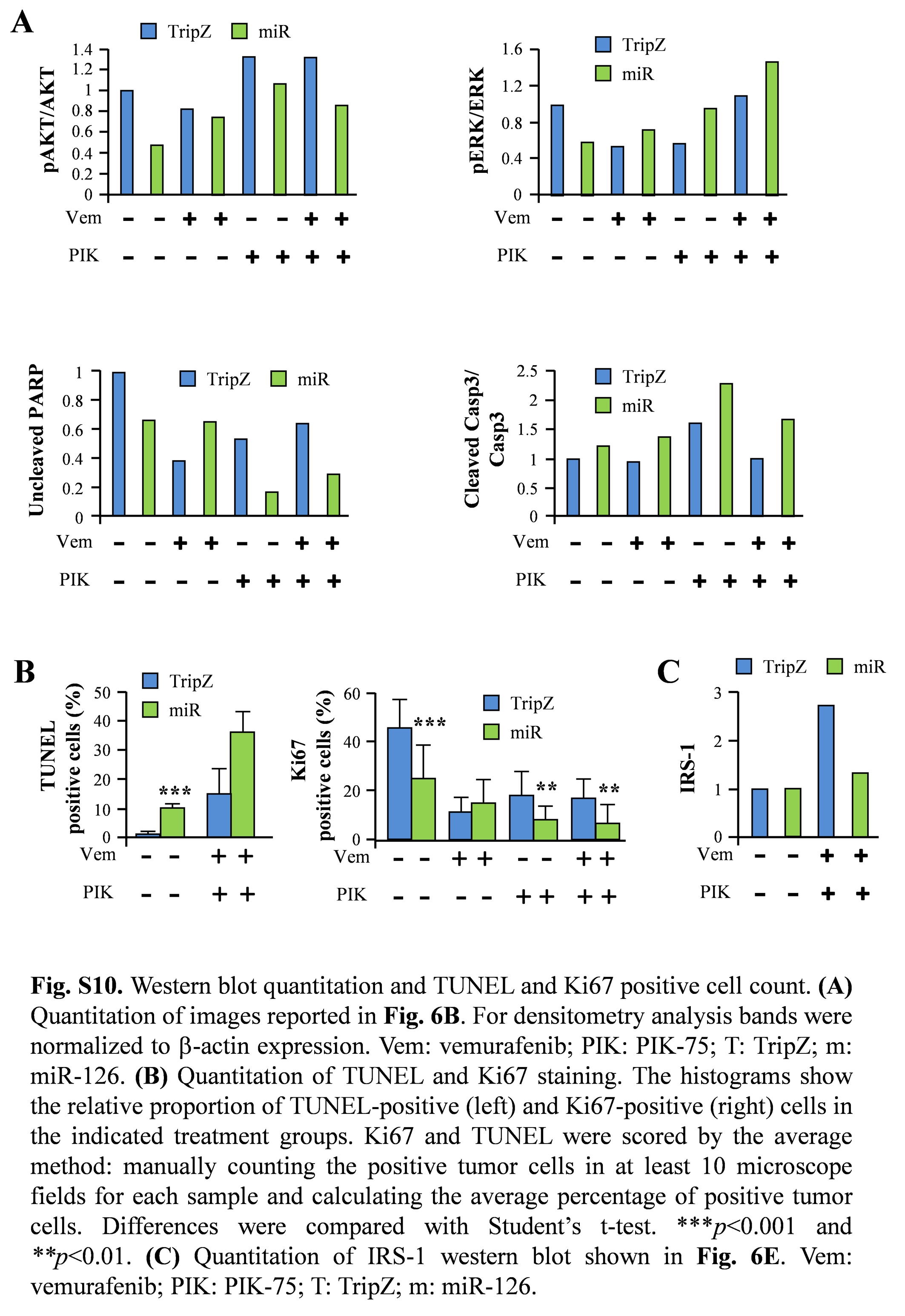

Supplement: Supplementary file 10 — Fig. S10. Western blot quantitation and TUNEL and Ki67 positive cell count. [file MOL2-13-1836-s010.tif]

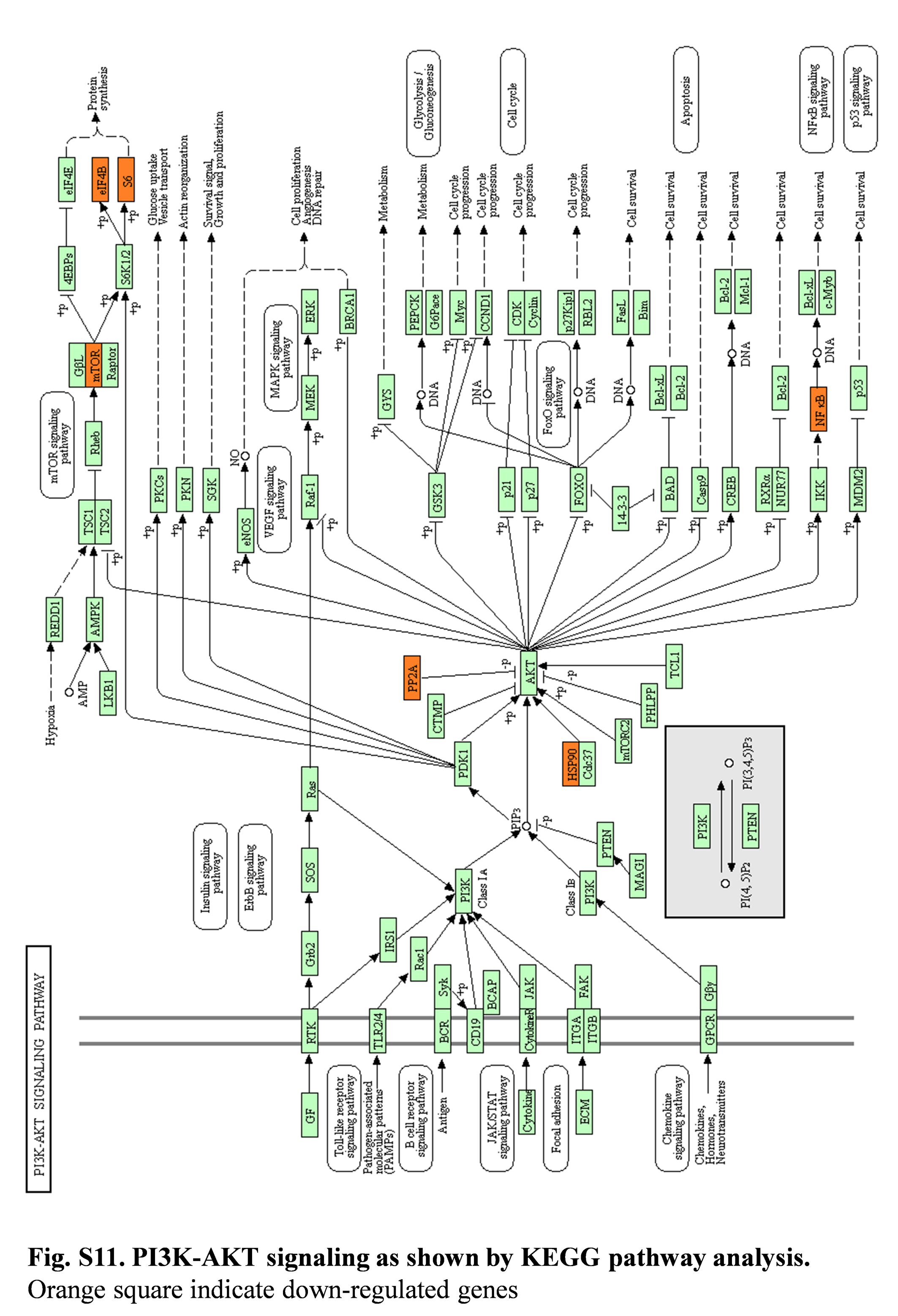

Supplement: Supplementary file 11 — Fig. S11. PI3K‐AKT signaling pathway as shown by KEGG analysis. [file MOL2-13-1836-s011.tif]
