# Supplementary material for: Joint action of miR‐126 and MAPK/PI3K inhibitors against metastatic melanoma
Source: Mol Oncol. 2019 Aug 6;13(9):1836–54. doi: 10.1002/1878-0261.12506 (PMC6717748; doi:10.1002/1878-0261.12506)
Supplement: Supplementary file 12 — Table S1. List of the 349 compounds of the Selleckchem anti‐cancer library, including names, targets and a brief description of the drugs. [file MOL2-13-1836-s012.pdf]

Supplementary Table S1. List of the 349 compounds of the Selleck Anti-cancer Library, including names, targets and a brief description of the drugs.

COMPOUND	TARGET	DESCRIPTION
**(-)-Epigallocatechin gallate**	Telomerase	A potent anti-oxidant polyphenol flavonoid that inhibits telomerase and DNA methyltransferase. EGCG blocks the activation of EGFRs and HER-2Rs
**17-AAG (Tanespimycin)**	HSP (e.g. HSP90)	An analog of geldanamycin and is a potent HSP90 inhibitor
**17-DMAG HCl (Alvespimycin)**	HSP (e.g. HSP90)	A potent HSP90 inhibitor
**2-Methoxyestradiol**	HIF	A tubulin polymerization inhibitor and also blocks HIF-1a nuclear accumulation and HIF-trascriptional activity.
**3-Methyladenine**	Autophagy	A selective PI3K inhibitor for Vps34 and PI3Kγ and also blocks autophagosome formation.
**A-769662**	AMPK	A potent, reversible AMPK activator and inhibits fatty acid synthesis.
**Abiraterone (CB-7598)**	P450 (e.g. CYP17)	A potent CYP17 inhibitor.
**Abitrexate (Methotrexate)**	DHFR	An antimetabolite and antifolate agent with antineoplastic and immunosuppressant activities.
**ABT-263 (Navitoclax)**	Bcl-2	A potent inhibitor of Bcl-xL, Bcl-2 and Bcl-w.
**ABT-737**	Autophagy	A BH3 mimetic inhibitor of Bcl-xL, Bcl-2 and Bcl-w.
**ABT-751**	Microtubule Associated	It binds to the colchicine site on β-tubulin and inhibits polymerization of microtubules.
**ABT-888 (Veliparib)**	PARP	A potent inhibitor of PARP1 and PARP2
**Adrucil (Fluorouracil)**	DNA/RNA Synthesis	An DNA/RNA synthesis inhibitor, which interrupts nucleotide synthetic by inhibiting thymidylate synthase (TS) in tumor cells
**AEE788 (NVP-AEE788)**	EGFR	A potent inhibitor of EGFR and HER2/ErbB2 and also inhibits VEGFR2 and Flt.
**Afatinib (BIBW2992)**	EGFR	It irreversibly inhibits EGFR/HER2 including EGFRwt, EGFRL858R, EGFRL858R/T790M and HER2.
**AG14361**	PARP	A potent inhibitor of PARP1.
**Altretamine (Hexalen)**	DNA alkylator	An alkylating antineoplastic agent.
**AMG 900**	Aurora Kinase	A potent and highly selective pan-Aurora inhibitor for Aurora A/B/C
**Aminoglutethimide (Cytadren)**	Aromatase	An aromatase inhibitor.
**Amuvatinib (MP-470)**	c-Kit	A potent and multi-targeted inhibitor of c-Kit, PDGFα and FLT3
**Anagrelide HCl**	PDE	A drug used for the treatment of essential thrombocytosis.
**Anastrozole**	Aromatase	A third-generation nonsteroidal selective aromatase inhibitor
**Andarine (GTX-007)**	Androgen Receptor	A selective nonsteroidal androgen receptor (AR) agonist
**APO866 (FK866)**	NMPRTase	An inhibitor of Nicotinamide phosphoribosyltransferase (NMPRTase)
**Aprepitant (MK-0869)**	Neurokinin-1 receptor	A potent and selective neurokinin-1 receptor antagonist
**AR-42 (HDAC-42)**	HDAC	A pan-HDAC inhibitor.
**AT-406**	XIAP, cIAP1, and cIAP2	An antagonist of IAP (inhibitor of apoptosis protein via E3 ubiquitin ligase)
**AT7519**	CDK	A multi-CDK inhibitor for CDK1/CyclinB, CDK2/CyclinA, CDK3/CyclinE, CDK4/CyclinD1, CDK6/CyclinD3 and CDK9/CyclinT
**AT9283**	JAK2/3	A potent JAK2/3, Aurora A/B and Abl inhibitor
**AUY922 (NVP-AUY922)**	HSP (e.g. HSP90)	A highly potent HSP90 inhibitor for HSP90α and HSP90β.
**Axitinib**	c-Kit	A multi-target inhibitor of VEGFR1, VEGFR2, VEGFR3, PDGFRβ and c-Kit.
**AZ 3146**	Kinesin	A selective Mps1 inhibitor.
**AZ628**	Raf	A pan-Raf inhibitor for B-Raf, B-RafV600E and C-Raf
**Azacitidine (Vidaza)**	DNA Methyltransferase	A nucleoside analogue of cytidine that specifically inhibits DNA methylation by trapping DNA methyltransferases.
**Azathioprine (Azasan, Imuran)**	Rac1	An immunosuppressive drug, inhibiting purine synthesis and GTP-binding protein Rac1 activation, used in the treatment of organ transplantation and autoimmune diseases.

**AZD6244 (Selumetinib)**	MEK	A highly selective MEK1 inhibitor.
**AZD7762**	Chk	A potent and selective Chk1 and Chk2 inhibitor.
**AZD8055**	mTOR	A novel ATP-competitive inhibitor of mTOR.
**Barasertib (AZD1152-HQPA)**	Aurora Kinase	A highly selective Aurora B inhibitor.
**BAY 11-7082 (BAY 11-7821)**	IkBa phosphorylation	An irreversible inhibitor of TNFα-induced IκBα phosphorylation
**Belinostat (PXD101)**	HDAC	A novel HDAC inhibitor.
**Bendamustine HCL**	DNA synthesis	A DNA-damaging agent.
**Betapar (Meprednisone)**	Glucocorticoid receptor	A glucocorticoid and a methylated derivative of prednisone.
**Bexarotene**	RXR	A retinoid specifically selective for retinoid X receptors, used as an oral antineoplastic agent in the treatment of cutaneous T-cell lymphoma.
**BEZ235 (NVP-BEZ235)**	ATM/ATR	A dual ATP-competitive PI3K and mTOR inhibitor of p110α, p110γ, p110δ and p110β and also inhibits ATR.
**BI 2536**	PLK	A potent Plk1 inhibitor
**BI6727 (Volasertib)**	PLK	A highly potent Plk1 inhibitor.
**BIBF1120 (Vargatef)**	VEGFR/FGFR	A potent triple angiokinase inhibitor for VEGFR1/2/3, FGFR1/2/3 and PDGFRα/β.
**BIBR 1532**	Telomerase	A potent, selective non-competitive telomerase inhibitor.
**Bicalutamide (Casodex)**	Androgen Receptor	An androgen receptor (AR) antagonist.
**BIIB021**	HSP (e.g. HSP90)	A synthetic small-molecule inhibitor of HSP90.
**BIRB 796 (Doramapimod)**	p38 MAPK	A highly selective p38α MAPK inhibitor
**BKM120 (NVP-BKM120)**	PI3K	A selective PI3K inhibitor of p110α, p110β, p110δ and p110γ.
**Bleomycin sulfate**	DNA/RNA Synthesis	A glycopeptide antibiotic and an anticancer agent for squamous cell carcinomas (SCC).
**BMS 777607**	Axl	A Met-related inhibitor for c-Met, Axl, Ron and Tyro3
**BMS 794833**	c-Met	A potent ATP competitive inhibitor of Met and VEGFR2
**BMS-599626 (AC480)**	HER1/2	A selective and efficacious inhibitor of HER1 and HER2
**Bortezomib (Velcade)**	Proteasome	A potent 20S proteasome inhibitor.
**Bosutinib (SKI-606)**	Src	A novel, dual Src/Abl inhibitor
**Brivanib (BMS-540215)**	VEGFR/FGFR	An ATP-competitive inhibitor against human VEGFR2 and FGFR
**BTZ043 racemate**	DprE	A decaprenylphosphoryl-β-D-ribose 2'-epimerase (DprE1) inhibitor acting as a new antimycobacterial agent that kill Mycobacterium tuberculosis.
**Busulfan (Myleran, Busulfex)**	DNA alkylator	A cell cycle non-specific alkylating antineoplastic agent.
**BX-795**	PDK1	A potent and specific PDK1 inhibitor
**CAL-101 (GS-1101)**	PI3K	A selective PI3K class I inhibitor of p110δ
**Canagliflozin**	SGLT	A highly potent and selective SGLT2 inhibitor
**Capecitabine (Xeloda)**	DNA/RNA Synthesis	A tumor-selective fluoropyrimidine carbamate, which achieves higher intratumoral 5-FU level with lower toxicity than 5-FU.
**Carboplatin**	DNA/RNA Synthesis	A DNA synthesis inhibitor by binding to DNA and interfering with the cell's repair mechanism.
**Carmofur**	Acid ceramidase	A highly potent acid ceramidase inhibitor, used in the treatment of breast and colorectal cancer.
**Cediranib (AZD2171)**	VEGFR	A highly potent VEGFR(KDR) inhibitor and also inhibits Flt1/4
**Celecoxib**	COX	A selective COX-2 inhibitor
**CEP33779**	JAK	A selective JAK2 inhibitor
**CH5132799**	PI3Ks	It exhibits a strong inhibitory activity especially against PI3Kα
**CHIR-99021 (CT99021) HCl**	GSK-3	A GSK-3α and GSK-3β inhibitor
**Chrysophanic acid (Chrysophanol)**	EGFR	A EGFR/mTOR pathway inhibitor.
**CI-1040 (PD184352)**	MEK	An ATP non-competitive MEK1/2 inhibitor
**Cisplatin**	DNA/RNA Synthesis	An inorganic platinum complex, which is able to inhibit DNA synthesis by conforming DNA adducts in tumor cells

**Cladribine**	DNA/RNA Synthesis	An adenosine deaminase inhibitor
**Clafen (Cyclophosphamide)**	DNA/RNA Synthesis	A nitrogen mustard alkylating agent
**Clofarabine**	DNA/RNA Synthesis	It inhibits the enzymatic activities of ribonucleotide reductase and DNA polymerase
**Coenzyme Q10 (CoQ10)**	Other	It is a component of the electron transport chain and participates in aerobic cellular respiration.
**CP-466722**	ATM/ATR	A potent and reversible ATM inhibitor.
**Crenolanib (CP-868596)**	PDGFR	A potent and selective inihibitor of PDGFRα/b
**Crizotinib (PF-02341066)**	ALK	A potent inhibitor of c-Met and ALK
**CUDC-101**	HDAC	A potent multi-target inhibitor targeting HDAC, EGFR and HER2
**CX-4945 (Silmitasertib)**	CK2	A potent and selective inhibitor of CK2 (casein kinase 2)
**CYC116**	Aurora Kinase	A potent inhibitor of Aurora A/B and VEGFR
**Cyclopamine**	Hedgehog	A specific Hedgehog (Hh) signaling pathway antagonist of Smoothened (Smo)
**Cyclophosphamide monohydrate**	DNA alkylator	A nitrogen mustard alkylating agent, it attaches the alkyl group to the guanine base of DNA, , shown to crosslink DNA, causing strand breakage and inducing mutations
**Cyclosporin A (Cyclosporine A)**	calcineurin	An immunosuppressive agent, binds to the cyclophilin and then inhibits calcineurin, widely used in organ transplantation to prevent rejection.
**Cyt387**	JAK	An ATP-competitive inhibitor of JAK1 and JAK2
**Cytarabine**	DNA/RNA Synthesis	An antimetabolic agent and DNA synthesis inhibitor
**Dacarbazine (DTIC-Dome)**	DNA/RNA Synthesis	A triazene derivative with antineoplastic activity. Dacarbazine alkylates and cross-links DNA during all phases of the cell cycle, resulting in disruption of DNA function, cell cycle arrest, and apoptosis; used in the treatment of various cancers.
**Dacomitinib (PF299804,PF-00299804)**	EGFR	A potent and irreversible pan-ErbB inhibitor against ErbB1, ErbB2 and ErbB4
**Dalcetrapib (JTT-705)**	CETP	A rhCETP inhibitor
**Danusertib (PHA-739358)**	Aurora Kinase	An Aurora inhibitor for Aurora A/B/C, Bcr-Abl, c-RET and FGFR
**Dapagliflozin**	SGLT	A potent and selective hSGLT2 inhibitor
**DAPT (GSI-IX)**	Beta Amyloid	A novel γ-secretase inhibitor, which inhibits Aβ production
**Dasatinib (BMS-354825)**	Bcr-Abl	A novel, potent and multi-targeted inhibitor that targets Abl, Src and c-Kit
**Daunorubicin HCl (Daunomycin HCl)**	Topoisomerase	It inhibits both DNA and RNA synthesis and inhibits DNA synthesis
**DCC-2036 (Rebastinib)**	Bcr-Abl	A conformational control Bcr/Abl inhibitor of Abl1(WT) and Abl1(T315I)
**Decitabine**	DNA Methyltransferase	A DNA methyltransferase inhibitor, incorporating into DNA and resulting in hypomethylation of DNA and intra-S-phase arrest of DNA replication. It is used to treat myelodysplastic syndrome (MDS).
**Deforolimus (Ridaforolimus)**	mTOR	A selective mTOR inhibitor
**Desmethyl Erlotinib (CP-473420)**	EGFR	A free base of OSI-420, which is an active metabolite of Erlotinib which is an orally active EGFR inhibitor for human EGFR
**Dexamethasone**	Autophagy	A potent synthetic member of the glucocorticoid class of steroid drugs, and an interleukin receptor modulator that has anti-inflammatory and immunosuppressant effects
**Dexamethasone acetate**	Autophagy	A potent synthetic member of the glucocorticoid class of steroid drugs, and an interleukin receptor modulator that has anti-inflammatory and immunosuppressant effects
**D-glutamine**	GluR	A D type stereoisomer of glutamine which is one of the 20 amino acids encoded by the standard genetic code.
**Dimesna**	Other	An uroprotective agent used to decrease urotoxicity.
**Disulfiram (Antabuse)**	Dehydrogenase	A specific inhibitor of aldehyde-dehydrogenase (ALDH1), used for the treatment of chronic alcoholism by producing an acute sensitivity to alcohol.
**DMXAA (ASA404)**	VDA	A vascular disrupting agents (VDA) and competitive inhibitor of DT-diaphorase
**Docetaxel (Taxotere)**	Microtubule Associated	An analog of paclitaxel, is an inhibitor of depolymerisation of microtubules by binding to stabilized microtubules.

**Dorzolamide HCl**	Carbonic Anhydrase	A water-soluble, potent inhibitor of human carbonic anhydrase II and IV used as anti-glaucoma agent.
**Dovitinib (TKI-258)**	VEGFR	A multi-target RTK inhibitor for Flt3, c-Kit, FGFR1/3, VEGFR1/2/3/4, PDGFRα/β
**Doxercalciferol (Hectorol)**	Vitamin	A synthetic analog of vitamin D2, suppressiong parathyroid synthesis and secretion, used to treat secondary hyperparathyroidism and metabolic bone disease.
**Doxorubicin (Adriamycin)**	Topoisomerase	An antibiotic agent that inhibits DNA topoisomerase II and induces DNA damage and apoptosis in tumor cells
**E7080 (Lenvatinib)**	VEGFR	A multi-target inhibitor, mostly for VEGFR2(KDR)/VEGFR3(Flt-4)
**Elesclomol**	HSP (e.g. HSP90)	A novel potent oxidative stress inducer that illicits pro-apoptosis events among tumor cells.
**ENMD-2076**	Aurora Kinase	A selective inhibitor of Aurora A and Flt3
**Entinostat (MS-275, SNDX-275)**	HDAC	An HDAC inhibitor of HDAC1 and HDAC3
**Enzastaurin (LY317615)**	PKC	A potent PKCβ selective inhibitor
**Epirubicin HCl**	Topoisomerase	A semisynthetic L-arabino derivative of doxorubicin, is an antineoplastic agent by inhibiting Topoisomerase.
**Epothilone A**	Microtubule Associated	A paclitaxel-like microtubule-stabilizing agent
**Epothilone B (EPO906)**	Microtubule Associated	A paclitaxel-like microtubule-stabilizing agent
**Erlotinib HCl**	Autophagy	An EGFR inhibitor
**Estradiol**	Estrogen/progestogen Receptor	A human sex hormone and steroid
**Estrone**	Estrogen/progestogen Receptor	An estrogenic hormone.
**Etoposide (VP-16)**	Topoisomerase	A semisynthetic derivative of podophyllotoxin, which inhibits DNA synthesis via topoisomerase II inhibition activity.
**Everolimus (RAD001)**	mTOR	An mTOR inhibitor of FKBP12
**Evista (Raloxifene HCl)**	Estrogen/progestogen Receptor	An estrogen antagonists, which inhibits human cytosolic aldehyde oxidase-catalyzed phthalazine oxidation activity
**EX 527**	Sirtuin	A potent and selective SIRT1 inhibitor
**Exemestane**	Aromatase	An aromatase inhibitor, inhibits human placental and rat ovarian aromatase
**Ezetimibe (Zetia)**	LDL	A potent, selective, cholesterol absorption inhibitor, used to lower cholesterol
**Febuxostat (Uloric)**	ROS	A selective xanthine oxidase inhibitor
**Fingolimod (FTY720)**	S1P Receptor	A S1P antagonist
**Flavopiridol (Alvocidib) HCl**	CDK	It competes with ATP to inhibit CDKs including CDK1, CDK2, CDK4 and CDK6
**Floxuridine (Fludara)**	DNA/RNA Synthesis	An antineoplastic antimetabolite, used in the treatment of colon carcinoma and colorectal cancer that has metastasized to the liver.
**Fludarabine (Fludara)**	DNA/RNA Synthesis	A STAT1 activation inhibitor and a DNA synthesis inhibitor.
**Fludarabine Phosphate (Fludara)**	DNA/RNA Synthesis	A STAT-1 activation inhibitor and a DNA synthesis inhibitor
**Flutamide (Eulexin)**	Androgen Receptor	An oral nonsteroidal antiandrogen agent primarily used to treat prostate cancer.
**Fluvastatin sodium (Lescol)**	HMG-CoA Reductase	It inhibits HMG-CoA reductase activity
**Formestane**	Aromatase	A second generation selective aromatase inhibitor
**Ftorafur**	DNA/RNA Synthesis	A substance being used in the treatment of some types of cancer.
**Fulvestrant (Faslodex)**	Estrogen/progestogen Receptor	An estrogen receptor (ER) antagonist
**Ganetespib (STA-9090)**	HSP (e.g. HSP90)	An HSP90 inhibitor
**GDC-0879**	Raf	A novel, potent and selective B-Raf inhibitor
**GDC-0941**	PI3K	A potent inhibitor of PI3Kα and PI3Kδ
**Gefitinib (Iressa)**	EGFR	An EGFR inhibitor for Tyr1173, Tyr992, Tyr1173 and Tyr992
**Geldanamycin**	Autophagy	A natural existing HSP90 inhibitor, specifically disrupts glucocorticoid receptor (GR)/HSP association.

**Gemcitabine (Gemzar)**	Autophagy	A nucleic acid synthesis inhibitor, is a very potent and specific deoxycytidine analogue, used as chemotherapy.
**Gemcitabine HCl (Gemzar)**	Autophagy	A DNA synthesis inhibitor
**Gossypol**	Dehydrogenase	A polyphenolic aldehyde that permeates cells and acts as an inhibitor for several dehydrogenase enzymes.
**GSK1120212 (Trametinib)**	MEK	A highly specific and potent MEK1 and MEK2 inhibitor
**GSK1904529A**	IGF-1R	A selective inhibitor of IGF-1R and IR
**GSK2126458**	mTOR	A highly selective and potent inhibitor of p110α, p110β, p110γ, p110δ, mTORC1 and mTORC2
**GSK461364**	PLK	It inhibits purified Plk1
**GSK690693**	Akt	A pan-Akt inhibitor targeting Akt1, Akt2 and Akt3
**GW3965 HCl**	Liver X Receptor	A potent, selective LXR agonist for hLXRα hLXRb
**GW4064**	FXR	An agonist of farnesoid X receptor (FXR)
**Hydrocortisone (Cortisol)**	Glucocorticoid receptor	A steroid hormone or glucocorticoid produced by the adrenal gland.
**Hydroxyurea (Cytodrox)**	DNA/RNA Synthesis	An antineoplastic agent that inhibits DNA synthesis through the inhibition of ribonucleoside diphosphate reductase.
**IC-87114**	PI3K	A selective inhibitor of PI3Kδ
**Idarubicin HCl**	Topoisomerase	A hydrochloride salt form of Idarubicin which is an anthracycline antibiotic and a DNA topoisomerase II (topo II) inhibitor
**Ifosfamide**	DNA/RNA Synthesis	A nitrogen mustard alkylating agent used in the treatment of cancer.
**Imatinib (Gleevec)**	Bcr-Abl	A multi-target inhibitor of tyrosine kinase with inhibition for v-Abl, c-Kit and PDGFR
**Imatinib Mesylate**	Bcr-Abl	It is orally bioavailability mesylate salt of Imatinib, which is a multi-target inhibitor of v-Abl, c-Kit and PDGFR
**Imiquimod**	Other	A novel synthetic agent with immune response modifying activity.
**Iniparib (BSI-201)**	PARP	A PARP1 inhibitor with demonstrated effectiveness in triple-negative breast cancer (TNBC)
**INK 128 (MLN0128)**	mTOR	A potent and selective mTOR inhibitor
**Irinotecan**	Topoisomerase	A topoisomerase I inhibitor
**Irinotecan HCl Trihydrate (Campto)**	Topoisomerase	It prevents DNA from unwinding by inhibition of topoisomerase 1.
**Isotretinoin**	Hydroxylase	It was developed to be used as a chemotherapy medication for the treatment of brain cancer, pancreatic cancer and more.
**Ispinesib (SB-715992)**	Kinesin	A potent, specific and reversible inhibitor of kinesin spindle protein (KSP)
**Itraconazole (Sporanox)**	P450	A relatively potent inhibitor of CYP3A4, used as a triazole antifungal agent.
**JNJ 26854165 (Serdemetan)**	E3 Ligase	A HDM2 ubiquitin ligase antagonist and also induces early apoptosis in p53 wild-type cells, inhibits cellular proliferation followed by delayed apoptosis in the absence of functional p53
**JNJ-26481585**	HDAC	A novel second-generation HDAC inhibitor with highest potency for HDAC1
**JNJ-38877605**	c-Met	An ATP-competitive inhibitor of c-Met
**JNJ-7706621**	Aurora Kinase	A pan-CDK inhibitor with the highest potency on CDK1/2 and also potently inhibits Aurora A/B
**Ku-0063794**	mTOR	A potent and highly specific dual-mTOR inhibitor of mTORC1 and mTORC2
**KU-55933**	ATM/ATR	A potent and specific ATM inhibitor
**KU-60019**	ATM/ATR	A potent and specific ATM inhibitor
**KX2-391**	Src	A highly selective non ATP-competitive Src inhibitor in cancer cell lines.
**Lapatinib**	HER2	Used in the form of Lapatinib Ditosylate, is a potent EGFR and ErbB2 inhibitor
**Lapatinib Ditosylate (Tykerb)**	HER2	A potent EGFR and ErbB2 inhibitor
**LDE225 (NVP-LDE225, Erismodegib)**	Hedgehog/Smoothened	A Smoothened (Smo) antagonist, inhibiting Hedgehog
**LDN193189**	TGF-beta/Smad	A selective BMP signaling inhibitor, inhibits the transcriptional activity of the BMP type I receptors ALK2 and ALK3

**Lenalidomide (Revlimid)**	TNF-alpha	A TNF-α secretion inhibitor
**Letrozole**	Aromatase	A third generation inhibitor of aromatase
**Leucovorin Calcium**	Other	A derivative of folic acid, which can be used to increase levels of folic acid under conditions favoring folic acid inhibition
**Linifanib (ABT-869)**	CSF-1R	A novel, potent ATP-competitive VEGFR/PDGFR inhibitor for KDR, CSF-1R, Flt-1/3 and PDGFRβ
**Linsitinib (OSI-906)**	IGF-1R	A selective inhibitor of IGF-1R and IR
**Lomustine (CeeNU)**	DNA/RNA Synthesis	It inhibits cancer cells by damaging the DNA and stops cells from dividing.
**Lonidamine**	Hexokinase	An orally administered small molecule hexokinase inactivator
**LY2109761**	TGF-beta/Smad	A novel selective TGF-β receptor type I/II (TβRI/II) dual inhibitor
**LY2157299**	TGF-beta/Smad	A potent TGFβ receptor I (TβRI) inhibitor
**LY2228820**	p38 MAPK	A novel and potent inhibitor of p38 MAPK
**LY2603618 (IC-83)**	Chk	A selective Chk1 inhibitor with potential anti-tumor activity.
**LY294002**	Autophagy	A PI3K inhibitor for p110α, p110δ and p110β and also blocks autophagosome formation.
**Maraviroc**	CCR	A CCR5 antagonist for MIP-1α, MIP-1β and RANTES
**Masitinib (AB1010)**	c-Kit	A novel tyrosine kinases inhibitor for Kit and PDGFRα/β
**MDV3100 (Enzalutamide)**	Androgen Receptor	An androgen-receptor (AR) antagonist
**Medroxyprogesterone acetate**	Estrogen/progestogen Receptor	A synthetic progestin and act as a progesterone receptor agonist.
**Megestrol Acetate**	Androgen Receptor	A synthetic progesteronal agent
**Mercaptopurine**	DNA/RNA Synthesis	A widely used antileukemic agent and immunosuppressive drug that inhibits de novo purine synthesis through incorporation of thiopurine methyltransferase metabolites into DNA and RNA.
**Mesna (Uromitexan, Mesnex)**	Other	A sulfhydryl compound that is used to reduce the incidence of hemorrhagic cystitis associated with certain chemotherapeutic agents.
**Mifepristone (Mifeprex)**	Estrogen/progestogen Receptor	A remarkably active antagonist of progesterone receptor and glucocorticoid receptor
**Mitoxantrone HCl**	Topoisomerase	A type II topoisomerase inhibitor
**MK-0752**	Gamma-secretase	A moderately potent γ-secretase inhibitor
**MK-1775**	Wee1	A potent and selective Wee1 inhibitor
**MK-2206 2HCl**	Akt	A highly selective inhibitor of Akt1, Akt2 and Akt3
**MLN2238**	Proteasome	It inhibits the chymotrypsin-like proteolytic (β5) site of the 20S proteasome
**MLN8237 (Alisertib)**	Aurora Kinase	A selective Aurora A inhibitor
**MLN9708**	Proteasome	A selective inhibitor of chymotrypsin-like proteolytic (β5) site of the 20S proteasome
**Mocetinostat (MGCD0103)**	HDAC	A potent HDAC inhibitor for HDAC1, HDAC2 and HDAC3
**Motesanib Diphosphate (AMG-706)**	PDGFR	A potent ATP-competitive inhibitor of VEGFR1/2/3, PDGFR, c-Kit and Ret
**Mycophenolate mofetil (CellCept)**	Dehydrogenase	A non-competitive, selective and reversible inhibitor of inosine monophosphate dehydrogenase I/II
**Mycophenolic (Mycophenolate)**	Dehydrogenase	A potent IMPDH inhibitor and the active metabolite of an immunosuppressive drug, used to prevent rejection in organ transplantation.
**Nelarabine (Arranon)**	DNA/RNA Synthesis	A purine nucleoside analog and DNA synthesis inhibitor in tumor cells.
**Neratinib (HKI-272)**	HER2	A highly selective HER2 and EGFR inhibitor
**Nilotinib (AMN-107)**	Bcr-Abl	A selective Bcr-Abl inhibitor
**Nocodazole**	Autophagy	A rapidly-reversible inhibitor of microtubule polymerization, also inhibits Abl, Abl(E255K) and Abl(T315I)
**NU7441 (KU-57788)**	DNA-PK	A highly potent and selective DNA-PK inhibitor and also inhibits PI3K
**Nutlin-3**	E3 Ligase	A potent and selective Mdm2 (RING finger-dependent ubiquitin protein ligase for itself and p53) antagonist
**NVP-BSK805 2HCl**	JAK	A potent and selective ATP-competitive JAK2 inhibitor

**Obatoclax mesylate (GX15-070)**	Autophagy	An antagonist of Bcl-2
**Olaparib (AZD2281)**	PARP	A selective inhibitor of PARP1 and PARP2
**OSI-420**	EGFR	An active metabolite of Erlotinib which is an orally active EGFR inhibitor for inhibition of human EGFR and EGFR autophosphorylation
**OSI-930**	c-Kit	A potent inhibitor of Kit, KDR, Flt, CSF-1R, c-Raf and Lck
**Ostarine (MK-2866)**	Androgen Receptor	A selective androgen receptor modulator (SARM)
**Oxaliplatin (Eloxatin)**	DNA/RNA Synthesis	It inhibits DNA synthesis by conforming DNA adducts.
**PAC-1**	Caspase	A potent procaspase-3 activator and the first small molecule known to directly activate procaspase-3 to caspase-3.
**Paclitaxel (Taxol)**	Microtubule Associated	A microtubule polymer stabilizer in human endothelial cells.
**Palomid 529**	mTOR	It inhibits both the mTORC1 and mTORC2 complexes, reduces phosphorylation of pAktS473, pGSK3βS9, and pS6
**Pamidronate Disodium**	Other	A nitrogen containing bisphosphonate, used to prevent osteoporosis.
**Pazopanib**	VEGFR	A potent and selective multi-targeted receptor tyrosine kinase inhibitor of VEGFR1, VEGFR2, VEGFR3, PDGFR, FGFR and c-Kit.
**PCI-24781**	HDAC	A novel pan-HDAC inhibitor mostly targeting HDAC1
**PCI-32765 (Ibrutinib)**	BTK	A potent and highly selective Brutons tyrosine kinase (Btk) inhibitor
**PD 0332991 (Palbociclib) HCl**	CDK	A highly selective inhibitor of CDK4/cyclin D1 and CDK6/cyclin D2
**PD0325901**	MEK	A selective and non ATP-competitive MEK inhibitor
**PD153035 HCl**	EGFR	A potent and specific inhibitor of EGFR
**PD173074**	VEGFR	A potent FGFR1 inhibitor and also inhibits VEGFR2
**Pelitinib (EKB-569)**	EGFR	A potent irreversible EGFR inhibitor
**Pemetrexed (Alimta)**	DHFR	A novel antifolate and antimetabolite for TS, DHFR and GARFT
**PF 573228**	FAK	an ATP-competitive inhibitor of FAK
**PF-03814735**	Aurora Kinase	A novel, potent and reversible inhibitor of both Aurora A and Aurora B
**PF-04217903**	c-Met	A selective ATP-competitive c-Met inhibitor
**PF-3845**	FAAH	A potent, selective and irreversible FAAH inhibitor
**PF-562271**	FAK	A potent, ATP-competitive, reversible inhibitor of FAK and Pyk2
**PH-797804**	p38 MAPK	A novel pyridinone inhibitor of p38α
**PHA-665752**	c-Met	A potent, selective and ATP-competitive c-Met inhibitor
**PHA-793887**	CDK	A novel and potent inhibitor of CDK2, CDK5 and CDK7
**Phloretin (Dihydronaringenin)**	SGLT	A dihydrochalcone, a type of polyphenol.
**PI-103**	Autophagy	A multi-targeted PI3K inhibitor for p110α/β/δ/γ
**PIK-75**	DNA-PK	A selective and competitive inhibitor of p110α
**PIK-90**	PI3K	A potent inhibitor of p110α, p110γ and p110δ
**PIK-93**	PI3K	A novel and potent inhibitor of PI3Kγ and PI4KIIIβ
**Pioglitazone (Actos)**	P450	A selective peroxisome proliferator-activated receptor gamma (PPARgamma) agonist
**Pomalidomide**	TNF-alpha	It inhibits LPS-induced TNF-α release
**Ponatinib (AP24534)**	FGFR	A novel, potent multi-target inhibitor of Abl, PDGFRα, VEGFR2, FGFR1 and Src
**Prednisone (Adasone)**	Glucocorticoid receptor	A synthetic corticosteroid agent that is particularly effective as an immunosuppressant compound.
**Procarbazine HCl (Matulane)**	DNA/RNA Synthesis	A hydrochloride salt form of procarbazine which is a polyfunctional alkylating compound used as an antineoplastic agent.
**Quercetin (Sophoretin)**	PI3K	A stimulator of recombinant SIRT1 and also a PI3K inhibitor
**Quizartinib (AC220)**	FLT3	A second-generation FLT3 inhibitor for Flt3(ITD/WT)
**R406 (free base)**	Syk	A Syk inhibitor

**R935788 (Fostamatinib disodium, R788 disodium)**	Syk	A prodrug of the active metabolite R406, is a Syk inhibitor
**Raltitrexed (Tomudex)**	DNA/RNA Synthesis	A thymidylate synthase inhibitor
**Ranolazine (Ranexa)**	Calcium channel	A calcium uptake inhibitor via the sodium/calcium channel
**Rapamycin (Sirolimus)**	Autophagy	A specific mTOR inhibitor
**Regorafenib (BAY 73-4506)**	VEGFR	A multi-target inhibitor for VEGFR1, VEGFR2, VEGFR3, PDGFRβ, Kit, RET and Raf-1
**RG108**	DNA Methyltransferase	An inhibitor of DNA methyltransferase
**Rigosertib (ON-01910)**	PLK	A non-ATP-competitive inhibitor of PLK1 (Polo-like kinase 1)
**Roscovitine (Seliciclib, CYC202)**	CDK	A potent and selective CDK inhibitor for Cdc2/cyclin B, CDK2/cyclin A, CDK2/cyclin E and CDK5/p53
**Rosiglitazone (Avandia)**	PPAR	A potent antihyperglycemic agent and a potent thiazolidinedione insulin sensitizer.
**Rucaparib (AG-014699 , PF-01367338)**	PARP	An inhibitor of PARP
**Ruxolitinib (INCB018424)**	JAK	A potent ans selective JAK1/2 inhibitor
**Salinomycin (Procoxacin)**	Wnt/beta-catenin	A traditionally used as an anti-coccidial drug, has recently been shown to possess anti-cancer and anti-cancer stem cell (CSC) effects
**Saracatinib (AZD0530)**	Scr	A potent Src inhibitor
**SB 203580**	p38 MAPK	A p38 MAPK inhibitor and blocks PKB phosphorylation
**SB 216763**	GSK-3	A potent and selective GSK-3α and GSK-3b inhibitor
**SB 431542**	TGF-beta/Smad	A potent and selective inhibitor of ALK5
**SB 525334**	TGF-beta/Smad	A potent and selective inhibitor of TGFβ receptor I (ALK5)
**SB 743921**	Kinesin	A Kinesin spindle protein (KSP) inhibitor
**SB590885**	Raf	A potent B-Raf inhibitor
**SB939 (Pracinostat)**	HDAC	A potent pan-HDAC inhibitor
**SGI-1776 free base**	Pim	A novel ATP competitive inhibitor to Pim1, Pim2 and Pim3
**SGX-523**	c-Met	A selective Met inhibitor
**Simvastatin (Zocor)**	HMG-CoA Reductase	A competitive inhibitor of HMG-CoA reductase
**Sirtinol**	Sirtuin	A specific SIRT1 and SIRT2 inhibtor
**SNS-032 (BMS-387032)**	CDK	A novel, potent and selective CDK inhibitor of CDK2, CDK7 and CDK9
**SNS-314 Mesylate**	Aurora Kinase	A potent and selective inhibitor of Aurora A, Aurora B and Aurora C
**Sodium butyrate**	HDAC	A histone deacetylase inhibitor and competitively binds to the zinc sites of class I and II histone deacetylases (HDACs).
**Sorafenib (Nexavar)**	PDGFR	A multikinase inhibitor of Raf-1, B-Raf and VEGFR-2
**Sotrastaurin (AEB071)**	PKC	A potent selective pan-PKC inhibitor, mostly for PKCθ
**SRT1720**	Sirtuin	A selective SIRT1 activator
**S-Ruxolitinib**	JAK	A JAK family inhibitor for JAK1 and JAK2
**STF-62247**	Autophagy	A molecule targeting VHL-deficient renal cell carcinoma that induces autophagy. It shows selective toxicity and growth inhibition of renal cells lacking VHL
**Streptozotocin (Zanosar)**	DNA alkylator	A glucosamine-nitrosourea derivative, which is a methylating, carcinogenic, antibiotic and diabetes inducing agent.
**SU11274**	c-Met	A selective Met inhibitor
**Sunitinib Malate (Sutent)**	c-Kit	A multi-targeted RTK inhibitor targeting VEGFR2 (Flk-1) and PDGFRβ and also inhibits c-Kit.
**TAE684 (NVP-TAE684)**	ALK	A potent and selective ALK inhibitor
**TAK-733**	MEK	A potent and selective MEK allosteric site inhibitor for MEK1
**TAME**	APC	An APC inhibitor and also inhibits an E3 ubiquitin ligase called "anaphase-promoting complex/cyclosome (APC/C)".
**Tamoxifen Citrate (Nolvadex)**	Autophagy	An antagonist of the estrogen receptor by competitive inhibition of estrogen binding.

**Tandutinib (MLN518)**	FLT3	An ATP-competitive and highly selective inhibitor of Flt3, PDGFR and c-Kit
**Telatinib (BAY 57-9352)**	VEGFR	A potent inhibitor of VEGFR2/3, c-Kit and PDGFRa
**Temozolomide**	Autophagy	A alkylanting agent that induces DNA damage
**Temsirolimus (Torisel)**	mTOR	A specific mTOR inhibitor
**Teniposide (Vumon)**	Topoisomerase	A chemotherapeutic medication mainly used in the treatment of childhood acute lymphocytic leukemia (ALL).
**TG101348 (SAR302503)**	JAK	A selective inhibitor of JAK2
**Thalidomide**	E3 Ligase	A sedative drug,immunomodulatory agent and also is investigated for treating symptoms of many cancers.Thalidomide inhibits an E3 ubiquitin ligase
**Tie2 kinase inhibitor**	Tie-2	A potent and selective Tie2 inhibitor
**Tipifarnib (Zarnestra)**	Transferase	A potent and specific farnesyltransferase (FTase) inhibitor
**Tivozanib (AV-951)**	VEGFR	A potent and selective VEGFR inhibitor for VEGFR1/2/3 and also inhibits PDGFR and c-Kit
**Tofacitinib (CP-690550, Tasocitinib)**	JAK	A novel inhibitor of JAK3
**Topotecan HCl**	Topoisomerase	A topoisomerase I inhibitor for MCF-7 Luc cells and DU-145 Luc cells
**Toremifene Citrate (Fareston, Acapodene)**	Estrogen/progestogen Receptor	An oral selective estrogen receptor modulator (SERM) used in the treatment od advanced breast cancer
**Torin 1**	mTOR	A potent inhibitor of mTOR 1/2
**Torin 2**	ATM/ATR	A highly potent and selective mTOR inhibitor and also exhibits potent cellular activity against ATM/ATR/DNA-PK
**Tosedostat (CHR2797)**	Aminopeptidase	An aminopeptidase inhibitor for LAP, PuSA and Aminopeptidase N
**TPCA-1**	IκB/IKK	An inhibitor of IKK-2 and it inhibits NF-kB pathway
**Tretinoin (Aberela)**	Retinoid receptor	A ligand for both the retinoic acid receptor (RAR) and the retinoid X receptor (RXR), can induce granulocytic differentiation and apoptosis in acute promyelocytic leukemia (APL) cells
**Triamcinolone Acetonide**	Glucocorticoid receptor	A synthetic glucocorticoid, used in the symptomatic treatment of inflammation
**Trichostatin A (TSA)**	HDAC	An HDAC inhibitor
**Triciribine (Triciribine phosphate)**	Akt	A DNA synthesis inhibitor and also inhibits Akt and HIV-1
**Triptolide**	NF-kB	A immunosuppresive agent that acting as NF-κB inhibitor with dual actions by disruption of p65/CBP interaction and by reduction of p65 protein.
**TW-37**	Bcl-2	A novel nonpeptide inhibitor to recombinant Bcl-2, Bcl-xL and Mcl-1
**Ubenimex (Bestatin)**	Other	A potent aminopeptidase-B and leukotriene (LT) A4 hydrolase inhibitor, used in the treatment of acute myelocytic leukemia.
**Valproic acid sodium salt (Sodium valproate)**	Autophagy	A HDAC inhibitor by selectively inducing proteasomal degradation of HDAC2
**Vandetanib (Zactima)**	VEGFR	A potent inhibitor of VEGFR2, VEGFR3 AND EGFR
**Vatalanib 2HCl (PTK787)**	VEGFR	An inhibitor of VEGFR2/KDR
**Vemurafenib (PLX4032)**	Raf	A novel and potent inhibitor of B-RafV600E
**Vinblastine**	AChR	It inhibits microtubule formation and supresses nAChR activity, used to treat certain kinds of cancer
**Vincristine**	Microtubule Associated	An inhibitor of polymerization of microtubules by binding to tubulin
**Vinpocetine (Cavinton)**	Sodium Channel	A selectively inhibitor of voltage-sensitive sodium channel for the treatment of stroke, vascular dementia and Alzheimer's disease.
**Vismodegib (GDC-0449)**	Hedgehog/Smoothened	A potent, novel and specific Hedgehog inhibitor and also inhibits P-gp
**Vorinostat (SAHA)**	Autophagy	An HDAC inhibitor
**VX-680 (MK-0457, Tozasertib)**	Aurora Kinase	A pan-Aurora inhibitor, mostly against Aurora A
**WAY-362450**	FXR	A potent, selective FXR agonist, highly selective versus other nuclear receptors, such as LXR, PPAR, ER

**WP1130**	Bcr-Abl	A selective deubiquitinase (DUB: USP5, UCH-L1, USP9x, USP14, and UCH37) inhibitor and also suppresses Bcr/Abl, also a JAK2 transducer (without affecting 20S proteasome) and activator of transcription (STAT).
**WYE-354**	mTOR	A potent, specific and ATP-competitive inhibitor of mTOR
**WZ4002**	EGFR	A novel, mutant-selective EGFR inhibitor for EGFRL858R and EGFRL858R/T790M
**XAV-939**	Wnt/beta-catenin	A selective Wnt β-catenin-mediated transcription inhibitor for TNKS1 and TNKS2
**XL147**	PI3K	A selective and reversible class I PI3K inhibitor for PI3Kα/d/g
**XL765 (SAR245409)**	mTOR	A dual inhibitor of mTOR/PI3k mostly for p110γ and also inhibits DNA-PK and mTOR
**Y-27632 2HCl**	Autophagy	A selective ROCK1 (p160ROCK) inhibitor
**YM155**	Survivin	A potent survivin suppressant by inhibiting Survivin promoter activity
**YM201636**	PI3K	A selective PIKfyve inhibitor
**YO-01027**	Gamma-secretase	A dipeptidic γ-secretase inhibitor for APPL and Notch cleavage
**Zibotentan (ZD4054)**	Endothelin Receptor	A specific Endothelin A (ETA) antagonist
**Zileuton**	Lipoxygenase	An orally active inhibitor of 5-lipoxygenase, and thus inhibits leukotrienes (LTB4, LTC4, LTD4, and LTE4) formation.
**ZSTK474**	PI3K	A potent pan-class I PI3K inhibitor, mostly PI3Kd
