# Supplementary material for: Joint action of miR‐126 and MAPK/PI3K inhibitors against metastatic melanoma
Source: Mol Oncol. 2019 Aug 6;13(9):1836–54. doi: 10.1002/1878-0261.12506 (PMC6717748; doi:10.1002/1878-0261.12506)
Supplement: Supplementary file 13 — Table S2. Cell viability at 24 and 48 h of treatment with 349 anti‐cancer compounds. [file MOL2-13-1836-s013.pdf]

**Supplementary Table S2.** Cell viability at 24 and 48hrs of treatment with 349 anti-cancer compounds.

| **Viability at 24 hours** | | | | | | | | |
|---|---|---|---|---|---|---|---|---|
| **100 < Viability % > 80** | | | | | | | | |
| | ***Compounds*** | **%** | **s.d.** | | | ***Compounds*** | **%** | **s.d.** |
| **1** | SGX-523 | 154,12 | 1,94 | | **27** | Aminoglutethimide (Cytadren) | 105,9 | 2,56 |
| **2** | MLN8237 (Alisertib) | 154,03 | 4,41 | | **28** | Betapar (Meprednisone) | 105,8 | 1,63 |
| **3** | KU-55933 | 147,49 | 2,22 | | **29** | Mesna (Uromitexan, Mesnex) | 105,7 | 3,18 |
| **4** | BTZ043 racemate | 144,94 | 2,54 | | **30** | Triamcinolone Acetonide | 105,7 | 1,28 |
| **5** | LY294002 | 129,31 | 5,73 | | **31** | Zibotentan (ZD4054) | 105,5 | 5,10 |
| **6** | GSK690693 | 113,32 | 3,54 | | **32** | Barasertib (AZD1152-HQPA) | 105,38 | 1,72 |
| **7** | JNJ-38877605 | 113,27 | 1,94 | | **33** | Ruxolitinib (INCB018424) | 105,4 | 4,43 |
| **8** | Triciribine (Triciribine phosphate) | 111,90 | 3,13 | | **34** | Sirtinol | 105,27 | 5,02 |
| **9** | Hydrocortisone (Cortisol) | 111,8 | 4,21 | | **35** | Dexamethasone | 105,2 | 2,83 |
| **10** | Fludarabine (Fludara) | 111,7 | 0,10 | | **36** | Prednisone (Adasone) | 105,0 | 2,56 |
| **11** | GSK1904529A | 111,33 | 0,46 | | **37** | XL765 (SAR245409) | 104,9 | 6,51 |
| **12** | XL147 | 111,10 | 5,50 | | **38** | Coenzyme Q10 (CoQ10) | 104,82 | 4,84 |
| **13** | Cisplatin | 110,22 | 1,30 | | **39** | PF-04217903 | 104,76 | 2,81 |
| **14** | E7080 (Lenvatinib) | 109,53 | 8,42 | | **40** | A-769662 | 104,73 | 2,87 |
| **15** | Valproic acid sodium salt (Sodium valproate) | 109,41 | 1,30 | | **41** | XAV-939 | 104,51 | 3,02 |
| **16** | Vatalanib 2HCl (PTK787) | 109,27 | 5,21 | | **42** | Pomalidomide | 104,3 | 3,04 |
| **17** | Estradiol | 108,8 | 5,02 | | **43** | Doxercalciferol (Hectorol) | 104,3 | 11,54 |
| **18** | Busulfan (Myleran, Busulfex) | 108,8 | 1,42 | | **44** | BIRB 796 (Doramapimod) | 104,3 | 1,09 |
| **19** | Capecitabine (Xeloda) | 108,79 | 1,25 | | **45** | Quercetin (Sophoretin) | 104,20 | 5,05 |
| **20** | Ezetimibe (Zetia) | 107,9 | 2,72 | | **46** | Anastrozole | 104,07 | 3,48 |
| **21** | Estrone | 107,7 | 1,61 | | **47** | Roscovitine (Seliciclib, CYC202) | 104,04 | 15,34 |
| **22** | Rucaparib (AG-014699 , PF-01367338) | 107,54 | 5,59 | | **48** | Phloretin (Dihydronaringenin) | 103,89 | 1,13 |
| **23** | Tretinoin (Aberela) | 107,1 | 1,03 | | **49** | Pioglitazone (Actos) | 103,67 | 2,07 |
| **24** | Ostarine (MK-2866) | 106,36 | 4,36 | | **50** | Dapagliflozin | 103,5 | 14,21 |
| **25** | Febuxostat (Uloric) | 106,1 | 1,13 | | **51** | GW3965 HCl | 103,34 | 1,02 |
| **26** | PCI-32765 (Ibrutinib) | 106,01 | 3,09 | | **52** | Formestane | 103,1 | 0,23 |

| | | | | | | | | |
|---|---|---|---|---|---|---|---|---|
| **53** | Vinblastine | 102,89 | 3,10 | | **84** | LDE225 (NVP-LDE225, Erismodegib) | 100,5 | 20,36 |
| **54** | BIBR 1532 | 102,87 | 4,36 | | **85** | Vismodegib (GDC-0449) | 100,48 | 1,29 |
| **55** | RG108 | 102,85 | 4,41 | | **86** | Zileuton | 100,5 | 0,22 |
| **56** | Dacarbazine (DTIC-Dome) | 102,61 | 8,70 | | **87** | Bexarotene | 100,3 | 23,50 |
| **57** | LY2109761 | 102,57 | 3,25 | | **88** | Motesanib Diphosphate (AMG-706) | 100,26 | 15,51 |
| **58** | 3-Methyladenine | 102,55 | 1,95 | | **89** | Andarine (GTX-007) | 100,21 | 3,92 |
| **59** | Dexamethasone acetate | 102,42 | 3,14 | | **90** | Celecoxib | 100,21 | 1,71 |
| **60** | JNJ 26854165 (Serdemetan) | 102,42 | 2,04 | | **91** | Disulfiram (Antabuse) | 100,0 | 1,29 |
| **61** | AT-406 | 102,38 | 0,98 | | **93** | DMXAA (ASA404) | 100,0 | 2,70 |
| **62** | Altretamine (Hexalen) | 102,34 | 2,69 | | **94** | Carboplatin | 99,96 | 0,59 |
| **63** | SB 216763 | 102,33 | 31,29 | | **95** | Brivanib (BMS-540215) | 99,96 | 0,79 |
| **64** | BAY 11-7082 (BAY 11-7821) | 102,28 | 8,52 | | **96** | Lomustine (CeeNU) | 99,9 | 1,02 |
| **65** | Cyclophosphamide monohydrate | 102,3 | 6,48 | | **97** | Palomid 529 | 99,87 | 1,56 |
| **66** | Lenalidomide (Revlimid) | 102,10 | 2,87 | | **98** | PHA-665752 | 99,66 | 1,01 |
| **67** | Fludarabine Phosphate (Fludara) | 101,91 | 16,46 | | **99** | Clafen (Cyclophosphamide) | 99,63 | 0,85 |
| **68** | Toremifene Citrate (Fareston, Acapodene) | 101,9 | 6,67 | | **100** | DAPT (GSI-IX) | 99,6 | 0,90 |
| **69** | Dorzolamide HCl | 101,9 | 2,66 | | **101** | SB 203580 | 99,55 | 1,02 |
| **70** | SB 431542 | 101,85 | 1,68 | | **102** | Hydroxyurea (Cytodrox) | 99,5 | 0,26 |
| **71** | LY2157299 | 101,63 | 21,72 | | **103** | MDV3100 (Enzalutamide) | 99,43 | 2,00 |
| **72** | Dalcetrapib (JTT-705) | 101,44 | 19,75 | | **104** | Isotretinoin | 99,3 | 0,74 |
| **73** | PF-3845 | 101,42 | 2,06 | | **105** | Mifepristone (Mifeprex) | 99,23 | 2,00 |
| **74** | Bicalutamide (Casodex) | 101,33 | 1,88 | | **106** | WAY-362450 | 99,22 | 0,30 |
| **75** | Chrysophanic acid (Chrysophanol) | 101,25 | 19,63 | | **107** | Temozolomide | 99,20 | 1,44 |
| **76** | Vinpocetine (Cavinton) | 101,2 | 5,41 | | **108** | D-glutamine | 99,2 | 0,20 |
| **77** | Aprepitant (MK-0869) | 101,11 | 2,28 | | **109** | Medroxyprogesterone acetate | 99,08 | 1,25 |
| **78** | S-Ruxolitinib | 101,10 | 4,40 | | **110** | TAME | 98,75 | 1,08 |
| **79** | PD153035 HCl | 101,09 | 16,54 | | **111** | Tie2 kinase inhibitor | 98,7 | 1,72 |
| **80** | Mocetinostat (MGCD0103) | 100,97 | 5,71 | | **112** | Oxaliplatin (Eloxatin) | 98,66 | 1,36 |
| **81** | Imatinib Mesylate | 100,92 | 4,05 | | **113** | Iniparib (BSI-201) | 98,46 | 2,55 |
| **82** | EX 527 | 100,8 | 3,23 | | **114** | Anagrelide HCl | 98,30 | 1,65 |
| **83** | Streptozotocin (Zanosar) | 100,76 | 33,08 | | **115** | Pamidronate Disodium | 98,29 | 1,29 |

| | | | | | | | | |
|---|---|---|---|---|---|---|---|---|
| **116** | Crenolanib (CP-868596) | 98,10 | 0,91 | | **148** | PH-797804 | 95,18 | 0,36 |
| **117** | Ubenimex (Bestatin) | 98,1 | 5,81 | | **149** | YO-01027 | 95,12 | 0,02 |
| **118** | CHIR-99021 (CT99021) HCl | 97,95 | 0,94 | | **150** | Pelitinib (EKB-569) | 95,1 | 1,53 |
| **119** | Imiquimod | 97,94 | 2,09 | | **151** | ABT-888 (Veliparib) | 94,99 | 1,85 |
| **120** | Ranolazine (Ranexa) | 97,8 | 3,67 | | **152** | Procarbazine HCl (Matulane) | 94,7 | 1,89 |
| **121** | MK-0752 | 97,77 | 1,95 | | **153** | Tandutinib (MLN518) | 94,62 | 2,50 |
| **122** | SU11274 | 97,73 | 1,32 | | **154** | PAC-1 | 94,61 | 1,79 |
| **123** | Dimesna | 97,72 | 0,82 | | **155** | Lapatinib Ditosylate (Tykerb) | 94,54 | 2,47 |
| **124** | Cyclopamine | 97,68 | 4,27 | | **156** | Flutamide (Eulexin) | 94,5 | 1,02 |
| **125** | GW4064 | 97,67 | 0,61 | | **157** | Rosiglitazone (Avandia) | 94,49 | 1,40 |
| **126** | Sodium butyrate | 97,6 | 0,56 | | **158** | Linifanib (ABT-869) | 94,42 | 5,47 |
| **127** | Tofacitinib (CP-690550, Tasocitinib) | 97,49 | 1,82 | | **159** | Leucovorin Calcium | 94,39 | 1,93 |
| **128** | AEE788 (NVP-AEE788) | 97,2 | 1,48 | | **160** | Simvastatin (Zocor) | 94,4 | 2,68 |
| **129** | Letrozole | 97,07 | 0,06 | | **161** | Masitinib (AB1010) | 94,24 | 2,30 |
| **130** | Cyclosporin A (Cyclosporine A) | 97,07 | 2,15 | | **162** | Everolimus (RAD001) | 94,21 | 4,30 |
| **131** | Canagliflozin | 97,05 | 0,83 | | **163** | BMS-599626 (AC480) | 94,06 | 2,55 |
| **132** | Ftorafur | 96,98 | 1,64 | | **164** | Gossypol | 93,98 | 1,52 |
| **133** | CAL-101 (GS-1101) | 96,79 | 0,60 | | **165** | Gefitinib (Iressa) | 93,95 | 1,86 |
| **134** | Nelarabine (Arranon) | 96,75 | 0,84 | | **166** | (-)-Epigallocatechin gallate | 93,87 | 1,38 |
| **135** | IC-87114 | 96,68 | 1,39 | | **167** | TPCA-1 | 93,83 | 2,15 |
| **136** | Megestrol Acetate | 96,44 | 0,49 | | **168** | Tamoxifen Citrate (Nolvadex) | 93,5 | 3,63 |
| **137** | Sorafenib (Nexavar) | 96,43 | 1,49 | | **169** | Lapatinib | 93,4 | 1,72 |
| **138** | Fingolimod (FTY720) | 96,36 | 1,01 | | **170** | Lonidamine | 93,26 | 0,43 |
| **139** | SB 525334 | 96,3 | 2,15 | | **171** | STF-62247 | 93,25 | 4,41 |
| **140** | Bendamustine HCL | 96,24 | 1,38 | | **172** | Pazopanib | 93,12 | 9,45 |
| **141** | Saracatinib (AZD0530) | 96,11 | 0,51 | | **173** | Nilotinib (AMN-107) | 92,79 | 0,50 |
| **142** | Evista (Raloxifene HCl) | 95,74 | 1,88 | | **174** | AG14361 | 92,8 | 1,79 |
| **143** | Thalidomide | 95,61 | 0,20 | | **175** | Carmofur | 92,24 | 2,03 |
| **144** | BMS 794833 | 95,6 | 0,22 | | **176** | NVP-BSK805 2HCl | 91,70 | 0,70 |
| **145** | Exemestane | 95,43 | 0,59 | | **177** | Nutlin-3 | 91,49 | 2,1 |
| **146** | LY2228820 | 95,3 | 0,26 | | **178** | Fulvestrant (Faslodex) | 91,20 | 1,3 |
| **147** | Tosedostat (CHR2797) | 95,2 | 3,51 | | **179** | ABT-263 (Navitoclax) | 91,15 | 3,0 |

| | | | | | | | | |
|---|---|---|---|---|---|---|---|---|
| **180** | Itraconazole (Sporanox) | 91,12 | 2,0 | | **206** | WZ4002 | 86,92 | 7,5 |
| **181** | ABT-737 | 90,77 | 1,4 | | **207** | MK-2206 2HCl | 86,43 | 0,8 |
| **182** | Ifosfamide | 90,65 | 4,8 | | **208** | Salinomycin (Procoxacin) | 85,94 | 1,7 |
| **183** | Fluvastatin sodium (Lescol) | 90,6 | 3,5 | | **209** | Tivozanib (AV-951) | 85,80 | 1,7 |
| **184** | Amuvatinib (MP-470) | 90,44 | 3,6 | | **210** | Quizartinib (AC220) | 85,2 | 1,6 |
| **185** | BMS 777607 | 90,2 | 5,2 | | **211** | CP-466722 | 84,49 | 6,6 |
| **186** | KU-60019 | 90,0 | 1,2 | | **212** | Bleomycin sulfate | 84,46 | 1,6 |
| **187** | OSI-930 | 89,81 | 1,0 | | **213** | R935788 (Fostamatinib disodium, R788 disodium) | 84,2 | 1,5 |
| **188** | PD 0332991 (Palbociclib) HCl | 89,75 | 6,3 | | **214** | Olaparib (AZD2281) | 83,88 | 1,2 |
| **0,7** | Pemetrexed (Alimta) | 89,29 | 7,1 | | **215** | CYC116 | 83,87 | 6,8 |
| **190** | OSI-420 | 89,1 | 2,5 | | **216** | Paclitaxel (Taxol) | 83,24 | 1,0 |
| **191** | Tipifarnib (Zarnestra) | 89,1 | 7,5 | | **217** | Imatinib (Gleevec) | 82,91 | 2,8 |
| **192** | AZ 3146 | 89,01 | 0,8 | | **218** | PD173074 | 82,68 | |
| **193** | Adrucil (Fluorouracil) | 88,77 | 1,5 | | **219** | Desmethyl Erlotinib (CP-473420) | 82,07 | 6,4 |
| **194** | Mycophenolate mofetil (CellCept) | 88,5 | 1,5 | | **220** | BIBF1120 (Vargatef) | 81,76 | 5,7 |
| **195** | Y-27632 2HCl | 88,41 | 10,3 | | **221** | Cyt387 | 81,0 | 1,4 |
| **196** | Abiraterone (CB-7598) | 88,33 | 4,0 | | **222** | Erlotinib HCl | 80,99 | 2,0 |
| **197** | Decitabine | 88,27 | 3,5 | | **223** | GSK2126458 | 80,52 | 6,0 |
| **198** | Rapamycin (Sirolimus) | 87,95 | 6,0 | | **224** | PIK-93 | 80,5 | 9,5 |
| **199** | R406 (free base) | 87,9 | 3,0 | | **225** | Rigosertib (ON-01910) | 80,4 | 1,4 |
| **200** | SNS-314 Mesylate | 87,86 | 6,6 | | **226** | CEP33779 | 80,15 | 8,9 |
| **201** | Dacomitinib (PF299804,PF-00299804) | 87,80 | 2,4 | | **227** | Regorafenib (BAY 73-4506) | 80,09 | 3,2 |
| **202** | Sotrastaurin (AEB071) | 87,73 | 5,2 | | **228** | SGI-1776 free base | 80,1 | 0,5 |
| **203** | Telatinib (BAY 57-9352) | 87,47 | 8,4 | | **229** | Mycophenolic (Mycophenolate) | 80,05 | 2,4 |
| **204** | Afatinib (BIBW2992) | 87,30 | 1,7 | | **230** | Enzastaurin (LY317615) | 80,04 | 4,7 |
| **205** | Deforolimus (Ridaforolimus) | 87,07 | 6,5 | | | | | |

| **80 < Viability % > 50** | | | | | | | | |
|---|---|---|---|---|---|---|---|---|
| | **Compounds** | **%** | **s.d.** | | | **Compounds** | **%** | **s.d.** |
| **231** | KX2-391 | 79,72 | 0,7 | | **233** | Docetaxel (Taxotere) | 78,86 | 1,0 |
| **232** | Neratinib (HKI-272) | 79,1 | 2,7 | | **234** | Epothilone B (EPO906) | 78,7 | 1,0 |

| | | | | | | | | |
|---|---|---|---|---|---|---|---|---|
| **235** | AMG 900 | 78,54 | 2,9 | | **268** | GSK461364 | 64,5 | 4,71 |
| **236** | Entinostat (MS-275, SNDX-275) | 78,48 | 6,6 | | **269** | TAE684 (NVP-TAE684) | 64,48 | 5,57 |
| **237** | Cediranib (AZD2171) | 78,36 | 0,8 | | **270** | Bosutinib (SKI-606) | 63,96 | 0,70 |
| **238** | PF 573228 | 77,3 | 2,8 | | **271** | Vorinostat (SAHA) | 63,84 | 3,73 |
| **239** | CX-4945 (Silmitasertib) | 77,11 | 10,5 | | **272** | GDC-0941 | 63,59 | 2,18 |
| **240** | NU7441 (KU-57788) | 76,94 | 4,1 | | **273** | SB590885 | 63,26 | 2,82 |
| **241** | TW-37 | 76,93 | 1,3 | | **274** | PD0325901 | 63,18 | 2,92 |
| **242** | ABT-751 | 76,70 | 3,7 | | **275** | BI6727 (Volasertib) | 63,18 | 0,76 |
| **243** | AZ628 | 76,09 | 6,7 | | **276** | BI 2536 | 63,17 | 2,47 |
| **244** | Epothilone A | 75,57 | 1,7 | | **277** | Raltitrexed (Tomudex) | 62,87 | 7,85 |
| **245** | Sunitinib Malate (Sutent) | 75,48 | 3,2 | | **278** | CI-1040 (PD184352) | 62,75 | 4,44 |
| **246** | Axitinib | 75,37 | 3,3 | | **279** | YM201636 | 61,83 | 1,54 |
| **247** | Danusertib (PHA-739358) | 72,73 | 7,4 | | **280** | Nocodazole | 61,77 | 4,19 |
| **248** | GSK1120212 (Trametinib) | 72,67 | 0,4 | | **281** | TG101348 (SAR302503) | 61,43 | 8,20 |
| **249** | Dasatinib (BMS-354825) | 72,25 | 4,8 | | **282** | VX-680 (MK-0457, Tozasertib) | 61,13 | 2,73 |
| **250** | Temsirolimus (Torisel) | 72,07 | 2,2 | | **283** | ZSTK474 | 59,86 | 0,46 |
| **251** | PIK-90 | 71,52 | 3,7 | | **284** | Crizotinib (PF-02341066) | 59,69 | 6,97 |
| **252** | AZD8055 | 70,3 | 7,7 | | **285** | INK 128 (MLN0128) | 59,21 | 6,61 |
| **253** | BX-795 | 70,25 | 2,8 | | **286** | SB 743921 | 59,2 | 3,55 |
| **254** | GDC-0879 | 69,28 | 1,1 | | **287** | Ponatinib (AP24534) | 58,8 | 0,22 |
| **255** | TAK-733 | 69,26 | 10,6 | | **288** | PHA-793887 | 58,2 | 5,57 |
| **256** | Elesclomol | 68,81 | 2,4 | | **289** | PF-03814735 | 57,31 | 4,49 |
| **257** | Dovitinib (TKI-258) | 68,05 | 7,9 | | **290** | Vandetanib (Zactima) | 56,93 | 1,46 |
| **258** | CH5132799 | 67,80 | 2,5 | | **291** | PI-103 | 56,56 | 0,55 |
| **259** | ENMD-2076 | 67,73 | 5,0 | | **292** | BKM120 (NVP-BKM120) | 56,35 | 2,44 |
| **260** | Torin 1 | 67,59 | 1,3 | | **293** | Geldanamycin | 55,73 | 9,00 |
| **261** | Linsitinib (OSI-906) | 67,32 | 5,3 | | **294** | SB939 (Pracinostat) | 55,6 | 2,05 |
| **262** | 2-Methoxyestradiol | 66,58 | 3,4 | | **295** | BEZ235 (NVP-BEZ235) | 54,60 | 2,98 |
| **263** | Vincristine | 66,26 | 2,2 | | **296** | Azathioprine (Azasan, Imuran) | 52,7 | 0,93 |
| **264** | AZD6244 (Selumetinib) | 66,02 | 1,2 | | **297** | Ispinesib (SB-715992) | 52,2 | 2,84 |
| **265** | JNJ-7706621 | 65,00 | 5,42 | | **298** | BIIB021 | 51,92 | 3,08 |
| **266** | Ku-0063794 | 64,98 | 1,50 | | **299** | Azacitidine (Vidaza) | 51,8 | 1,95 |
| **267** | WYE-354 | 64,90 | 4,16 | | **300** | Cytarabine | 50,2 | 0,99 |

## 50 < Viability % > 30

| | *Compounds* | *%* | *s.d.* | | | *Compounds* | *%* | *s.d.* |
|---|---|---|---|---|---|---|---|---|
| **301** | Ganetespib (STA-9090) | 49,70 | 0,71 | | **313** | Gemcitabine HCl (Gemzar) | 39,11 | 4,04 |
| **302** | Belinostat (PXD101) | 48,91 | 4,78 | | **314** | JNJ-26481585 | 38,98 | 5,87 |
| **303** | AR-42 (HDAC-42) | 47,36 | 2,95 | | **315** | Etoposide (VP-16) | 37,60 | 10,57 |
| **304** | 17-DMAG HCl (Alvespimycin) | 46,96 | 2,53 | | **316** | APO866 (FK866) | 36,56 | 3,78 |
| **305** | AUY922 (NVP-AUY922) | 46,53 | 0,66 | | **317** | Trichostatin A (TSA) | 35,67 | 0,89 |
| **306** | CUDC-101 | 45,81 | 3,32 | | **318** | Floxuridine (Fludara) | 33,35 | 6,49 |
| **307** | 17-AAG (Tanespimycin) | 45,80 | 2,47 | | **319** | Mercaptopurine | 31,71 | 4,81 |
| **308** | Vemurafenib (PLX4032) | 44,87 | 4,27 | | **320** | MLN9708 | 31,7 | 4,30 |
| **309** | Gemcitabine (Gemzar) | 44,4 | 1,18 | | **321** | AT7519 | 31,6 | 1,85 |
| **310** | Torin 2 | 42,25 | 2,53 | | **322** | PF-562271 | 30,37 | 2,37 |
| **311** | PCI-24781 | 41,63 | 0,02 | | **323** | Obatoclax mesylate | 30,24 | 5,13 |
| **312** | MK-1775 | 39,7 | 0,33 | | | | | |

## Viability % < 30

| | *Compounds* | *%* | *s.d.* | | | *Compounds* | *%* | *s.d.* |
|---|---|---|---|---|---|---|---|---|
| **324** | MLN2238 | 29,0 | 2,62 | | **333** | Triptolide | 17,94 | 5,39 |
| **325** | DCC-2036 | 28,05 | 4,47 | | **334** | Clofarabine | 16,99 | 4,25 |
| **326** | Teniposide | 27,3 | 2,60 | | **335** | Bortezomib | 16,81 | 1,90 |
| **327** | Irinotecan HCl | 26.9 | 0,42 | | **336** | Irinotecan | 16,25 | 1,98 |
| **340** | SRT1720 | 26.50 | 1,09 | | **337** | Cladribine | 14,42 | 2,21 |
| **328** | AT9283 | 26.45 | 3,27 | | **338** | AZD7762 | 12,3 | 4,72 |
| **329** | Abitrexate | 26.10 | 4,41 | | **339** | WP1130 | 8,86 | 0,35 |
| **330** | LY2603618 | 20,77 | 4,93 | | **341** | Mitoxantrone HCl | 5,75 | 1,85 |
| **331** | Flavopiridol HCl | 18,17 | 1,40 | | **342** | Daunorubicin HCl | 3,96 | 3,37 |
| **332** | SNS-032 | 18,05 | 3,17 | | **343** | Topotecan HCl | 3,89 | 1,33 |
| **344** | LDN193189 | 3,40 | 6,92 | | **347** | YM155 | 1,82 | 4,15 |
| **345** | Doxorubicin | 3,35 | 0,20 | | **348** | Idarubicin HCl | 1,64 | 1,08 |
| **346** | Epirubicin HCl | 2,23 | 0,67 | | **349** | PIK-75 | 1,33 | 3,85 |

| Viability at 48 hours | | | | | | | | |
|---|---|---|---|---|---|---|---|---|
| 100 < Viability % > 80 | | | | | | | | |
| | ***Compounds*** | **%** | **s.d.** | | | ***Compounds*** | **%** | **s.d.** |
| **1** | Valproic acid sodium salt (Sodium valproate) | 118.03 | 1.57 | | **28** | S-Ruxolitinib | 102.48 | 0.62 |
| **2** | E7080 (Lenvatinib) | 116.62 | 0.99 | | **29** | SB 431542 | 102.45 | 0.65 |
| **3** | Aprepitant (MK-0869) | 115.83 | 1.81 | | **30** | Roscovitine (Seliciclib, CYC202) | 102.43 | 0.84 |
| **4** | Cyclopamine | 114.93 | 0.79 | | **31** | BAY 11-7082 (BAY 11-7821) | 102.12 | 4.39 |
| **5** | Capecitabine (Xeloda) | 114.19 | 2.95 | | **32** | EX 527 | 102.0 | 4.0 |
| **6** | Cisplatin | 113.12 | 0.51 | | **33** | Hydrocortisone (Cortisol) | 101.9 | 2.5 |
| **7** | Bicalutamide (Casodex) | 111.95 | 4.16 | | **34** | Dorzolamide HCl | 101.9 | 1.3 |
| **8** | Ostarine (MK-2866) | 111.19 | 0.60 | | **35** | SB 203580 | 101.80 | 2.59 |
| **9** | Andarine (GTX-007) | 109.75 | 0.05 | | **36** | RG108 | 101.75 | 0.23 |
| **10** | SU11274 | 108.58 | 0.50 | | **37** | Lonidamine | 101.48 | 0.31 |
| **11** | WZ4002 | 107.80 | 1.17 | | **38** | BIRB 796 (Doramapimod) | 101.3 | 3.5 |
| **12** | Anastrozole | 106.60 | 4.98 | | **39** | Toremifene Citrate (Fareston, Acapodene) | 101.3 | 3.6 |
| **13** | Zibotentan (ZD4054) | 106.4 | 3.1 | | **40** | Saracatinib (AZD0530) | 101.2 | 5.45 |
| **14** | SB 216763 | 105.31 | 1.25 | | **41** | DAPT (GSI-IX) | 101.1 | 2.6 |
| **15** | Busulfan (Myleran, Busulfex) | 104.9 | 7.5 | | **42** | DMXAA (ASA404) | 101.1 | 1.3 |
| **16** | Anagrelide HCl | 104.67 | 0.97 | | **43** | Formestane | 101.1 | 4.5 |
| **17** | Febuxostat (Uloric) | 104.5 | 1.5 | | **44** | Rosiglitazone (Avandia) | 101.05 | 3.09 |
| **18** | Iniparib (BSI-201) | 104.31 | 0.49 | | **45** | Sirtinol | 101.0 | 1.54 |
| **19** | Doxercalciferol (Hectorol) | 104.2 | 1.4 | | **46** | Zileuton | 101.0 | 0.6 |
| **20** | Evista (Raloxifene HCl) | 103.90 | 1.32 | | **47** | BIBF1120 (Vargatef) | 100.97 | 3,42 |
| **21** | Celecoxib | 103.69 | 1.43 | | **48** | XL765 (SAR245409) | 100.9 | 1.7 |
| **22** | Tofacitinib (CP-690550, Tasocitinib) | 103.5 | 0.45 | | **49** | AEE788 (NVP-AEE788) | 100.8 | 4.1 |
| **23** | Cediranib (AZD2171) | 103.33 | 0.45 | | **50** | Dexamethasone | 100.8 | 2.4 |
| **24** | Vismodegib (GDC-0449) | 103.13 | 0.71 | | **51** | SGX-523 | 100.67 | 0.36 |
| **25** | Dexamethasone acetate | 102.84 | 1.72 | | **52** | Ranolazine (Ranexa) | 100.6 | 3.1 |
| **26** | Lapatinib Ditosylate (Tykerb) | 102.69 | 2.25 | | **53** | Oxaliplatin (Eloxatin) | 100.47 | 1.72 |
| **27** | Brivanib (BMS-540215) | 102.56 | 2.56 | | **54** | (-)-Epigallocatechin gallate | 100.44 | 2.21 |

| | | | | | | | | |
|---|---|---|---|---|---|---|---|---|
| **55** | Betapar (Meprednisone) | 100.2 | 3.1 | | **86** | Lapatinib | 97.9 | 13.1 |
| **56** | MDV3100 (Enzalutamide) | 100.19 | 1.44 | | **87** | Maraviroc | 97.8 | 6.0 |
| **57** | Hydroxyurea (Cytodrox) | 100.2 | 1.0 | | **88** | PCI-32765 (Ibrutinib) | 97.74 | 2.92 |
| **58** | Dacarbazine (DTIC-Dome) | 100.13 | 0.04 | | **89** | Telatinib (BAY 57-9352) | 97.70 | 2.11 |
| **59** | Cyclophosphamide monohydrate | 100.1 | 3.2 | | **90** | Chrysophanic acid (Chrysophanol) | 97.61 | 1.81 |
| **60** | Imatinib Mesylate | 100.08 | 0.75 | | **91** | BMS 794833 | 97.6 | 2.1 |
| **61** | BMS-599626 (AC480) | 99.92 | 1.98 | | **92** | ABT-888 (Veliparib) | 97.55 | 2.32 |
| **62** | SGI-1776 free base | 99.8 | 2.7 | | **93** | Carboplatin | 97.50 | 0.17 |
| **63** | Masitinib (AB1010) | 99.76 | 1.29 | | **94** | Bendamustine HCL | 97.46 | 0.49 |
| **64** | Dalcetrapib (JTT-705) | 99.69 | 3.03 | | **95** | TAME | 97.45 | 1.10 |
| **65** | Quercetin (Sophoretin) | 99.67 | 0.82 | | **96** | GW4064 | 97.44 | 4.73 |
| **66** | Estradiol | 99.6 | 1.5 | | **97** | Mifepristone (Mifeprex) | 97.23 | 1.49 |
| **67** | Y-27632 2HCl | 99.54 | 1.84 | | **98** | Tamoxifen Citrate (Nolvadex) | 97.2 | 2.9 |
| **68** | BIBR 1532 | 99.39 | 1.35 | | **99** | Mesna (Uromitexan, Mesnex) | 97.0 | 8.3 |
| **69** | Ruxolitinib (INCB018424) | 99.3 | 6.9 | | **100** | Phloretin (Dihydronaringenin) | 96.92 | 2.22 |
| **70** | 3-Methyladenine | 99.04 | 1.2 | | **101** | Canagliflozin | 96.91 | 1.19 |
| **71** | Nelarabine (Arranon) | 99.0 | 0.55 | | **102** | Medroxyprogesterone acetate | 96.76 | 1.61 |
| **72** | Fingolimod (FTY720) | 98.95 | 1.04 | | **103** | AG14361 | 96.6 | 5.1 |
| **73** | Barasertib (AZD1152-HQPA) | 98.91 | 1.02 | | **104** | Procarbazine HCl (Matulane) | 96.4 | 5.0 |
| **74** | Letrozole | 98.88 | 2.63 | | **105** | Isotretinoin | 96.4 | 8.2 |
| **75** | Tandutinib (MLN518) | 98.86 | 1.39 | | **106** | Abiraterone (CB-7598) | 96.35 | 3.92 |
| **76** | JNJ-38877605 | 98.84 | 4.45 | | **107** | Nilotinib (AMN-107) | 96.23 | 2.43 |
| **77** | Dimesna | 98.82 | 2.82 | | **108** | D-glutamine | 96.2 | 9.0 |
| **78** | Sodium butyrate | 98.7 | 1.6 | | **109** | GSK690693 | 96.15 | 0.72 |
| **79** | Estrone | 98.7 | 7.6 | | **110** | Triamcinolone Acetonide | 96.0 | 6.9 |
| **80** | Motesanib Diphosphate (AMG-706) | 98.37 | 3.89 | | **111** | Pioglitazone (Actos) | 95.96 | 1.93 |
| **81** | STF-62247 | 98.26 | 0.54 | | **112** | Lomustine (CeeNU) | 95.9 | 2.8 |
| **82** | LY2157299 | 98.16 | 0.71 | | **113** | LY2109761 | 95.76 | 2.27 |
| **83** | PD153035 HCl | 98.06 | 0.51 | | **114** | Lenalidomide (Revlimid) | 95.43 | 0.48 |
| **84** | PHA-665752 | 98.03 | 1.03 | | **115** | Linifanib (ABT-869) | 95.39 | 0.99 |
| **85** | Clafen (Cyclophosphamide) | 98.0 | 2.07 | | **116** | Exemestane | 95.36 | 0.18 |

| | | | | | | | | |
|---|---|---|---|---|---|---|---|---|
| **117** | Imiquimod | 95.35 | 2.21 | | **150** | SB 525334 | 92.3 | 11.7 |
| **118** | Megestrol Acetate | 95.23 | 0.12 | | **151** | Ftorafur | 92.18 | 1.81 |
| **119** | Sotrastaurin (AEB071) | 95.20 | 2.35 | | **152** | Dapagliflozin | 92.0 | 2.6 |
| **120** | Coenzyme Q10 (CoQ10) | 95.17 | 4.60 | | **153** | CYC116 | 91.97 | 3.23 |
| **121** | Streptozotocin (Zanosar) | 95.05 | 0.17 | | **154** | Altretamine (Hexalen) | 91.97 | 3.23 |
| **122** | AT-406 | 94.82 | 1.55 | | **155** | PF-04217903 | 91.58 | 2.18 |
| **123** | Pamidronate Disodium | 94.78 | 1.73 | | **156** | YO-01027 | 91.21 | 1.10 |
| **124** | Vinblastine | 94.77 | 5.34 | | **157** | MK-0752 | 90.87 | 2.06 |
| **125** | IC-87114 | 94.76 | 1.94 | | **158** | Fulvestrant (Faslodex) | 90.5 | 2.59 |
| **126** | Vatalanib 2HCl (PTK787) | 94.69 | 4.50 | | **159** | PIK-93 | 90.4 | 2.4 |
| **127** | GW3965 HCl | 94.55 | 1.47 | | **160** | Imatinib (Gleevec) | 90.38 | 2.20 |
| **128** | Ubenimex (Bestatin) | 94.5 | 5.6 | | **161** | Gefitinib (Iressa) | 90.15 | 1.06 |
| **129** | Quizartinib (AC220) | 94.3 | 1.3 | | **162** | Enzastaurin (LY317615) | 90.07 | 20.50 |
| **130** | Pomalidomide | 94.2 | 1.1 | | **163** | Cyclosporin A (Cyclosporine A) | 89.98 | 1.57 |
| **131** | TPCA-1 | 94.13 | 0.27 | | **164** | MK-2206 2HCl | 89.96 | 1.73 |
| **132** | Flutamide (Eulexin) | 94.1 | 3.2 | | **165** | Temozolomide | 89.64 | 5.03 |
| **133** | ABT-737 | 94.08 | 1.84 | | **166** | Amuvatinib (MP-470) | 89.63 | 2.76 |
| **134** | Tie2 kinase inhibitor | 94.0 | 5.1 | | **167** | R406 (free base) | 89.3 | 3.6 |
| **135** | Bexarotene | 93.6 | 4.9 | | **168** | SNS-314 Mesylate | 89.20 | 3.07 |
| **136** | CAL-101 (GS-1101) | 93.6 | 0.79 | | **169** | KU-55933 | 89.16 | 7.13 |
| **137** | Vinpocetine (Cavinton) | 93.6 | 17.3 | | **170** | Tosedostat (CHR2797) | 89.2 | 2.4 |
| **138** | Pazopanib | 93.52 | 2.39 | | **171** | PH-797804 | 89.14 | 1.87 |
| **139** | Aminoglutethimide (Cytadren) | 93.5 | 3.1 | | **172** | XAV-939 | 88.68 | 2.72 |
| **140** | A-769662 | 93.47 | 0.62 | | **173** | LDE225 (NVP-LDE225, Erismodegib) | 87.9 | 6.0 |
| **141** | Thalidomide | 93.29 | 0.19 | | **174** | CP-466722 | 87.18 | 1.01 |
| **142** | Nutlin-3 | 93.26 | 6.02 | | **175** | LY294002 | 86.58 | 3.39 |
| **143** | Palomid 529 | 93.25 | 1.58 | | **176** | Tivozanib (AV-951) | 86.37 | 2.27 |
| **144** | WAY-362450 | 92.94 | 2.78 | | **177** | Afatinib (BIBW2992) | 85.95 | 0.06 |
| **145** | Ifosfamide | 92.71 | 0.46 | | **178** | OSI-420 | 85.9 | 4.2 |
| **146** | Disulfiram (Antabuse) | 92.5 | 6.5 | | **179** | Tipifarnib (Zarnestra) | 85.7 | 1.6 |
| **147** | Tretinoin (Aberela) | 92.5 | 4.5 | | **180** | Sorafenib (Nexavar) | 84.87 | 9.03 |
| **148** | NVP-BSK805 2HCl | 92.35 | 2.52 | | **181** | Prednisone (Adasone) | 84.7 | 0.2 |
| **149** | Ezetimibe (Zetia) | 92.3 | 0.4 | | **182** | CEP33779 | 84.42 | 0.92 |

| | | | | | | | | |
|---|---|---|---|---|---|---|---|---|
| **183** | Olaparib (AZD2281) | 84.10 | 1.83 | | **190** | Deforolimus (Ridaforolimus) | 81.38 | 0.1 |
| **184** | Desmethyl Erlotinib (CP-473420) | 83.98 | 8.70 | | **191** | BMS 777607 | 81.1 | 5.4 |
| **185** | Pelitinib (EKB-569) | 83.7 | 0.5 | | **192** | R935788 (Fostamatinib disodium, R788 disodium) | 81.1 | 3.4 |
| **186** | Crenolanib (CP-868596) | 83.27 | 1.87 | | **193** | PD173074 | 81.01 | 2.81 |
| **187** | Dacomitinib (PF299804,PF-00299804) | 83.25 | 3.24 | | **194** | Leucovorin Calcium | 80.57 | 3.68 |
| **188** | BTZ043 racemate | 83.18 | 0.5 | | **195** | PF-3845 | 80.18 | 1.72 |
| **189** | Itraconazole (Sporanox) | 82.29 | 3.49 | | | | | |
| | | | | | | | | |
| **80 < Viability % > 50** | | | | | | | | |
| | ***Compounds*** | **%** | **s.d.** | | | ***Compounds*** | **%** | **s.d.** |
| **196** | Rapamycin (Sirolimus) | 79.40 | 5.97 | | **216** | Docetaxel (Taxotere) | 73.13 | 1.87 |
| **197** | OSI-930 | 79.17 | 1.73 | | **217** | AZ 3146 | 71.91 | 5.37 |
| **198** | JNJ 26854165 (Serdemetan) | 79.06 | 13.07 | | **218** | AMG 900 | 71.43 | 7.93 |
| **199** | Elesclomol | 78.63 | 1.07 | | **219** | Paclitaxel (Taxol) | 71.0 | 2.24 |
| **200** | KU-60019 | 78.4 | 2.4 | | **220** | PF 573228 | 70.9 | 1.2 |
| **201** | MLN8237 (Alisertib) | 78.37 | 1.76 | | **221** | XL147 | 70.67 | 1.27 |
| **202** | Erlotinib HCl | 77.74 | 3.19 | | **222** | Axitinib | 70.25 | 0.78 |
| **203** | Temsirolimus (Torisel) | 77.5 | 0.88 | | **223** | Simvastatin (Zocor) | 68.7 | 1.0 |
| **204** | Rucaparib (AG-014699 , PF-01367338) | 77.38 | 2.24 | | **224** | LY2228820 | 68.6 | 2.1 |
| **205** | Salinomycin (Procoxacin) | 76.93 | 0.71 | | **225** | Adrucil (Fluorouracil) | 68.44 | 2.18 |
| **206** | Decitabine | 76.45 | 1.67 | | **226** | CX-4945 (Silmitasertib) | 67.95 | 0.57 |
| **207** | Neratinib (HKI-272) | 76.2 | 6.1 | | **227** | Bleomycin sulfate | 67.59 | 2.44 |
| **208** | ABT-263 (Navitoclax) | 76.13 | 5.47 | | **228** | Bosutinib (SKI-606) | 67.56 | 3.77 |
| **209** | Fludarabine (Fludara) | 75.7 | 6.3 | | **229** | NU7441 (KU-57788) | 67.39 | 1.94 |
| **210** | Dasatinib (BMS-354825) | 75.29 | 3.22 | | **230** | Gossypol | 67.20 | 0.82 |
| **211** | Cyt387 | 74.3 | 4.2 | | **231** | Carmofur | 67.16 | 3.50 |
| **212** | Sunitinib Malate (Sutent) | 74.21 | 11.82 | | **232** | ENMD-2076 | 66.93 | 1.32 |
| **213** | Everolimus (RAD001) | 74.19 | 3.28 | | **233** | VX-680 (MK-0457, Tozasertib) | 65.70 | 0.01 |
| **214** | GSK1904529A | 73.69 | 1.68 | | **234** | CHIR-99021 (CT99021) HCl | 65.03 | 5.27 |
| **215** | PD 0332991 (Palbociclib) HCl | 73.18 | 0.37 | | **235** | Linsitinib (OSI-906) | 64.18 | 2.0 |

| | | | | | | | | |
|---|---|---|---|---|---|---|---|---|
| **236** | Triciribine (Triciribine phosphate) | 63.68 | 3.40 | | **244** | Crizotinib (PF-02341066) | 56.90 | 2.63 |
| **237** | PIK-90 | 63.42 | 1.53 | | **245** | TG101348 (SAR302503) | 55.92 | 5.60 |
| **238** | Regorafenib (BAY 73-4506) | 61.49 | 1.73 | | **246** | Fluvastatin sodium (Lescol) | 55.1 | 2.2 |
| **239** | PAC-1 | 61.38 | 1.62 | | **247** | YM201636 | 54.67 | 0.06 |
| **240** | Dovitinib (TKI-258) | 60.78 | 5.58 | | **248** | Mycophenolate mofetil (CellCept) | 52.9 | 2.3 |
| **241** | SB590885 | 59.09 | 3.36 | | **249** | Epothilone A | 51.72 | 2.44 |
| **242** | BX-795 | 58.75 | 0.48 | | **250** | Vandetanib (Zactima) | 50.59 | 3.99 |
| **243** | Epothilone B (EPO906) | 57.2 | 0.4 | | | | | |

## 50 < Viability % > 30

| | ***Compounds*** | ***%*** | ***s.d.*** | | | ***Compounds*** | ***%*** | ***s.d.*** |
|---|---|---|---|---|---|---|---|---|
| **251** | Vorinostat (SAHA) | 49.71 | 1.18 | | **270** | BI6727 (Volasertib) | 43.60 | 0.55 |
| **252** | GSK2126458 | 49.54 | 0.78 | | **271** | PF-03814735 | 41.69 | 9.46 |
| **253** | CH5132799 | 47.27 | 2.31 | | **272** | Torin 1 | 40.56 | 1.61 |
| **254** | AZD8055 | 46.7 | 2.3 | | **273** | TW-37 | 40.44 | 3.95 |
| **255** | Mycophenolic (Mycophenolate) | 46.62 | 1.07 | | **274** | ZSTK474 | 39.92 | 1.62 |
| **256** | Pemetrexed (Alimta) | 46.46 | 2.27 | | **275** | GSK1120212 (Trametinib) | 38.39 | 1.17 |
| **257** | Danusertib (PHA-739358) | 45.61 | 1.77 | | **276** | CI-1040 (PD184352) | 37.76 | 1.31 |
| **258** | TAK-733 | 45.48 | 3.05 | | **277** | Etoposide (VP-16) | 37.37 | 0.54 |
| **259** | Fludarabine Phosphate (Fludara) | 45.48 | 1.07 | | **278** | INK 128 (MLN0128) | 37.26 | 1.93 |
| **260** | GDC-0941 | 45.21 | 1.74 | | **279** | AZD6244 (Selumetinib) | 37.18 | 1.52 |
| **261** | Entinostat (MS-275, SNDX-275) | 45.11 | 1.28 | | **280** | Azacitidine (Vidaza) | 36.9 | 0.9 |
| **262** | 2-Methoxyestradiol | 44.93 | 0.76 | | **281** | PI-103 | 36.59 | 1.37 |
| **263** | Ponatinib (AP24534) | 44.9 | 1.2 | | **282** | AZ628 | 36.45 | 0.52 |
| **264** | WYE-354 | 44.45 | 3.77 | | **283** | Rigosertib (ON-01910) | 35.3 | 1.6 |
| **265** | TAE684 (NVP-TAE684) | 44.08 | 3.04 | | **284** | MK-1775 | 35.0 | 5.2 |
| **266** | GDC-0879 | 43.89 | 2.1 | | **285** | KX2-391 | 34.44 | 0.54 |
| **267** | ABT-751 | 43.81 | 4.79 | | **286** | PD0325901 | 34.01 | 0.97 |
| **268** | BI 2536 | 43.74 | 0.03 | | **287** | BEZ235 (NVP-BEZ235) | 30.92 | 0.31 |
| **269** | Ku-0063794 | 43.69 | 3.01 | | **288** | Nocodazole | 30.73 | 2.38 |

| Viability % < 30 | | | | | | | | |
|---|---|---|---|---|---|---|---|---|
| | ***Compounds*** | ***%*** | ***s.d.*** | | | ***Compounds*** | ***%*** | |
| **289** | SB 743921 | 28.9 | 15.6 | | **320** | Mocetinostat (MGCD0103) | 6.87 | 2.58 |
| **290** | Azathioprine (Azasan, Imuran) | 28.6 | 1.8 | | **321** | APO866 (FK866) | 6.84 | 0.02 |
| **291** | Torin 2 | 28.56 | 0.5 | | **322** | Gemcitabine HCl (Gemzar) | 6.28 | 0.76 |
| **292** | Vincristine | 27.79 | 3.9 | | **323** | SB939 (Pracinostat) | 6.0 | 1.9 |
| **293** | BKM120 (NVP-BKM120) | 27.02 | 1.1 | | **324** | Clofarabine | 4.61 | 0.2 |
| **294** | PHA-793887 | 23.4 | 1.6 | | **325** | Belinostat (PXD101) | 4.09 | 1.43 |
| **295** | CUDC-101 | 23.07 | 0.18 | | **326** | DCC-2036 | 3.87 | 0.4 |
| **296** | Vemurafenib (PLX4032) | 21.96 | 0.34 | | **327** | AR-42 (HDAC-42) | 3.42 | 0.08 |
| **297** | BIIB021 | 20.30 | 4.97 | | **328** | Cladribine | 3.14 | 0.72 |
| **298** | Abitrexate | 19.20 | 0.14 | | **329** | WP1130 | 2.92 | 0.63 |
| **299** | 17-AAG (Tanespimycin) | 18.91 | 6.72 | | **330** | AT7519 | 2.51 | 0.6 |
| **300** | Ganetespib (STA-9090) | 18.36 | 1.01 | | **331** | SRT1720 | 2.5 | 4.53 |
| **301** | Irinotecan HCl | 17.9 | 1.3 | | **332** | PCI-24781 | 2.29 | 2.24 |
| **302** | AUY922 (NVP-AUY922) | 16.03 | 5.24 | | **333** | SNS-032 | 20.4 | 1.29 |
| **303** | Obatoclax mesylate | 15.98 | 8.6 | | **334** | MLN2238 | 1.7 | 0.2 |
| **304** | Teniposide | 15.6 | 3.7 | | **335** | MLN9708 | 1.6 | 0.2 |
| **305** | Mercaptopurine | 15.36 | 1.32 | | **336** | Trichostatin A (TSA) | 1.48 | 6.66 |
| **306** | 17-DMAG HCl (Alvespimycin) | 14.33 | 8.12 | | **337** | Doxorubicin | 1.37 | 0.98 |
| **307** | Geldanamycin | 13.91 | 1.97 | | **338** | Bortezomib | 1.29 | 8.5 |
| **308** | JNJ-7706621 | 13.28 | 12.50 | | **339** | Mitoxantrone HCl | 1.23 | 0.05 |
| **309** | Irinotecan | 12.29 | 0.02 | | **340** | Flavopiridol HCl | 1.20 | 0.35 |
| **310** | PF-562271 | 12.12 | 5.05 | | **341** | PIK-75 | 1.18 | 1.10 |
| **311** | GSK461364 | 10.8 | 0.2 | | **342** | Daunorubicin HCl | 1.12 | 0.09 |
| **312** | LY2603618 | 10.55 | 0.47 | | **343** | Triptolide | 1.04 | 0.03 |
| **313** | Raltitrexed (Tomudex) | 10.06 | 0.9 | | **344** | JNJ-26481585 | 0.97 | 5.84 |
| **314** | Cytarabine | 10.0 | 0.3 | | **345** | YM155 | 0.91 | 3.25 |
| **315** | Ispinesib (SB-715992) | 9.8 | 1.6 | | **346** | LDN193189 | 0.87 | 0.02 |
| **316** | Floxuridine (Fludara) | 8.57 | 3.99 | | **347** | Idarubicin HCl | 0.84 | 0.78 |
| **317** | AT9283 | 8.25 | 0.85 | | **348** | Epirubicin HCl | 0.7 | 0.17 |
| **318** | Gemcitabine (Gemzar) | 8.0 | 1.1 | | **349** | Topotecan HCl | 0.65 | 0.15 |
| **319** | AZD7762 | 7.1 | 0.6 | | | | | |
